# Reovirus μ2 protein modulates host cell alternative splicing by reducing protein levels of U5 snRNP core components

**DOI:** 10.1093/nar/gkac272

**Published:** 2022-04-30

**Authors:** Simon Boudreault, Mathieu Durand, Carole-Anne Martineau, Jean-Pierre Perreault, Guy Lemay, Martin Bisaillon

**Affiliations:** Département de biochimie et de génomique fonctionnelle, Faculté de médecine et des sciences de la santé, Université de Sherbrooke, Sherbrooke, Québec J1E 4K8, Canada; Plateforme de RNomique, Université de Sherbrooke, Sherbrooke, Québec J1E 4K8, Canada; Département de biochimie et de génomique fonctionnelle, Faculté de médecine et des sciences de la santé, Université de Sherbrooke, Sherbrooke, Québec J1E 4K8, Canada; Département de biochimie et de génomique fonctionnelle, Faculté de médecine et des sciences de la santé, Université de Sherbrooke, Sherbrooke, Québec J1E 4K8, Canada; Département de microbiologie, infectiologie et immunologie, Faculté de médecine, Université de Montréal, Montréal, Québec H3C 3J7, Canada; Département de biochimie et de génomique fonctionnelle, Faculté de médecine et des sciences de la santé, Université de Sherbrooke, Sherbrooke, Québec J1E 4K8, Canada

## Abstract

Mammalian orthoreovirus (MRV) is a double-stranded RNA virus from the *Reoviridae* family presenting a promising activity as an oncolytic virus. Recent studies have underlined MRV’s ability to alter cellular alternative splicing (AS) during infection, with a limited understanding of the mechanisms at play. In this study, we investigated how MRV modulates AS. Using a combination of cell biology and reverse genetics experiments, we demonstrated that the *M1* gene segment, encoding the μ2 protein, is the primary determinant of MRV’s ability to alter AS, and that the amino acid at position 208 in μ2 is critical to induce these changes. Moreover, we showed that the expression of μ2 by itself is sufficient to trigger AS changes, and its ability to enter the nucleus is not required for all these changes. Moreover, we identified core components of the U5 snRNP (i.e. EFTUD2, PRPF8, and SNRNP200) as interactors of μ2 that are required for MRV modulation of AS. Finally, these U5 snRNP components are reduced at the protein level by both MRV infection and μ2 expression. Our findings identify the reduction of U5 snRNP components levels as a new mechanism by which viruses alter cellular AS.

## Graphical Abstract

**Graphical Abstract:**
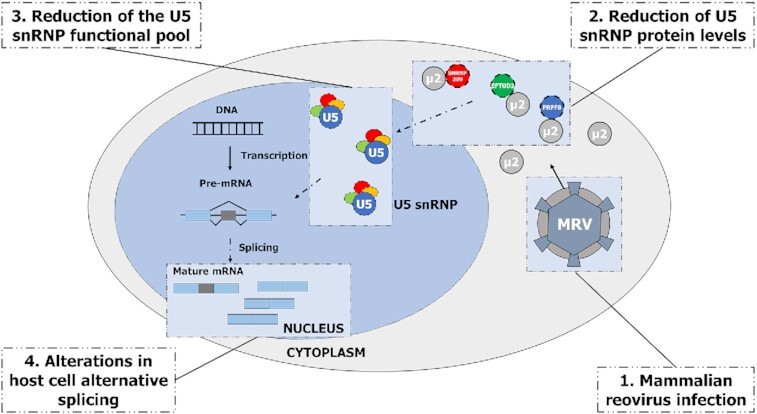
Upon reovirus infection, the viral μ2 protein exerts its activity on cellular AS by reducing the levels of U5 snRNP core components.

## INTRODUCTION

Mammalian orthoreovirus (MRV) is a double-stranded (dsRNA) virus from the *Reoviridae* family which has been instrumental to our understanding of the basis of virus replication, such as internalization, uncoating, transcription, and translation (1–3). MRV genome is composed of ten dsRNA segments that produce eight structural proteins (λ1, λ2, λ3, μ1, μ2, σ1, σ2, σ3) that form both the outer and the inner capsid (or core), and four non-structural proteins (μNS, μNSC, σNS and σ1s) involved in replication (4). MRV replication happens in cytoplasmic inclusions named viral factories (VF), which are structures acting as organizing centers to coordinate translation of viral mRNA, genome replication, gene segment assortment, genome packaging, and assembly of newly produced viral particles (5,6). VF are formed primarily by the non-structural protein μNS, but μ2 and σNS are also necessary for their genesis and maturation (7,8). In VF, μ2 binds to both μNS and cellular microtubules, and thus anchors VF to cellular microtubules (7–9). The μ2 protein is a 83 kDa structural protein encoded by the M1 segment, and a minor component of the core (10). The μ2 protein possesses both ssRNA- and dsRNA-binding activities (11); nucleoside triphosphatase (NTPase) and RNA 5′-triphosphatase (RTPase) enzymatic activities *in vitro* (12), and can also form homodimers (13). Interest in MRV has exploded since the discovery of its natural ability to preferentially replicate in and destroy cancer cells, making it one of the few naturally oncolytic viruses (14). Despite initial promises, clinical trials have not been as successful as hoped, suggesting that improvement to WT MRV might enhance its oncolytic potential (15). Notably, polymorphisms in μ2 have been linked to the MRV’s oncolytic potential (16).

The interferon (IFN) pathway is the main cellular response to fight viral infection and alert the immune system (17). Viral determinants, such as dsRNA, are recognized by pattern-recognition receptors (e.g. RIG-I), triggering a signaling cascade leading to the production of IFN, key components of the innate immune response to fight viral infection. Once secreted, IFN can act on uninfected cells in a paracrine fashion to shield them from infection, or on the infected cell in an autocrine manner to help them fight the virus. The binding of IFN to its receptor induces the expression of a myriad of interferon-stimulated genes (ISG), which produces the effectors of the cellular antiviral response (17,18). This pathway is notably dysregulated in cancer cells (19,20), and is involved in the ability of certain viruses to infect and kill them preferentially (21–23).

Upon transcription of the RNA in the nucleus, eukaryotic cells need to process pre-mature RNA through numerous steps before exporting them into the cytoplasm for translation. Amongst those maturation processes, constitutive splicing allows the removal of non-coding introns, and ligation of the coding exons in the mature mRNA. The spliceosome is a large ribonucleoprotein (RNP) complex assembled from five small nuclear ribonucleoproteins complexes (snRNP; U1, U2, U4, U5 and U6) responsible for recognizing key sequences in introns (i.e. branch point and polypyrimidine tract) and exons (5′ splice site and 3′ splice site) to catalyze the removal of introns. These snRNP are recruited to the intron in a sequential fashion, culminating in the reorganization of the precatalytic spliceosome and formation of an activated B complex committed to excising the intron (reviewed in (24)). Remarkably, the U5 snRNP plays a critical role in the reorganization and in the subsequent steps allowing the removal of the intron (25–27). On the other hand, alternative splicing (AS) results in the formation of a mixed populations of mature mRNAs (28–31). AS arises from stimulatory and inhibitory signals coming from multiple splicing factors bound to pre-mature RNA near weak splice sites, either helping or destabilizing spliceosome assembly at this location. This allows for the removal of exon or part of exons, and introns to be retained in the mRNA, altering the coding potential of the RNA. AS is a pivotal RNA processing step to allow increased protein diversity since mRNA arising from the same gene can encode different isoforms of the same protein. These isoforms can be differentially regulated through the inclusion of specific domains, and thus help the cell fine-tune the levels and activity of its proteins (32–34). Notably, numerous proteins involved in the innate immune response, such as IRF7, are regulated through their AS (34–36).

Many viruses usurp the cellular splicing machinery to splice their own genes and increase protein diversity (37). However, the impact of viruses on the AS landscape of their host cells has been overlooked until recently. This field has been rapidly expanding in the last 10 years, with mounting evidence showing that viral infection indeed impacts the AS of the infected cell (38–41); reviewed in (24,42,43). Previously, we and others have demonstrated that MRV infection induces drastic changes in cellular AS (44,45). For example, infection of murine L929 fibroblasts with MRV leads to a dysregulation in 240 alternative splicing events (ASE) at 16 h post-infection (PI) (44). This modulation of cellular AS is an entirely novel actor in MRV-host interaction, and these changes in AS have the potential to reshape the proteome of infected cells. Moreover, cancer cells present dysregulated AS compared to normal cells, and it is thus tempting to speculate that MRV modulation of AS could be involved in the specificity of the virus towards cancer cells or its ability to destroy them preferentially.

In the present study, we investigated the mechanism used by MRV to induce changes in cellular AS during infection. Using a combination of cell biology, reverse genetics experiments, AS minigene reporter assays, and IP-MS, we demonstrated that the MRV μ2 protein is the main determinant of MRV modulation of AS, and interacts with core components of the U5 snRNP. These U5 components are required for MRV modulation of AS and are reduced at the protein level during MRV infection. Our findings identify this reduction of U5 snRNP components as a new mechanism by which viruses alter cellular AS during infection.

## MATERIALS AND METHODS

### Cells and viruses

Murine L929 fibroblasts were originally obtained from the American Type Culture Collection (ATCC). The baby hamster kidney (BHK) cell line stably expressing the T7 RNA polymerase (BSR-T7 cells) has been described (46) and was a generous gift from the laboratory of Dr John Hiscott (Lady Davis Research Institute, Montréal, Canada). The Vero cell line was obtained from the laboratory of Dr Lee-Hwa Tai (Université de Sherbrooke, Sherbrooke, Canada). The COS-7 cell line was acquired from the laboratory of Dr Xavier Roucou (Université de Sherbrooke, Sherbrooke, Canada). The 293T cell line was a generous gift from the laboratory of Dr. Nathalie Rivard (Université de Sherbrooke, Sherbrooke, Canada). L929, Vero and BHK-T7 cells were routinely grown in Eagle's minimal essential medium (EMEM, Wisent) containing 5% fetal bovine serum (Wisent) and supplemented in 1% glutamine; 293T and COS-7 cells were routinely grown in Dulbecco's modified Eagle's medium (DMEM, Wisent) containing 10% fetal bovine serum (Wisent). MRV serotype 3 strain Dearing (T3/Human/Ohio/Dearing/55) was also originally obtained from ATCC and was propagated and titrated by TCID_50_ on L929 fibroblasts, as routinely used in the laboratory (47). The WT laboratory stock of MRV type 3 (T3D^S^) was previously described (48,49), and rescued by reverse genetics following the introduction of the appropriate mutations in the plasmids encoding the WT virus from the original reverse genetics system, T3D^K^ (50). T3D^K^ was rescued using the original reverse genetics system. Other viruses, harboring various combinations of genes from T3D^K^ or T3D^S^ in either background, were obtained by reverse genetics, as described below.

### Viral infection

L929 cells were plated at a density of 7 × 10^4^ cells/cm^2^ the day before infection at a multiplicity of infection (MOI) of 50 TCID_50_ units per cell using standard procedures (47). Control L929 cells were seeded at the same density and mock infected. For AS-PCR experiments described below, cells were collected 16 h post-infection, at which time visible cytopathic effect was still minimal, with the exception of the time course experiment where RNA was harvested at indicated times.

### Bystander experiment

L929 cells were plated on 0.4 μm pore-diameter Transwell™ (top) and in a six-well plate (bystander; bottom) the day before the experiment. The next day, cells on the Transwell™ filters were either infected or mock infected using the procedure described above. After adsorption, medium was added, and cells were incubated 1 h at 37°C to allow internalization of the viral particles. In parallel, the medium in the six-well plate was replaced by the same medium containing 1% rabbit neutralizing antireovirus antiserum (a generous gift from Dr Earl G. Brown, University of Ottawa). After 1 h of internalization, the medium on the Transwell™ was replaced by fresh medium, and then the Transwell™ was laid atop of the second layer of cells for 15 h.

### siRNA transfection

L929 cells were plated in a 12-well plate at 75 000 cells/well and transfected on the following morning using 50 pmol of siRNA and 3.75 μl of RNAiMAX (ThermoFisher) as per the manufacturer's protocol. Ambion Silencer® Select (catalog number 4390771) siRNA were used against RIG-I (#1; ID: s106374 and #2; ID: s106375); EFTUD2 (ID: s74089); PRPF8 (ID: s101224); and SNRNP200 (ID: s115821). Cells were incubated for 56 h before being infected or mock-infected as previously described and further incubated for 16 h before harvesting RNA or proteins.

### Production of reassortant MRV by reverse genetics

The plasmids corresponding to the ten genes of MRV serotype 3 Dearing, T3D^K^, under the transcriptional control of the T7 promoter were originally obtained from the laboratory of Dr Terence Dermody (UPMC Children's Hospital of Pittsburgh, Pittsburgh, PA, USA) (50). To generate point mutations in the *M1* gene segment, standard QuikChange site-directed mutagenesis was performed. All constructions were validated using Sanger sequencing. Sequences of all primers used in mutagenesis are available upon request. Plasmids were then used to recover infectious virus by the improved reverse genetics approach using transfection in BHK cells expressing the T7 RNA polymerase with some modifications (51–53). Briefly, the 10 plasmids (100 ng of each) were simultaneously introduced alongside plasmids encoding the cytoplasmic Vaccinia Virus capping proteins D1R and D12L into semi-confluent 35 mm-diameter petri dish of BHK21-T7 cells using Fugene 6 (Roche). pCAG-D1R and pCAG-D12L were a gift from Takeshi Kobayashi (Addgene plasmid #89160; http://n2t.net/addgene:89160; RRID:Addgene_89160 and Addgene plasmid #89161; http://n2t.net/addgene:89161; RRID:Addgene_89161, respectively). Upon confluency (3-4 days), the medium was recovered, cells trypsinized and plated in a P100 dish with the medium recovered previously and completed with complete medium containing 5% heat-inactivated fetal bovine serum. Upon confluency (3–4 days), cells and their medium were subjected to three freeze-thaw cycles (−80°C/37°C) and used as starting virus stocks. Reassortant viruses were first propagated in Vero cells in the presence of chymotrypsin, as previously described (52). Upon sufficient cell lysis (2–3 days), cells and their medium were again subjected to three freeze-thaw cycles (−80°C/37°C) and used to further propagate the viruses in L929 cells.

### Molecular cloning

The genomic sequence encoding the μ2 protein (T3D^s^ strain) was amplified from the reverse genetics plasmid and cloned using HindIII and XhoI into the pEGFPN1 and pEGFPC1 plasmid. For pEGFPN1, the stop codon was removed to ensure the GFP was translated together with μ2; for pEGFPC1, two nucleotides (CG) were added before μ2 start codon to conserve the open reading frame with GFP. The P208S mutant was realized from these plasmids using the same QuikChange primers as described for the reverse genetics’ mutants. Mutants unable to accumulate in the nucleus were generated using KLD mutagenesis (New England Biolabs). To clone AS minigenes, L929 genomic DNA was harvested using DNeasy Blood & Tissue kit (Qiagen). The amplicons were amplified by PCR and cloned using KpnI and Not1 (*ALKBH1*, *SERBP1*) or by Gibson assembly (NEB) for the remaining by opening the plasmid with the same enzymes. All constructions were validated using Sanger sequencing; sequences are available upon demand.

### RNA extraction

Total RNA samples were extracted with Qiazol^®^ as recommended by the manufacturer (Qiagen).

### Reverse transcription

Reverse transcription was performed on 2.2 μg total RNA with Transcriptor reverse transcriptase, random hexamers, dNTPs (Roche Diagnostics) and 10 units of RNAse OUT (Invitrogen) following the manufacturer's protocol in a total volume of 20 μl.

### qPCR

All forward and reverse primers were individually resuspended to 20–100 μM stock solution in Tris-EDTA buffer (IDT) and diluted as a primer pair to 1 μM in RNase DNase-free water (IDT). The complete list of primers used in this study is available in Supplementary Table S1. Quantitative PCR (qPCR) reactions were performed in 10 μl in 96-well plates on a CFX-96 thermocycler (BioRad) with 5 μl of 2× iTaq Universal SYBR Green Supermix (BioRad), 10 ng (3 μl) cDNA, and 200 nM final (2 μl) primer pair solutions. The following cycling conditions were used: 3 min at 95°C; 50 cycles: 15 s at 95°C, 30 s at 60°C, 30 s at 72°C. Relative expression levels were calculated using the qBASE framework using PSMC4, PUM1 and TXNL4B as housekeeping genes. For all PCR run, control reactions performed in the absence of template were performed for each primer pair, and these were consistently negative. All qPCR data were generated following the MIQE guidelines (54).

### Alternative splicing PCR (AS-PCR)

PCR primer sequences were designed at the Université de Sherbrooke Rnomics Platform using a custom software designed to optimize standard primer design criterias, and to certify target specificity using embedded NCBI Blast software (https://blast.ncbi.nlm.nih.gov/). The primers were placed on exons flanking the alternative region to amplify both isoforms in the same PCR reaction. All forward and reverse primers were individually resuspended to 20–100 μM stock solution in Tris-EDTA buffer (IDT) and diluted as a primer pair to 1.2 μM in RNase DNase-free water (IDT). End-point PCR reactions were done on 10 ng cDNA in 10 μl final volume containing 0.2 mmol/l each dNTP, 1.5 mmol/l MgCl_2_, 0.6 μmol/l each primer, and 0.2 units of Platinum Taq DNA polymerase (Invitrogen). An initial incubation of 2 min at 95°C was followed by 35 cycles at 94°C 30 s, 55°C 30 s and 72°C 60 s. The amplification was completed by a 2 min incubation at 72°C. PCR reactions were carried on thermocyclers GeneAmp PCR System 9700 (ABI), and the amplified products were analyzed by automated chip-based microcapillary electrophoresis on LabChip GX Touch HT Nucleic Acid Analyzer (PerkinElmer). Amplicon sizing and relative quantitation were performed by the manufacturer's software, before being uploaded to the LIMS database. The percent spliced-in (PSI) metric was used to quantitate the level of inclusion in these alternative splicing events. It represents the percent of the long form over total abundance for both the long and short forms. The formula is as follows:

$$\begin{equation*}PSI\; = \;\frac{{Long\;form}}{{Long\;form + Short\;form}}\end{equation*}$$

For the minigene reporters, the reverse primer was substituted for the BGH primer from the pcDNA3.1+ plasmid alongside the usual forward primer, only allowing the monitoring of the RNA derived from the plasmid and not from the endogenous gene. The only exception was *SERBP1* for which the forward primer was substituted for another primer in the second exon to generate shorter amplicons to correctly separate the two forms in capillary electrophoresis.

### Plasmid transfection

293T cells were plated in a 12-well plate at 400 000 cells/well (24 h) or 300 000 cells/well (48 h) and transfected on the following morning using Lipofectamine2000 (ThermoFisher) and 1.5 μg of plasmid DNA. Only 75 ng (20× less) of the control empty plasmid encoding only GFP (pEGFPN1) was transfected to normalize the expression of the GFP alone to GFP-μ2 proteins; empty pcDNA3.1+ was used to fill the remaining 1.5 μg of plasmid DNA. AS minigenes were co-transfected altogether with the plasmid of interest at 10 ng per AS minigene.

### Western blot

The linearity of all antibodies used in this study was first experimentally determined to allow for an adequate quantification in the linear range of both samples analyzed and the antibody used. Cells were rinsed with PBS and lysed in RIPA Buffer (1% Triton X-100, 1% sodium deoxycholate, 0.1% SDS, 1 mM EDTA, 50 mM Tris–HCl pH 7.5 and complete protease inhibitor (ROCHE)) on ice. Upon transfer in a microtube, DNA was fragmented using ultrasound on ice at 13% amplitude for 5 s, two to four times. Debris were then pelleted at 13 000 RPM, 4°C, 10 min. Lysates were dosed for total protein in triplicate using standard Bradford assay (Thermo Scientific Coomassie Protein Assay). The appropriate quantity of protein was diluted with water and Laemmli 4x buffer. Samples were heated 5 min at 95°C. Samples were loaded on 10% or 6% SDS-polyacrylamide gels and electrophoresis was carried out at 150 V. The Bluelf protein ladder was used a molecular weight marker (FroggaBio). Gels were transferred onto a polyvinylidene difluoride (PVDF) membrane at 4°C, 75 min, 100 V. Membranes were blocked in 5% non-fat milk in TBS-T (10 mM Tris–HCl pH 8.0, 220 mM NaCl, 0.1% Tween 20), 1 h at room temperature. Membranes were incubated overnight with the appropriate antibody in 2.5% milk/PBS. The commercial antibodies used in this study are the following: Actin (Sigma, A5441, 1:10 000), CAMK2D (Abcam, ab181052, 1:2000), CAMK2G (Abcam, ab201966, 1:500), EFTUD2 (Abcam, ab188327, 1:2000), GAPDH (Sigma, G9545, 1:12,000), GFP (Santa Cruz Biotechnology, sc-9996, 1:8000), RIG-I (Cell Signaling Technology, #3743, 1:1000), PRPF6 (Abcam, ab99292, 1:2000), PRPF8 (Abcam, ab79237, 1:1000), SNRNP200 (Abcam, ab241589, 1:1000), U2AF35 (Abcam, ab172614, 1:2000), Vinculin (Santa Cruz Biotechnology, sc-73614, 1:1000). The anti-μ2 T1L is a rabbit antiserum (55) and was diluted 1:1000; the σ3 antibody is the supernatant from a mouse hybridoma cells expressing the monoclonal antibody 4F2 (56) and was diluted 1:100. Membranes were washed 3× in TBS-T and incubated with a horse anti mouse-HRP secondary antibody 1:5000 (Cell Signaling Technologies, 7076) or goat anti rabbit-HRP secondary antibody 1:10 000 (Abcam, ab205718) during 1 h at room temperature. Membranes were washed again 3 times with TBS-T and once with PBS. Bound antibodies were revealed using Clarity ECL western blotting substrates (BIO RAD) except for the lowly expressed CAMK2G that required the Clarity Max ECL western blotting substrates (BIO RAD) and scanned on an ImageQuant LAS4000 (GE Healthcare Life Science). For quantification, HRP was inactivated using 30% H_2_O_2_ for 30 min, followed by 2x PBS washing, and membranes were blocked again and probed for the relevant loading control. All western blots were performed three times, and a representative result is presented in the article. All uncropped western blots are available in the Supplementary Figure S32.

### Immunoprecipitation (IP)

293T cells were seeded at 6 × 10^6^ cells/100 mm dish and transfected on the following morning with 10 μg of plasmid DNA and Lipofectamine2000 (ThermoFisher) as per the manufacturer's protocol. After 24 h incubation, cells were washed with PBS and 1 mL lysis buffer (1% Triton X-100; 150 mM NaCl; 20 mM Tris–HCl, pH 7.5; 0.1 mg/ml PMSF) and incubated 5 min on ice. Petri dishes were scraped, and protein lysis was completed by a couple up and down. Lysates were sonicated on ice at 25% amplitude, 5 s for four times. Debris were then pelleted at 13 000 RPM, 4°C for 10 min. Lysates were dosed for total protein in triplicate using standard Bradford assay (Thermo Scientific Coomassie Protein Assay). One milligram of lysate was DNAse and RNAse treated using 5 μg of DNAse I (Sigma) and 5 μg of RNAse A (Bio Basic), 10 min at room temperature. GFP-trap beads (Chromotek) were washed twice in lysis buffer; 20 μl were added per IP and IP reactions were completed at 1 mL with lysis buffer. IP was performed for 4 h at 4°C on a tube rotator. Tubes were then spinned at 2000 RPM, the supernatant was removed, and beads were washed three times with lysis buffer and twice with PBS. Immunoprecipitates were subjected to mass spectrometry preparation, or resuspended in 1× Laemmli buffer, boiled, and submitted to western blotting.

### LC–MS/MS preparation and analysis

All solutions for this section were prepared in MS-grade water. Beads were washed five times with 20 mM ammonium bicarbonate in LoBind eppendorfs. Proteins were then reduced with 10 mM DTT in 20 mM ammonium bicarbonate for 30 min with shaking at 1250 RPM, 60°C. Proteins were then alkylated by adding an equal volume of 15 mM chloroacetamide in 20 mM ammonium bicarbonate in the dark with shaking at 1250 RPM for 1 h. Proteins were digested with 1 μg of trypsin at 37°C overnight with shaking at 1250 RPM. Trypsin digestion was stopped by acidifying to a final concentration of 1% formic acid (FA) and supernatant was harvested upon centrifugation at 2000 *g* for 3 min. Beads were washed again in 100 μl of a 60% acetonitrile/1% formic acid solution for 5 min with shaking at 1250 RPM at room temperature and centrifuged at 2000 *g* for 3 min. This second supernatant was pooled with the first and dried in a speed vac. The peptides were resuspended in 30 μl of 0.1% trifluoroacetic acid (TFA) to desalt on ZipTip. Desalted peptides were dried again with a speed vac and resuspended in 30 μl of 1% formic acid. Peptides were quantified using a nanodrop at 205 nm and 250 ng of each sample were injected into a nanoElute HPLC (Bruker Daltonics); loaded onto a trap column with a constant flow of 4 μl/min (Acclaim PepMap100 C18 column, 0.3 mm id × 5 mm, Dionex Corporation); and eluted onto an analytical C18 Column (1.9 μm beads size, 75 μm × 25 cm, PepSep). A 2 h gradient of acetonitrile (5–37%) in 0.1% FA at 500 nl/min was used to elute peptides and inject them into a TimsTOF Pro ion mobility mass spectrometer equipped with a CaptiveSpray nano electrospray source (Bruker Daltonics). Data was acquired using data-dependent auto-MS/MS with a 100-1700 *m*/*z* mass range, with PASEF enabled with a number of PASEF scans set at 10 (1.27 s duty cycle) and a dynamic exclusion of 0.4 min, m/z dependent isolation window and collision energy of 42.0 eV. The target intensity was set to 20 000, with an intensity threshold of 2500.

### Protein identification by MaxQuant

The raw files were analyzed using MaxQuant (version 1.6.17.0, (57)) and the Uniprot human proteome database (21/03/2020, 75 776 entries) supplemented with both μ2 and μ2-P208S sequences. The settings used for the MaxQuant analysis (with TIMS-DDA type in group-specific parameters) were: 2 miscleavages allowed; fixed modification: carbamidomethylation on cysteine; enzyme was trypsin (K/R not before P); variable modifications included were methionine oxidation, and protein N-terminal acetylation. The mass tolerance were 10 ppm (precursor ions) and 20 ppm (fragment ions). FDR (PSM and protein) and site decoy fraction were set to 0.05; minimum peptide count was set to 1. Label-Free-Quantification (LFQ) was also allowed with minimal ratio count of 2. The ‘Second peptides’ and ‘Match between runs’ options were both allowed. Following the analysis, proteins positive for at least either one of the ‘Reverse’, ‘Only.identified.by.site’ or ‘Potential.contaminant’ categories were eliminated, as well as proteins identified from a single peptide.

### Indirect immunofluorescence in COS-7 cells

COS-7 cells were seeded at 1 × 10^4^ cells/well in 24-well plates on glass coverslips and transfected on the next morning. After a 24 h incubation period, cells were washed with PBS and fixed 20 min using 4% paraformaldehyde and 4% sucrose in PBS at room temperature. Cells were then permeabilized with 0.15% triton X-100 in PBS for 5 min at room temperature and blocked in 10% normal goat serum (Wisent) for 20 min. Anti-GFP antibody (1:1000) was incubated 4 h at room temperature to increase the GFP signal. Cells were washed three times in 10% normal goat serum for 5 min and incubated 1 h in the dark with AlexaFluor 488-labelled goat anti-mouse secondary antibody (Invitrogen, 1:1000). Cells were washed three time in PBS, and nucleus staining was performed using 1 μg/ml Hoechst for 15 min at room temperature. Coverslips were mounted on slides with SlowFade Diamond mounting medium (Life Technologies), then confocal microscopy imaging was done using a confocal Zeiss LSM 880 2 photons microscope.

### Data analysis and statistical analyses

Image quantitation was done using the ImageJ software (Fiji). All statistical analyses were conducted with the GraphPad software. All results presented in this article are mean ± standard deviation.

## RESULTS

### The interferon response is not necessary nor sufficient to trigger AS changes during MRV infection

**Figure 1.**
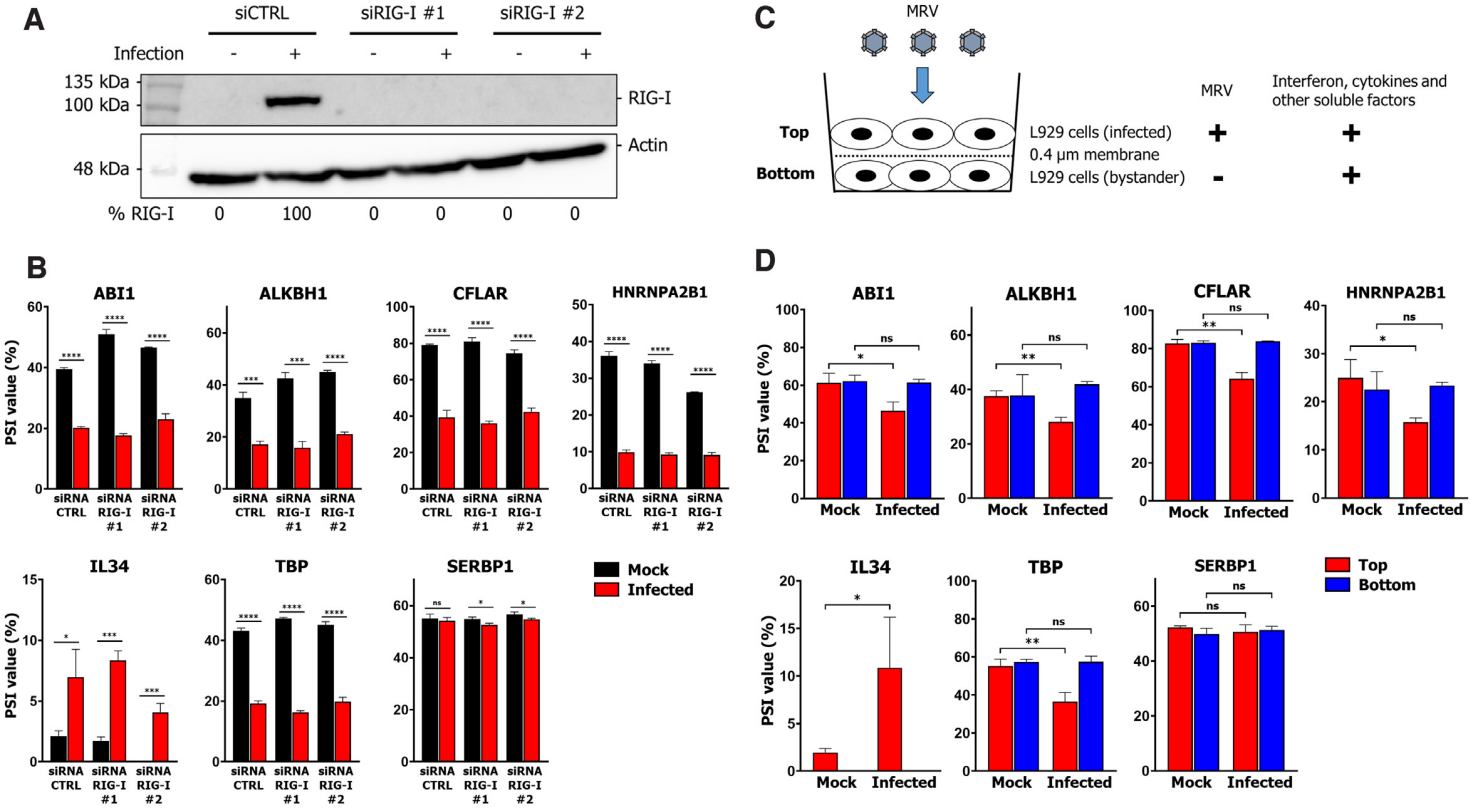
The interferon response is not necessary nor sufficient to trigger AS changes during MRV infection. (**A**) Validation by western blot of RIG-I knockdown in mock- and MRV- (T3D^S^) L929 infected cells. The membrane was H_2_O_2_-inactived and probed against actin as a loading control. (**B**) Percent spliced in (PSI) values for numerous ASE modulated by MRV in siCTRL and siRIG-I mock (black) and infected (red) L929 cells. *SERBP1* was included as a negative control ASE. n = 3, biological replicates, unpaired two-tailed Student's t-test (ns, *P* > 0.05; *P ≤ 0.05; **P ≤ 0.01; ***P ≤ 0.001; ****P ≤ 0.0001) comparing mock and infected cells for each siRNA condition. (**C**) Overview of the bystander experiment. (**D**) PSI values of the same ASE as B in the membrane (top, red) or the bystander (bottom, blue) cells when the top layer was infected or mock-infected with MRV. *SERBP1* was included as a negative control ASE. *n* = 3, biological replicates, unpaired two-tailed Student's *t*-test (ns, *P* > 0.05; *P ≤ 0.05; **P ≤ 0.01) comparing mock and infected cells on the membrane (top, red) or bystander (bottom, blue).

Upon infection of L929 murine cells with MRV, we and others have previously demonstrated that drastic changes in cellular AS occur 16 h post-infection (PI) (44,45). These changes could either be (i) induced directly by the presence of the virus; (i) triggered by the host cell as a defense mechanism or (iii) linked to the antiviral state mediated through secreted factors, such as IFN, cytokines or other ISG. We initially wanted to address if IFN induction is required for MRV modulation of cellular AS. To do so, we targeted RIG-I, one of the principal cytoplasmic sensors of viral dsRNA and ssRNA allowing IFN production during MRV infection (58,59). siRNA-mediated knockdown (KD) of RIG-I using two different siRNA completely abrogated its expression in MRV-infected L929 at 16 h PI (Figure 1A). Further validation by measuring mRNA levels of IFN-β and two ISG (*DDX60* and *MX1*) confirmed an 80-90% reduction in IFN-β mRNA levels and a 60% (siRIG-I #1) to 80–90% (siRIG-I #2) reduction in ISG induction (Supplementary Figure S1). Next, we harvested RNA from both mock and infected cells at 16 h PI in either siCTRL or siRIG-I conditions, and submitted them to alternative splicing PCR (AS-PCR) coupled to capillary electrophoresis to quantitate potential changes in AS (60–64). Alternative splicing events (ASE) were selected from the 240 ASE previously shown to be modulated during MRV infection and validated using AS-PCR (44). All pertinent information regarding the primers and the amplicon quantitated are available in Supplementary Table S2, and design maps for every ASE analyzed are depicted in Supplementary Figure S2. The percent spliced-in (PSI) metric was used to quantitate the level of intron inclusion in these ASE; it represents the percent of the long form over total abundance (both long and short forms). In the control siRNA transfection, MRV drastically changed the AS of the ASE we monitored, as we have previously demonstrated (44) (Figure 1B). We also analyzed an ASE in the *SERBP1* gene, which is not modulated by MRV, as a negative control to confirm that MRV’s impact on cellular AS is targeted to specific transcripts, as we previously described (44). Upon RIG-I KD, the capacity of MRV to alter the splicing of these events was not affected, indicating that the interferon response is not necessary for MRV modulation of these ASE (Figure 1B). However, a very limited number of ASE, such as in *CKDN2AIP* and *EIF4A2*, were affected by the status of the IFN response. For instance, in the case of *CKDN2AIP*, RIG-I KD limits MRV ability to alter AS. For *EIF4A2*, the AS in uninfected cells is modulated by the RIG-I KD (Supplementary figure S3). As controls, we also monitored viral *M1* and S1 RNA levels (Supplementary Figure S4) and viral protein levels (Supplementary Figure S5) to ensure that knocking RIG-I was indeed increasing viral RNA and protein levels.

We next wondered if soluble factors secreted upon infection were sufficient to trigger the observed AS changes. To do so, we designed a bystander experiment aimed at answering this question (Figure 1C). First, L929 cells were plated on a 0.4 μM semi-permeable membrane and infected by MRV, before being laid atop a second layer of uninfected cells in 1% anti-MRV antibody. In such a system, the first layer of cells is infected, but not the second layer, and no virus is present in these bystander cells nor the mock experiment as determined by measuring the levels of *M1* and *S1* viral RNA by qPCR (Supplementary Figure S6). However, bystander cells are stimulated by secreted factors from infected cells, as demonstrated by the levels of induction of two ISG at the RNA level (Supplementary Figure S6). Upon analysis of the same ASE as Figure 1B, the bystander experiment in the uninfected control condition showed no change in AS, whether cells were on the membrane (top) or bystander (bottom) to those cells (Figure 1D). In the infected condition, a change in AS can be observed in cells cultured on the membrane (top, red); however, no impact on AS can be observed in bystander cells (bottom, blue) for all ASE tested (Figure 1D, Supplementary Figure S7). The *SERBP1* negative control ASE was constant in all tested conditions. These results indicate that secreted factors are not sufficient to mediate changes in these ASE in bystander cells. To further rule out any possibility that IFN signaling might affect AS, we directly treated L929 cells with 10, 100, or 1000 U/ml of recombinant mouse IFN-β and demonstrated that all concentrations of IFN-β do no impact the AS of selected ASE (Supplementary Figure S8). Taken together, these data suggest the IFN response is neither necessary (RIG KD) nor sufficient (bystander experiment) for MRV’s impact on these cellular ASE.

### The modulation of AS happens in a time-dependent manner during MRV infection

**Figure 2.**
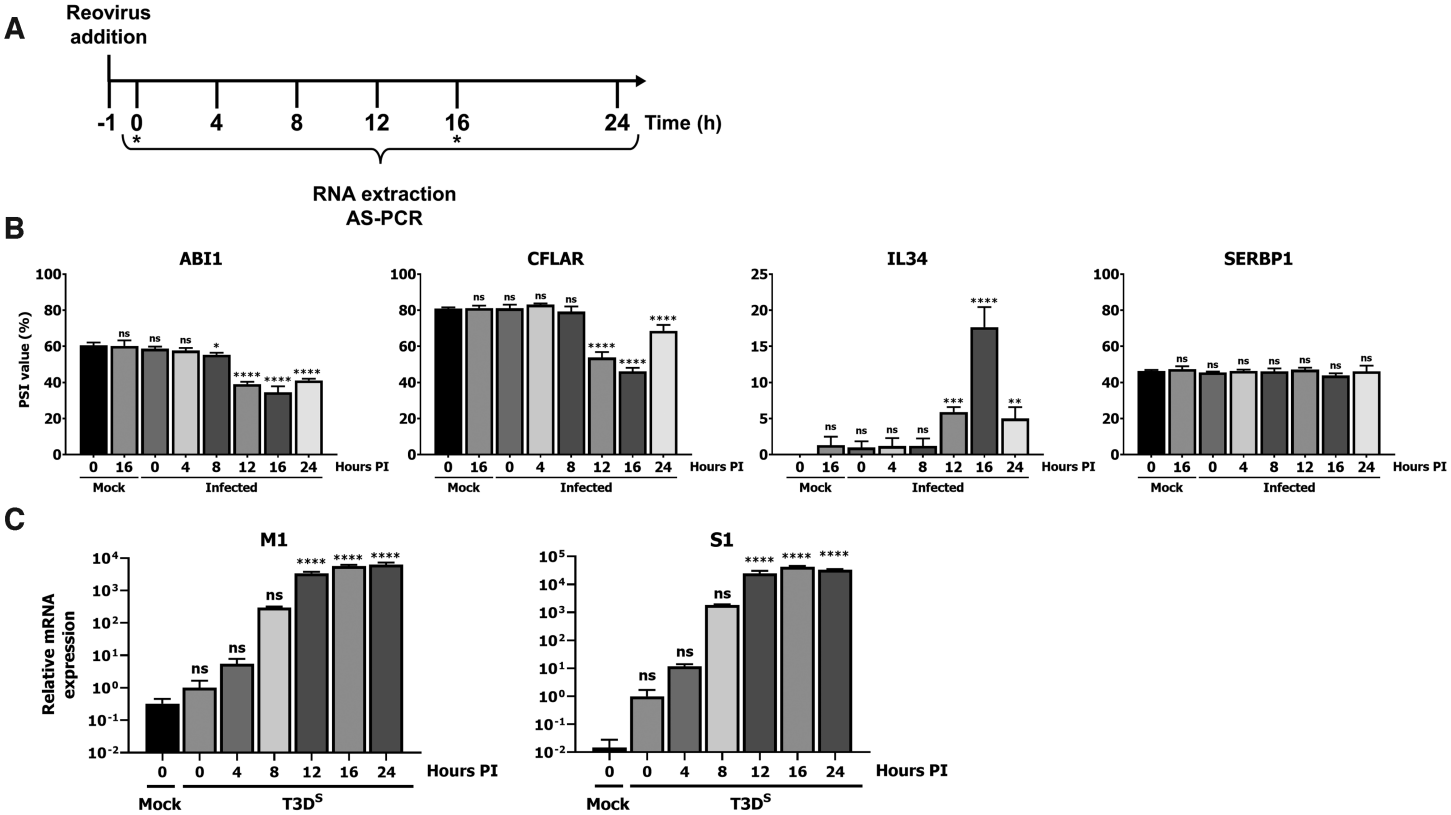
The modulation of AS occurs in a time-dependent manner during MRV infection. (**A**) Overview of the time-course experiment. Mock cells were harvested at the 0 h and 16 h time point (denoted by an asterisk). (**B**) Splicing profiles (PSI) of the *ABI1*, *CFLAR*, *IL34*, and negative control *SERBP1* ASE throughout infection. PCR amplicons were resolved using capillary electrophoresis and quantified using relative fluorescence. *n* = 3, biological replicates, one-way ANOVA with Dunnett's multiple comparisons test against the 0 h mock condition (ns, *P* > 0.05; *P ≤ 0.05; **P ≤ 0.01; ***P ≤ 0.001; ****P ≤ 0.0001). (**C**) Relative levels of *M1* and *S1* RNA as determined by qPCR throughout the time-course experiment and up to 24 h. The first time point upon adsorption (0h post-infection, T3D^S^) was used to normalize the RNA level to the input RNA that was present to infect cells. PSMC4, PUM1 and TXNL4B were used as housekeeping genes for normalization. *n* = 3, biological replicates, one-way ANOVA with Dunnett's multiple comparisons test against the 0 h mock condition (ns, *P* > 0.05; ****P ≤ 0.0001).

Since the preceding results suggested that MRV presence is necessary to induce changes in AS, we next assessed the kinetics of these changes observed during infection. Changes in AS early in infection could point towards early replication steps, such as internalization or uncoating of the viral particle, to be involved. On the other hand, changes appearing later during infection would suggest that the translation of viral RNA and production of viral proteins are required, thereby suggesting the involvement of newly produced viral proteins. A time-course experiment was performed where RNA was harvested directly after adsorption (0 h) and up to 24 h PI (Figure 2A). The splicing profile of ASE modulated during infection was then assessed using AS-PCR. The time-course experiment revealed that upon adsorption and early during infection (4 h and 8 h), no significant modulation in the AS profiles of *ABI1*, *CFLAR* and *IL34* could be detected (Figure 2B). However, at 12 h, the splicing profile started to shift and peaked at 16 h post-infection for the three ASE analyzed. These splicing changes were not further modulated at 24 h, and some PSI values were even returning towards the basal level in some case (*CFLAR*, *IL34*). Monitoring the AS profile of the negative control ASE (*SERBP1*) showed no change throughout the infection, hence showing that the modulation of AS is specific and timely regulated (Figure 2B). Additional ASE were analyzed and showed similar profiles; however, some rare examples, such as the one in *EIF4A2*, were modulated using a different kinetic (Supplementary Figure S9). We monitored viral replication by following levels of *M1* and *S1* RNA by qPCR during the same time course and confirmed that MRV replication peaks at 16 h post-infection (Figure 2C), as previously described under similar conditions (65). Since these AS changes happen in a coordinated fashion with the peak of viral replication, the simultaneous timing of these two events suggests that the production of some viral protein(s), reaching a critical peak at this time point (65), is involved in this modulation.

### The μ2 protein is the main determinant of the modulation of cellular AS during MRV infection

**Figure 3.**
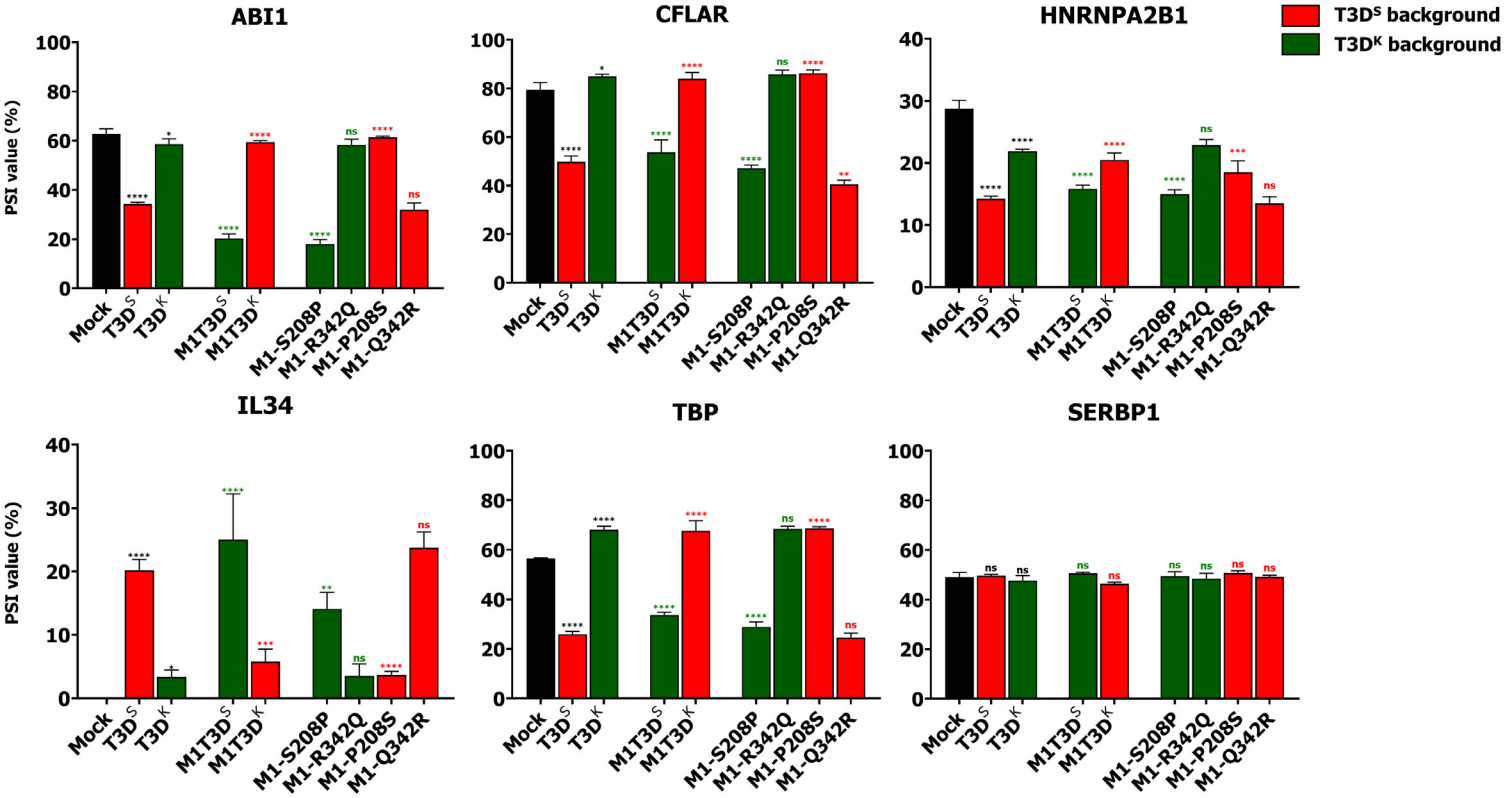
Strain-dependent modulation of AS segregates with the *M1* gene segment and is dictated by a polymorphism at position 208 in μ2. Splicing profile (PSI) of the ASE in the *ABI1*, *CFLAR*, *HNRNPA2B1*, *IL34* and *TBP* gene upon infection with (i), T3D^S^ or T3D^K^ MRV; (ii), T3D^S^ harboring the *M1* gene segment from T3D^K^ (M1T3D^K^[T3D^S^]) or T3D^K^ harboring the *M1* gene segment from T3D^S^ (M1T3D^S^[T3D^K^]) and (iii), single amino acid mutant in μ2 S208P or R342Q in T3D^K^ and P208S or Q342R in T3D^S^. PCR amplicons were resolved using capillary electrophoresis and quantified using relative fluorescence. The negative control ASE in *SERBP1* was also analyzed. *n* = 3, biological replicates, one-way ANOVA with Dunnett's multiple comparisons test against the mock condition for T3D^S^/T3D^K^ (in black); two-way ANOVA with Šídák's multiple comparisons test against the parental virus (T3D^S^ in red and T3D^K^ in green) for the reassortant and mutant viruses (ns, *P* > 0.05; *P ≤ 0.05; **P ≤ 0.01; ***P ≤ 0.001; ****P ≤ 0.0001).

In an effort to identify which protein(s) could be involved, we decided to assess the ability of another MRV T3D strain to modulate AS. The rationale is that if a strain-dependent phenotype is present, then mapping that phenotype to one of the ten gene segments, each encoding one or two proteins, should be possible. To do so, we compared our MRV laboratory stock, T3D^S^, with the one from the original reverse genetics system, T3D^K^ (50). There are a dozen single amino acid polymorphisms between these two virus strains, leading to drastic differences in some phenotypes such as the induction of interferon and the morphology of the viral factories (5,48,66). L929 cells were infected with either T3D^S^, T3D^K^ or mock-infected, and RNA was harvested 16 h post-infection. Following AS-PCR, T3D^K^ appeared to be a less-potent modulator of AS than T3D^S^ for the various ASE analyzed (Figure 3). For example, the AS of *ABI1*, *CFLAR* and *IL34* is efficiently modulated by T3D^S^, but not by T3D^K^. Both T3D^S^ and T3D^K^ modulate the splicing of *TBP*; however, T3D^K^ induces the accumulation of the long form instead of the short form of the ASE as seen with T3D^S^. Again, additional ASE analyzed are shown in Supplementary Figure S10 and further confirmed the different impact of T3D^S^ and T3D^K^ on cellular AS. The negative control ASE *SERPB1* shows that the two viruses are not broadly impacting AS but rather modulating only specific ASE.

Since the two laboratory strains present striking differences in their ability to modulate the splicing of these ASE, recombinant viruses were produced to map this strain-specific phenotype to one or more of their gene segments. It has been previously observed that the μ2 protein from another MRV strain (T1L) interacts with the splicing factor SRSF2 (45). Involvement of the *M1* gene segment, encoding for the μ2 protein, was thus first questioned by swapping the *M1* gene segment from T3D^K^ into the genetic background of T3D^S^ (M1T3D^K^[T3D^S^]), or reciprocally (M1T3D^S^[T3D^K^]). L929 cells were then infected using these two viruses and the splicing profiles characterized by AS-PCR, as before. Upon infection with either virus, their splicing profiles were mimicking the one from which the *M1* gene segment comes from, i.e., the splicing of M1T3D^K^[T3D^S^] was the same as the WT T3D^K^, and the same observation can be made for M1T3D^S^[T3D^K^] and T3D^S^ (Figure 3). This experiment confirmed that the strain-dependent phenotype in the ability to modulate these ASE mostly segregates with the *M1* gene segment, and thus suggests the involvement of the μ2 protein. Again, additional ASE analyzed are shown in Supplementary Figure S10 and confirmed that ability to modulate the AS of these genes is linked to the *M1* gene segment. However, it is also possible to observe some effects of the genetic background. For example, in the M1T3D^S^[T3D^K^] infection, the modulation of *ABI1* and *TBP* ASE is respectively increased and decreased as compared to WT T3D^S^. Other genetic determinants might thus be involved, either helping or restricting μ2 impact on AS. The same negative control as previously used, *SERPB1*, was also analyzed. Once again, this ASE was not modulated in all the viruses tested. To rule out any possibility that slower replication kinetics might be explaining these results, we monitored the levels of *M1* and *S1* RNA at 16 h PI as a general indicator of viral replication. All viruses tested only show slight significant differences in their levels of *M1* and *S1* RNA (Supplementary Figure S11). This indicates that these different virus strains replicate efficiently and do not exhibit gross defects in replication. Moreover, even the lower replicating viruses, such as M1T3D^S^[T3D^K^], induced changes in AS, indicating that small differences in replication levels cannot explain the differences in the ability to induce changes in cellular AS. Monitoring viral σ3 protein levels also confirmed similar replication for all viruses tested (Supplementary Figure S12). Altogether, these results clearly established that the strain-dependent phenotype in the modulation of these ASE is caused by the *M1* gene segment, and that the μ2 protein from T3D^S^ and T3D^K^ possesses drastically different capacities to alter AS during infection.

Interestingly, there are only two amino acid differences in μ2 between T3D^S^ and T3D^K^. In the case of T3D^S^, position 208 is a proline, and position 342 is a glutamine; in the case of T3D^K^, these positions are occupied by a serine and an arginine, respectively (48). The position 208 has already been involved in numerous strain-dependent phenotype attributed to μ2, such as the blockade of the IFN response and morphology of the viral factories (5,67,68). No strain-dependent phenotype has been linked to the position 342. We thus assessed which polymorphism (i.e. position 208 or 342) is responsible for this differential ability to modulate the cellular AS of these ASE by substituting the amino acid at these positions with the one from the other strain. Site-directed mutagenesis was conducted on the reverse genetic plasmid encoding the T3D^K^*M1* gene segment to introduce individually S208P or R342Q mutation in the T3D^K^ μ2 protein. This process was also done using the T3D^S^ μ2-encoding plasmid to introduce reciprocal mutations, P208S or Q342R. T3D^S^ or T3D^K^ viruses harboring these single amino acid mutations in the μ2 protein were rescued by reverse genetics, L929 cells were infected as before, and their ability to modulate the AS of target ASE evaluated using AS-PCR. Mutating the proline to serine at position 208 in μ2 from T3D^S^ abrogates its ability to strongly modulate these ASE; reciprocally mutating the serine to proline at this position in T3D^K^ μ2 rescue this phenotype (Figure 3). Mutating the arginine to glutamine or vice versa at position 342 has no effect on the modulation of AS for these genes. Once again, some influence from the genetic background can be observed for certain ASE. For example, the M1-S208P T3D^K^ virus induces a bigger change in the splicing of the *ABI* ASE than WT T3D^S^, indicating that additional factors in the background can influence μ2’s impact on AS. Additional ASE analyzed are presented in Supplementary Figure S10 and support both the involvement of the amino acid at position 208 in μ2 and the importance of MRV genetic background in the modulation of cellular AS. The AS of the negative control *SERBP1* was the same in all viruses tested. Once again, viral RNA levels (Supplementary Figure S11) and protein levels (Supplementary Figure S12) confirmed only minor differences in replication levels that cannot explain the impact on cellular AS. Taken together, these results show that the amino acid at position 208 in μ2 is critical for its impact on AS, and is mainly responsible for the strain-dependent phenotype previously described.

### μ2 directly impacts cellular AS of reporter minigenes in ectopic expression through both nuclear-dependent and nuclear-independent mechanisms

Our results so far demonstrate that MRV μ2 protein is the main determinant of MRV modulation of AS during infection, at least for the ASE we analyzed. However, we cannot conclude solely from these results that the μ2 protein is directly able to trigger these changes during infection, as all other viral proteins are expressed and might be indirectly necessary for the μ2-dependent alterations of AS. We thus needed to assess the ability of μ2 by itself to modulate cellular AS in the absence of other viral proteins. However, ectopic expression of μ2 was previously described to be challenging, especially in L929 cells classically used for MRV infection studies (55,69). To circumvent this problem, HEK293 and 293T cells have been shown to be suitable for the transient expression of the μ2 protein alone (9,45,68,70). We found that SV40 large T antigen harboring cells such as 293T and COS-7 were the most efficient for μ2 expression harboring either a N- or C-terminal GFP moiety (Supplementary Figure S13).

**Figure 4.**
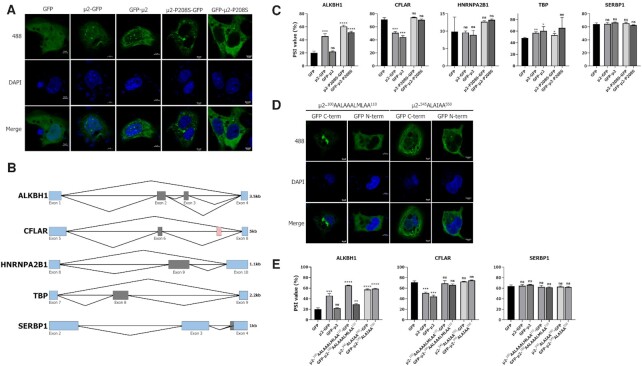
μ2 directly impacts the AS of minigene reporters in ectopic expression through both nuclear-dependent and nuclear-independent mechanisms. (**A**) Cellular localization of GFP, N-term or C-term GFP-μ2, and N-term or C-term GFP-μ2 harboring the single amino acid polymorphism P208S in COS-7 cells. The white scale bar represents 5 μm. (**B**) Schematic representation of the selected ASE cloned into the pcDNA3.1+ plasmid to act a AS reporters. Black lines represent introns and rectangles denote exons; in blue are the constitutive ones and in gray are the alternatively spliced ones. All selected ASE modulated by MRV are single exon cassette, except for the ASE in *ALKBH1* where there are two consecutive cassette exons. The negative control *SERBP1* is an alternative 3′ splice site (+18 nucleotides) that was previously monitored; although the exon 3 is annotated as a cassette exon, we have never observed the skipping of this exon in L929 cells. Introns and exons are to scale (for each ASE separately) and the length of the cloned fragment is denoted on the right. The red hatched exon in *CFLAR* denotes the 7^th^ exon, which an alternative transcription initiation site and is only included when transcription begins at this exon. (**C**) Splicing profiles (PSI) of the *ALKBH1*, *CFLAR*, *HNRNPA2B1, TBP*, and the negative control *SERBP1* AS minigenes reporters upon expression of the different μ2 constructs. n = 3, biological replicates, unpaired two-tailed Student's *t*-test (ns, *P* > 0.05; *P ≤ 0.05; **P ≤ 0.01; ***P ≤ 0.001; ****P ≤ 0.0001) against the GFP alone condition. (**D**) Cellular localization of N-term or C-term GFP-μ2 mutants 100-AALAAALMLAA-110 and 545-ALAIAA-550 unable to accumulate in the nucleus in COS-7 cells. The white scale bar represents 5 μm. (**E**) Splicing profiles (PSI) of the *ABI1*, *CFLAR*, and the negative control *SERBP1* AS minigene reporters upon expression of the different μ2 constructs. *n* = 3, biological replicates, unpaired two-tailed Student's *t*-test (ns, *P* > 0.05; *P ≤ 0.05; **P ≤ 0.01; ***P ≤ 0.001; ****P ≤ 0.0001) against the GFP alone condition.

We first monitored the cellular localization of the μ2 protein harboring a N- or C-terminal GFP moiety, or corresponding mutants where the proline at 208 was substituted for a serine (P208S) in COS-7 cells (Figure 4A). As previously shown, the WT μ2 showed filamentous localization in the cytoplasm by binding to cellular microtubules (5), and broad nuclear staining with bright foci described previously as nuclear speckles (9,45). Moreover, the P208S mutants showed a diffuse cytoplasmic staining, as it cannot bind to microtubules (5). Surprisingly, the P208S mutant was devoid of any nuclear accumulation, both in terms of the number of cells presenting nuclear μ2 as well as when quantifying the fluorescent signal inside the cells (Supplementary Figure S14A, B). Moreover, the same results were obtained in 293T cells (Supplementary Figure S15). This raises the possibility that incorrect import into the nucleus for the P208S mutant might be responsible for its reduced impact on cellular AS. To test this hypothesis, we monitored the ability of these constructions to alter AS in ectopic expression. Since AS is not very well conserved between mouse and human (71), we derived AS minigene reporters from the murine L929 cells for *ALKBH1*, *CFLAR*, *HNRNPA2B1*, *TBP* and *SERBP1* ASE (Figure 4B). These constructs encompass the spliced region known to be modulated by MRV that we previously monitored (Figures 1B, 1D,2B, and 3) into the pcDNA3.1+ vector. All pertinent information regarding the primers and the amplicon quantitated are available in Supplementary Table S3A. These constructs allowed the monitoring of the AS of these five ASE when co-transfected in 293T cells together with the different μ2 constructs. Western blotting (Supplementary Figure S16) and epifluorescence microscopy (Supplementary Figure S17) confirmed that all the constructions tested were correctly expressed. We first assessed the ability of WT μ2 or P208S-μ2 to alter the splicing of these minigenes. The PSI in the *CFLAR* ASE was reduced upon WT μ2 expression but not when the proline was substituted for a serine, recapitulating the phenotypes observed during MRV infection (Figure 3). Surprisingly, the PSI for the *ALKBH1* was strongly increased by the WT μ2 harboring the C-term GFP or both P208S mutants (Figure 4C), as opposed to the reduction in inclusion of this ASE previously observed during infection (Figure 1B, Supplementary Figure S10). However, this event is more complex than the other ones, as two long forms are present (exons 1-2-4 and exons 1-2-3-4) that might complexify the interpretation. As a control, the AS of the 3′-SS from the *SERBP1* minigene was not altered by the expression of any of these constructions. Together, these three minigenes reveal that μ2 protein expression alone specifically alters the AS profiles of ASE targeted during MRV infection. However, we failed to observe any changes in the splicing of the *HNRNPA2B1* and *TBP* reporters; these results will be addressed below in section *U5 core components are required for MRV modulation of cellular AS and are reduced by μ2*. Finally, we also exploited two conserved ASE between mouse and human dysregulated during MRV infection, namely *ABI1*, *CDKN2AIP*, in addition to the *SERBP1* negative control which is also conserved (Supplementary Table S3B), and analyzed their splicing upon the expression of the μ2 constructs (Supplementary Figure S18). The ASE in *ABI1* and *CDKN2AIP* were both modulated efficiently by μ2-GFP and μ2-P208S-GFP, but not when the GFP moiety was fused to the N-terminus of μ2. Once again, the negative control ASE was not impacted by μ2 expression. These results suggest an involvement of the N-terminus of μ2 in the modulation of the ASE in *ABI1*, *CDKN2AIP*, and *ALKBH1*. Taken together, only the ASE in *CFLAR* (minigene) was not modulated by the P208S mutants; ASE in *ALKBH1* (minigene), *ABI* (endogenous), and *CDKN2AIP* (endogenous) were all affected in the same fashion by the mutant than the WT μ2.

These previous results support that μ2 is altering cellular AS through both nuclear-dependent and nuclear-independent mechanisms. However, substituting the P208 for a serine might affect other aspects of μ2 than the localization, and we thus wanted to specifically target the ability of μ2 to enter the nucleus. One nuclear localization sequence (NLS) in μ2, 100-RRLRKRLMLKK-110, was previously described (9). However, it was never mutated in the context of the endogenous protein, but rather studied when grafted alone to GFP moiety (9). We also identified another potential NLS, 545-RLKIPY-550, satisfying the R/H/K-X_(2–5)_P-Y rule for PY-NLS recognized by Kapβ2 (72) (Supplementary Figure S19). In all these sequences, all basic residues were substituted for alanine, and the resulting mutants tested for their ability to accumulate in the nucleus in transiently-transfected COS-7 cells. All these mutations severely impaired nuclear localization, indicating that altering either of these sequences is sufficient to restrain the μ2 protein to the cytoplasm (Figure 4D, Supplementary Figure S14A, S14B). Next, we assess the ability of these mutants to alter cellular AS of the reporter minigenes for *ALKBH1*, *CFLAR*, and *SERBP1*. All mutants were still able to alter the splicing of the *ALKBH1* minigene and unable to alter the splicing of *CFLAR* (Figure 4E). Moreover, the mutations restraining μ2’s ability to accumulate in the nucleus also had no impact on the capacity to alter the endogenous ASE in *ABI1* and *CDKN2AIP* (Supplementary Figure S18). Altogether, these data clearly established that MRV μ2 protein by itself is sufficient to alter cellular AS, and that μ2 is able to alter cellular AS through both nuclear-dependent and nuclear-independent mechanisms.

### IP-MS of GFP-tagged μ2 reveals interaction with core components of the U5 spliceosomal snRNP

**Figure 5.**
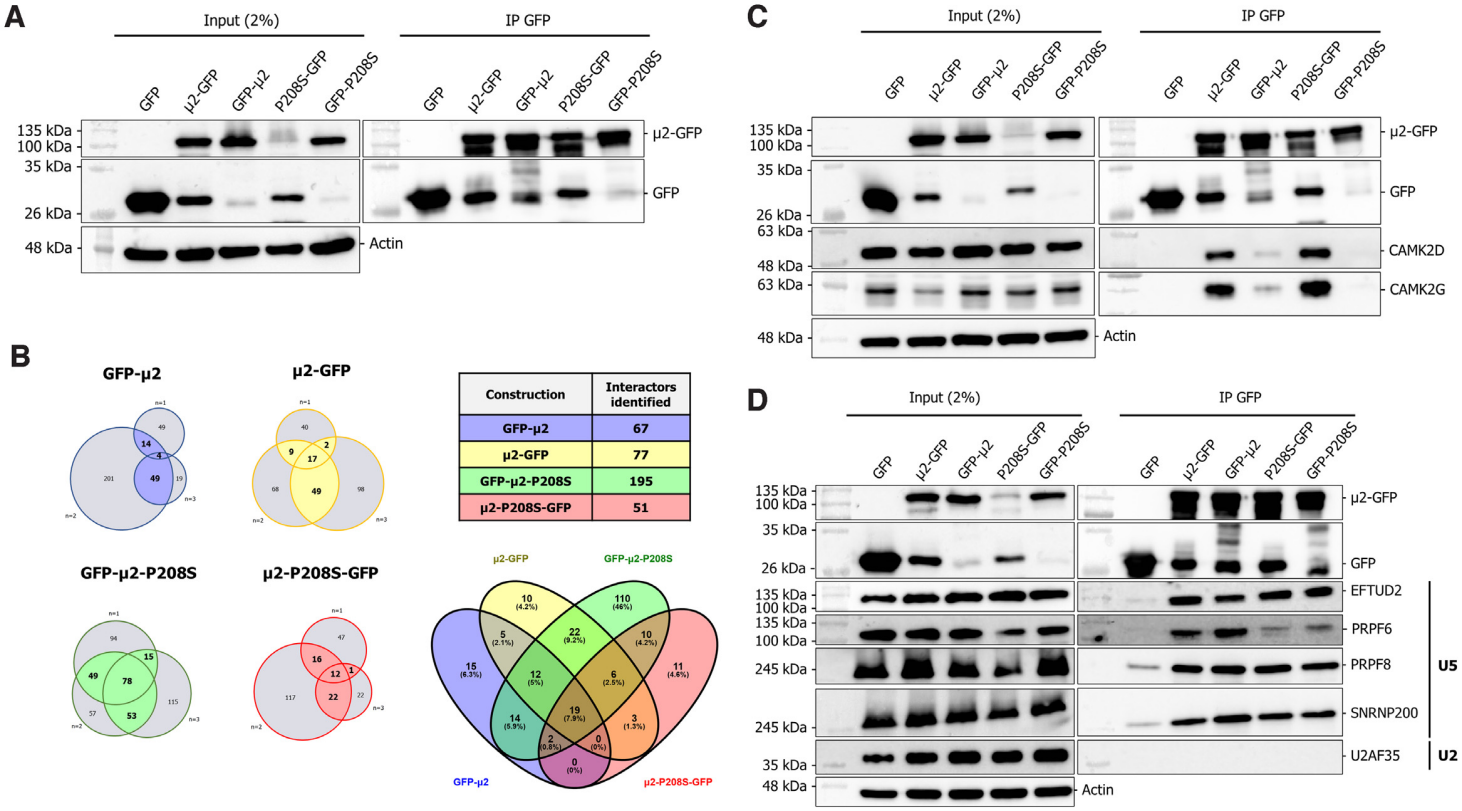
IP-MS of GFP-tagged μ2 reveals interaction with core components of the U5 spliceosomal snRNP. (**A**) Validation of the immunoprecipitation of the different μ2-GFP constructions. (**B**) Summary of the IP-MS results. On the left, the three independent replicates are shown for each construction, alongside the overlap between them. Only proteins identified in two or three independent replicates were selected as hits. On the top right, the number of identified hits for each construction is denoted. On the bottom right, the protein overlap between the different constructions is depicted using a Venn diagram. (**C**) Validation of the experimental μ2 IP-MS design using the CAMK2D and CAMK2G potential interactors by Co-IP and western blot. (**D**) Validation of the co-immunoprecipitation of MRV μ2 protein with components of the U5 snRNP by Co-IP and western blot. Input or IP fractions were resolved on SDS-page gels and submitted to a WB against GFP, EFTUD2, PRPF6, PRPF8, SNRNP200, U2AF35 or the loading control actin.

We next sought to determine which molecular mechanism(s) is (are) used by this viral protein to trigger such changes. Since numerous viral proteins interact with spliceosomes and splicing factors through direct protein-protein interaction (38,39,73,74), we first determined the cellular interactome of both μ2 and μ2-P208S using IP-MS. GFP-tagged constructions of μ2 and μ2-P208S in both C- and N- terminus were transfected in 293T cells alongside control GFP alone, and submitted to GFP pulldown. These constructions were readily expressed in 293T cells (Supplementary Figure S15 and S16) and were efficiently pulled down using GFP-Trap beads (Figure 5A). Since the μ2 protein binds RNA (11), lysates were DNAse and RNAse treated prior to IP to ensure interactions mediated by nucleic acids were not identified. Next, the immunoprecipitated proteins were subjected to tandem mass-spectrometry in three independent replicates to identify bound proteins. Each replicate was analyzed independently using SAINT (75), and interactors with a Saint score above 0.9 were considered statistically significant (Supplementary Figure S20). To ensure that only true partners were identified, we added an additional criterion of independent identification in at least two replicates, which allowed the identification of between 51 and 195 cellular partners for the four different constructs (Figure 5B). Moreover, each dataset had an important overlap with one another, as 39% of identified interactors were represented more than once (Figure 5B). Of those 61% that were unique, 46% belonged to the GFP-μ2-P208S dataset, which contained 2.5 to 4 times more identified proteins than the three other ones. The complete list of identified interactors is available in Supplementary Figure S21. To further validate our experimental procedure, we confirmed CAMK2D and CAMK2G as interacting partners of both WT and P208S mutant μ2, but only when the GFP moiety was fused in C-terminus, as determined in the MS results (Figure 5C; Supplementary Figure S21). These results underline the pertinence of analyzing interactors by tagging both ends of μ2, as in this case, the N-terminus GFP moiety is likely blocking the interaction interface of μ2 with these partners.

Having validated the experimental approach, we searched the interactome data for potential splicing factors and spliceosomal components involved in AS. The aforementioned overlap between the WT protein and the mutant suggests the P208S mutation has a limited impact on the interactome of the μ2 protein, and thus a difference in interacting partners might not be the explanation for their different impacts on AS. In light of these results, we focused our attention on the 19 proteins that were common between all constructions, which included the three core components of the U5 snRNP, i.e. EFTUD2, PRPF8 and SNRNP200 (Supplementary Figure S22). This strongly suggested that μ2 interacts with spliceosomal proteins, which could explain its impact on cellular AS. We validated that the different μ2 constructs indeed co-immunoprecipitated EFTUD2, PRPF8 and SNRNP200, with no impact of the P208S mutation on these interactions (Figure 5D). Moreover, another component of the U5 snRNP, PRPF6, which was identified in the GFP-μ2-P208S IP (Supplementary Figure S21), also immunoprecipitated with the WT μ2, albeit to a lesser extent (Figure 5D). However, μ2 failed to pulldown the U2AF35 protein, an auxiliary factor required for the recruitment of the U2 snRNP to the branch point, showing that μ2 specifically interacts with components from the U5 snRNP (Figure 5D). Finally, we also tested the ability of μ2 to pulldown the RNA components of the different snRNP in RIP-ddPCR experiment, and μ2 failed to pulldown the U5 RNA (Supplementary Figure S23). These data, taken together with the dispensable nuclear localization of μ2 for the modulation of some ASE (Figure 4E, Supplementary Figure S18), suggest that μ2 does not interact with the assembled snRNP in the nucleus, but rather with individual components in the cytoplasm before their import in the nucleus and assembly into a functional snRNP.

### U5 core components are required for MRV modulation of cellular AS and are reduced during infection by μ2

**Figure 6.**
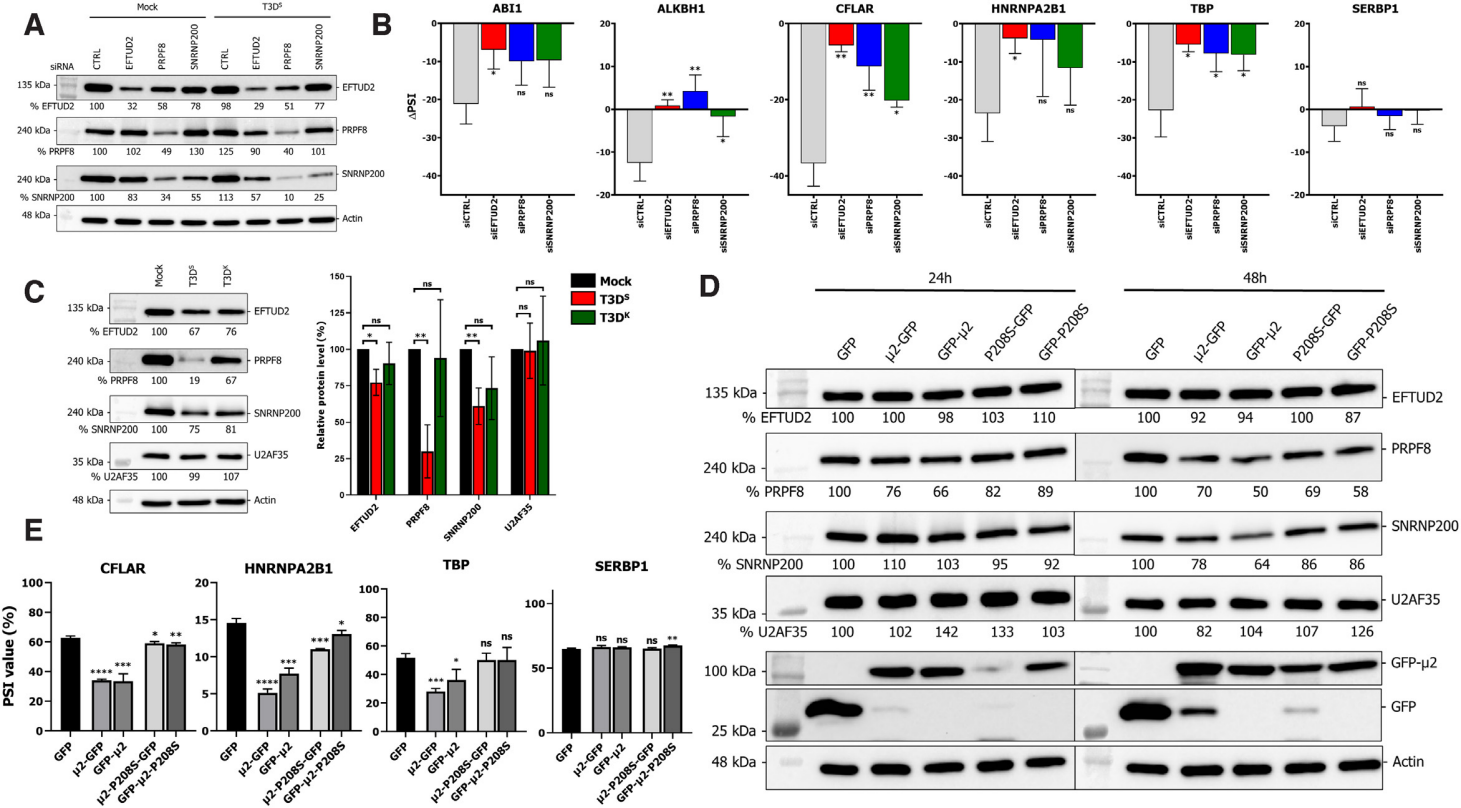
U5 core components are required for MRV modulation of cellular AS and are reduced during infection by μ2. (**A**) Validation of the KD efficiency for EFTUD2, PRPF8 and SNRNP200 by western blot in mock- and T3D^S^-infected L929 cells. (**B**) Difference in splicing (ΔPSI) between infected and mock L929 cells in control siRNA or EFTUD2, PRPF8, and SNRNP200 siRNA-treated cells. Respective standard deviation for the mock and infected cells were added when calculating the standard deviation for the ΔPSI. *n* = 3, biological replicates, unpaired two-tailed Student's t-test (ns, *P* > 0.05; *P ≤ 0.05; **P ≤ 0.01) comparing each condition against the control siRNA. (**C**) Protein levels of U5 snRNP components EFTUD2, PRPF8 and SNRNP200 in mock, T3D^S^ and T3D^K^ infected L929 cells. The membranes were H_2_O_2_-inactived and probed again against actin (EFTUD2, U2AF35) or vinculin (PRPF8, SNRNP200) as a loading control; a representative loading control is shown. On the right, the cumulative results for three western blots are summarized in a bar graph. The U2 snRNP protein U2AF35 was probed as a control. n = 3, biological replicates, unpaired two-tailed Student's t-test (ns, *P* > 0.05; *P ≤ 0.05; **P ≤ 0.01) comparing infected cells against control cells. (**D**) Impact of the ectopic expression of μ2 on U5 components protein levels at 24h and 48h in 293T cells. The U2 snRNP protein U2AF35 was probed as a control. The membranes were H_2_O_2_-inactived and probed again against actin (EFTUD2, U2AF35) or vinculin (PRPF8, SNRNP200) as a loading control; a representative loading control is shown. (**E**) Splicing profiles (PSI) of the *HNRNPA2B1*, *TBP*, and the negative control *SERBP1* AS minigene reporters upon expression of the different μ2 constructs at 48 h. PCR amplicons were resolved using capillary electrophoresis and quantified using relative fluorescence. *n* = 3, biological replicates, unpaired two-tailed Student's t-test (ns, *P* > 0.05; *P ≤ 0.05; **P ≤ 0.01; ***P ≤ 0.001; ****P ≤ 0.0001) against the GFP alone condition.

The interaction of μ2 with multiple central components of the U5 snRNP, namely EFTUD2, PRPF8 and SNRNP200, suggests that μ2 might exert its impact on cellular AS by affecting this crucial complex in the splicing reaction (38). To test this hypothesis, we individually depleted EFTUD2, PRPF8 and SNRNP200 in L929 cells using siRNA, and then infected the cells 16 h before harvesting the RNA to assess the ability of MRV to modulate AS in the absence of these components of the splicing machinery. We could readily reduce the levels of the three proteins between 50 and 75% (Figure 6A). Then, we calculated the difference in cellular AS upon infection by subtracting the PSI in infected cells from the PSI in control cells for each siRNA condition (ΔPSI). By doing so, we could isolate MRV’s impact in the experiment with no regard on the impact of the siRNA on these ASE. A drastic reduction of MRV’s ability to induce changes in the studied ASE was observed when either EFTUD2, PRPF8 or SNRNP200 was reduced (Figure 6B). However, knockdown of U5 core components did not enhance or affect the capacity of the virus to alter the splicing of the *SERBP1 ASE* negative control. Moreover, we monitored *M1* and *S1* viral RNA levels (Supplementary Figure S24), and μ2 and σ3 viral protein levels (Supplementary Figure S25) in infected cells to ensure that the knockdown of U5 components did not reduce viral replication under these conditions. Taken together, these data suggest that μ2 exerts its ability to alter the splicing of these ASE through components of the U5 snRNP.

To further understand the importance of the U5 snRNP in the control of AS for these ASE, we analyzed the impact of the reduction of these U5 components in uninfected cells. As shown for some of these ASE, depleting core components of the U5 snRNP led to more skipping of the ASE, as the PSI decreases (Supplementary Figure S26). This was particularly marked for the ASE in *ALKBH1* and *TBP*, with reduction of 10–20% in PSI upon knockdown. Interestingly, these ASE all show a reduction in inclusion and more exon skipping during infection (Figures 3 and 6B), which suggests MRV and μ2 might alter normal U5 snRNP function to alter the splicing of these ASE. We validated in immunofluorescence that there was no drastic relocalization of these proteins during MRV infection (Supplementary Figure S27). Next, we monitored protein levels for the main U5 components (EFTUD2, PRPF8, SNRNP200) in control and L929-infected cells, comparing again T3D^S^ with T3D^K^ as a prototypic virus harboring the P208S mutation in μ2. Surprisingly, T3D^S^ infection led to a striking reduction in PRPF8 (80%) protein levels, and a more modest reduction in EFTUD2 (35%) and SNRNP200 (25%). (Figure 6C). Interestingly, T3D^K^ reduced SNRNP200 and EFTUD2 levels in a similar fashion, but had only a modest impact on PRPF8, further strengthening our hypothesis that these two viruses affect the U5 snRNP differently, which lead them to also affect cellular AS in a different fashion (Figure 3). These differences in levels upon T3D^S^ infection were all statistically significant with three independent biological replicates, but not for the T3D^K^ infection (Figure 6C). Monitoring protein levels for U2AF35 revealed no reduction in this auxiliary spliceosomal protein, underlining that MRV specifically affect the levels of only some spliceosomal components in the U5 snRNP. Moreover, the U5 snRNA was the only spliceosomal RNA affected at the RNA level by MRV infection, further confirming that we identified the precise snRNP affected by MRV infection (Supplementary Figure S28).

Since U5 components were reduced in MRV-infected cells, we directly tested if the expression of μ2 was sufficient to alter the protein levels of those U5 proteins. GFP alone, μ2-GFP or the P208S mutants were transfected in 293T cells, and cells were harvested at 24 and 48 h to test EFTUD2, PRPF8, and SNRNP200 protein levels. At 24 h, a 20% reduction of PRPF8 levels could already be detected (Figure 6D). This reduction was further increased at 30–50% after 48 h for PRPF8, and SNRNP200 protein level was also reduced 15–35% by μ2 expression at that time point. The P208S mutation did not affect the reduction of either PRPF8 or SNRNP200, suggesting the P208S mutant retains its ability to reduce PRPF8 levels, as seen during T3D^K^ infection (Figure 6C). Once again, we could not detect any difference in the U2 auxiliary protein U2AF35. Quantification of three independent experiments confirmed a statistically significant reduction at 24 h for PRPF8 and for both PRPF8 and SNRNP200 at 48 h (Supplementary Figure S29). Taken together, these data suggest that μ2 is the main MRV protein responsible for reducing protein levels of the U5 snRNP core components during infection. Furthermore, the increased reduction at 48 h of U5 snRNP PRPF8 and SNRNP200 protein levels suggests the impact of the ectopic expression of μ2 on AS might be increased at this time point. As we previously monitored μ2’s impact on AS minigenes at 24h, we reassessed this ability at 48 h with the previously inconclusive *HNRNPA2B1* and *TBP* reporters (Figure 4C). Very reminiscent of the case of the *CFLAR* minigene (Figure 4C), μ2 expression alone modulated the AS of both these minigenes, but the P208S had a much more limited impact (Figure 6E), highlighting that the reduction of U5 snRNP components are correlated with an increased impact on cellular AS attributable to the μ2 protein. The impact on the *CFLAR* minigene was also increased at 48 h compared to 24 h (Figures 4C and 6E). Altogether, these results reveal that MRV modulation of these ASE involves the reduction of core components of the U5 snRNP through the action of the μ2 protein.

## DISCUSSION

### Involvement of μ2 in reducing U5 snRNP components during MRV infection to modulate cellular AS

**Figure 7.**
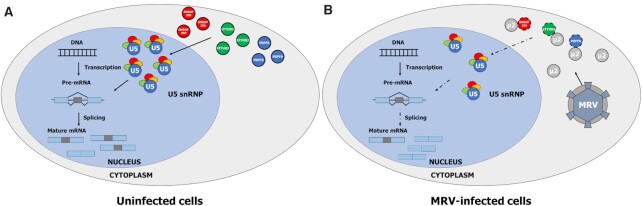
Model depicting how MRV infection leads to alterations in the host cell AS. In normal conditions (**A**), U5 main components EFTUD2, PRPF8, and SNRNP200 are translated in the cytoplasm and imported in the nucleus. The U5 snRNP levels are decreased due to the normal turnover of the complex, but this decrease is balanced by the import of the protein components and assembly of new U5 snRNP, allowing the functional U5 snRNP level to stay at equilibrium. Upon infection (**B**), expression of MRV viral protein μ2 in the cytoplasm disturbs the capacity of the cell to produce EFTUD2, PRPF8 and SNRNP200 protein through a yet to define mechanism. This shutdown prevents the cell from replenishing the diminution of the U5 snRNP due to the normal turnover, and functional U5 snRNP level diminishes. This affects the capacity of the cell to regulate its AS, and leads to AS changes observed during MRV infection.

In this study, we demonstrated that the μ2 protein is a key determinant for the modulation of AS during MRV infection. We showed that upon MRV infection, the μ2 protein exerts its effect on some specific ASE by reducing the levels of core components of the U5 snRNP (Figure 7). The μ2 protein is not sequestering U5 snRNP protein in the cytoplasm, as immunofluorescence of MRV-infected cells failed to show any defect in localization for EFTUD2, PRPF8, and SNRNP200 (Supplementary Figure S27). Although we demonstrated that the reduction of U5 snRNP core components protein levels during infection was attributable to μ2, and that μ2 interacts with these proteins, how μ2 exerts this effect on these U5 snRNP proteins remains elusive. However, the data presented herein supports the hypothesis that μ2 is not able to induce the degradation of U5 components assembled in the mature U5 snRNP in the nucleus, but suggests it rather impairs the renewal of these proteins. First, μ2 mutants unable to accumulate in the nucleus remain capable of altering AS (Figure 4E, Supplementary Figure S18), suggesting nuclear localization is not required for all the modulation of AS. Second, transient expression of μ2 shows a very limited reduction of these components at 24h and a modest reduction at 48h (Figure 6D), arguing against the degradation of the bulk nuclear pool of these proteins. This suggests that the reduction requires time, and supports a defect in renewal as more probable than the degradation of already translated proteins. Third, μ2 does not interact with the assembled U5 snRNP (Supplementary Figure S23). Altogether, the data presented in this study suggest that μ2 affects either the transcription, stability, export, or translation of the RNA for these U5 proteins, or degradation of the newly synthesized proteins in the cytoplasm before their nuclear import.

Since μ2 mutants unable to accumulate in the nucleus also exert an activity on AS (Figure 4E, Supplementary Figure S18), transcription and nuclear export of the RNA seems less likely than RNA stability in the cytoplasm, translation, or degradation of the newly synthesized proteins. In the RIP-ddPCR experiment, we saw that μ2 was bound non-specifically to all cellular mRNA that we tested (Supplementary Figure S30). This result supports the RNA binding activity of μ2 demonstrated before (11), and further suggests it might impair translation by interacting with cellular mRNA. A translational block was shown long ago in MRV-infected cells (1,2) that favours MRV RNA to the detriments of cellular mRNA. Although it was attributed to σ3 and its antagonism of the PKR protein (76,77), a contribution of the μ2 protein might have been overlooked. Supporting this hypothesis is the identification of numerous proteins from the large ribosomal subunit in the μ2 IP-MS (i.e. *RPL3, RPL5, RPL7A, RPL8, RPL9, RPL10A, RPL12, RPL28, RPL31* and *RPL35A;*Supplementary Figure S21). Care must be taken as ribosomal proteins are frequent contaminants in IP-MS (78), but this result warrants further study to determine if μ2 does indeed contact the large subunit of the ribosome and affects translation. Interestingly, the μ2 protein itself is ubiquitinylated (79), and μ2 pulldowns numerous E3 ubiquitin ligases (i.e. *MYCBP2, HERC2, UBR5, PRPF19, UBE3C* and *TRIM27*; Supplementary Figure S21). Since ubiquitination is the principal pathway of degradation for nuclear proteins (80), this raises the hypothesis that μ2 might contact E3 ubiquitin ligases and U5 snRNP components to induce their degradation.

One limitation of our IP-MS approach is that it is very likely that some of the U5 proteins we identified (EFTUD2, PRPF8 and SNRNP200; Figure 5D) are direct interactors of μ2, and some are co-immunoprecipitated by the direct interactors bound with μ2. However, our data clearly involve the U5 snRNP in MRV modulation of cellular AS, as KD of any of U5 snRNP core proteins reduce the ability of MRV to alter AS (Figure 6B). Surprisingly, one might expect that this would result in complete abrogation of both constitutive and alternative splicing, as U5 is required for the splicing reaction. This study thus points towards a possible implication of the U5 snRNP in controlling AS, and a high tolerance to reduction of the U5 snRNP levels without any drastic effect on constitutive splicing. Recent ENCODE data bolsters this hypothesis, as only between 15% to 25% of affected ASE following KD of EFTUD2, PRPF8 or SNRNP200, are retained introns (81). Therefore, the impact of reducing specific components of U5 snRNP and/or reducing the total U5 snRNP functional pool translates principally to an impact on AS, and not on constitutive splicing. Further studies will need to address the precise role of the U5 snRNP in regulating AS, and how this allows MRV to modulate cellular AS during infection.

### Importance of the U5 snRNP in viral infection

The identification of components of the U5 snRNP as interactors of MRV μ2 protein might seem surprising, as these cellular components are mainly nuclear, whereas MRV replicates in the cytoplasm. However, some examples of cytoplasmic virus proteins interacting with U5 snRNP components exist, such as the NS5 protein from Dengue virus. This RNA-dependent RNA polymerase (RdRp) interacts with CD2BP2 and DDX23 from the U5 snRNP, allowing modulation of cellular AS during infection benefiting the replication of Dengue virus (38). Another RdRp, 3D^pol^ from EV71 (a picornavirus), directly interacts with PRPF8 to also induce changes in cellular AS (39). In both cases, these RdRp are directly located in the nucleus of the cell during infection. In the case of MRV, our data support that the usurpation of these components is not nuclear, but rather cytoplasmic, suggesting a new mechanism never described before (see *Insight into the molecular impact of the P208S substitution on μ2 activity on cellular AS* for further explanations). Moreover, it is intriguing that the two know examples of viral proteins interacting with U5 components are RdRp, as μ2 is forming the MRV replicase complex alongside λ3, MRV’s RdRp. The fact that multiple proteins from different RNA viruses interact with U5 proteins suggests that U5 might play an important role in virus-host interactions. PRPF8 has previously been demonstrated to influence viral replication, as during influenza infection, it is induced by viral proteins and act as a proviral factor increasing viral production (82). Moreover, numerous studies support the involvement of U5 snRNP proteins in the interferon pathway (83). Indeed, both EFTUD2 and SNRNP200 have been shown to act as RNA sensors in the cytoplasm to allow the induction of the IFN response pathway during viral infection (84,85). Furthermore, EFTUD2 also controls the splicing of *MYD88*, which is crucial in allowing the transduction signal from Toll-like receptors to the nucleus to induce IFN production (86). The role of U5 core components (EFTUD2, PRPF8, SNRNP200) in MRV replication and IFN induction could be further addressed in follow-up studies. Nevertheless, we did notice in the present work a small increase in μ2 protein levels (Supplementary Figure S25) and S1 viral RNA levels (Supplementary Figure S24) upon KD of PRPF8, suggesting it might exert an antiviral role. The suspected implication of U5 snRNP proteins in MRV replication is further strengthened by the recent identification of multiple components of the U5 snRNP (*EFTUD2*, *PRPF8*, *SNRNP200*, and *TXNL4A*) in a CRISPR-screen of host factors required for MRV replication and cell killing (87).

### Insight into the molecular impact of the P208S substitution on μ2 activity on cellular AS

We identified a key polymorphism in μ2, the serine or proline at position 208, that drastically alters the impact of μ2 on cellular AS (Figure 3). This position has been linked to a number of phenotypes before. Indeed, the P208S polymorphism controls the viral factories morphology (5–9,66), the blockade of the IFN signaling in cardiomyocytes (67,68), the ability of μ2 to locate to nuclear speckles (45), the ubiquitination of μ2 (79) and the oncolytic potential of MRV (16). Since we identified a new phenotype linked to this position, we sought to understand how this position affected the impact on cellular AS. Our data showed no drastic impact of the P208S substitution on the interactome of μ2 (Figure 5B), nor on μ2’s ability to reduce PRPF8 and SNRNP200 protein levels in transient expression (Figure 6D). However, we did show that the P208S impairs the ability of μ2 to accumulates in the nucleus during transient transfection (Figure 4A), although our results with μ2 mutants showed that nuclear localization was not necessary for the modulation of all ASE tested (Figure 4E and Supplementary Figure S18). The significance of the accumulation of μ2 in the nucleus during transient transfection is unclear, as we and others have failed to locate the μ2 in the nucleus during infection using immunofluorescence (9). An additional explanation for the P208S defective mutation could be that this mutation simply decreases the levels of μ2 during infection, likely through increased ubiquitination, and thus insufficient μ2 levels could lead to all these aforementioned loss-of-function phenotypes. We did observe that viruses harboring a P208 present higher μ2 levels than the ones harboring a S208 (Supplementary Figure S31), supporting this hypothesis. Further studies will be required to adequately compare biochemical properties of μ2 proteins bearing P208 or S208, and the impact of μ2 levels on its activities during viral replication.

We have also generated mutants of the μ2 protein with an impaired nuclear localization (Figure 4D) that are useful molecular tools to define the role of nuclear localization in μ2 activity. We do not claim to have mutated the nuclear localization signal of μ2, as several different mutants present impaired nuclear localization; we rather think we affected the structure of the protein sufficiently to impair the nuclear accumulation. For instance, the 100-AALAAALMLAA-110 mutant is no longer binding to microtubules; it is also the case of the 545-ALAIAA-550 mutant bearing the GFP in N-terminus (Figure 4D) which supports a more global misfolding rather than a specific mutation to the nuclear localization signal. Designing such mutants have been challenging in the absence of a structure for μ2, but the structure has been recently solved using Cryo-EM in the viral particle (88). The structural information will greatly help the designing of molecular tools such as truncation mutants and isolated μ2 domains that will help a better understanding of the μ2 protein and the impact of the P208S substitution.

Finally, one last remark must be made concerning the impact of the P208S on cellular AS. As the RNA-Seq data have been generated using T3D^S^ that harbors the P208 (44), the selection of ASE to analyze was biased for events modulated strongly by μ2 with a proline at 208. The limited impact of the μ2-S208 on these ASE must be regarded in light of how ASE were selected; it is highly possible that some events (such as CDKN2AIP, Supplementary Figure S10) are modulated more efficiently by μ2-S208 but were not further studied because of the limited impact of T3D^S^ on them. Future work could be done to get a thorough understanding by comparing the impact on AS for T3D^S^, T3D^K^, and their isogenic virus with the P208S and S208P substitution, respectively, by a non-biased approach such as RNA-Seq. Furthermore, we concentrated our efforts on some ASE that were modulated by MRV through the U5 snRNP. To which extent this mechanism accounts for the other AS changes observed previously, and if additional mechanisms are at play during MRV infection to alter AS, remains to be further studied.

### Concluding remarks

This study underlines a novel mechanism utilized by viruses to modulate cellular AS during infection involving the U5 snRNP of the spliceosome. The identification of a polymorphism in μ2 that controls both the impact of MRV on cellular AS and its oncolytic potential raises the possibility that the ability to impact cellular AS might be beneficial for the oncolytic potential (16). It has previously been shown that SRSF2 splicing factor restrict herpes simplex virus type 1 oncolytic activity, further suggesting cellular AS might dictate the oncolytic potential of viruses (89). Further studies should increase our understanding of the ability of other viruses to alter cellular AS by reducing spliceosomal protein levels, and if this modulation shape MRV therapeutic potential as an oncolytic virus.

## DATA AVAILABILITY

The mass spectrometry proteomics data have been deposited to the ProteomeXchange Consortium via the PRIDE (90) partner repository with the dataset identifier PXD029701. The Venn diagram was produced using Venny2.1 (*Oliveros, J.C. (2007-2015) Venny. An interactive tool for comparing lists with Venn's diagram;*https://bioinfogp.cnb.csic.es/tools/venny/index.html).

## Supplementary Material

gkac272_Supplemental_File
