# Supplementary material for: Reovirus μ2 protein modulates host cell alternative splicing by reducing protein levels of U5 snRNP core components
Source: Nucleic Acids Res. 2022 Apr 30;50(9):5263–81. doi: 10.1093/nar/gkac272 (PMC9122528; doi:10.1093/nar/gkac272)

Supplementary Tables and Figures

Reovirus µ2 protein modulates host cell alternative splicing by reducing protein levels of U5 snRNP core components

Simon Boudreault, Mathieu Durand, Carole-Anne Martineau, Jean-Pierre Perreaul[1], Guy Lemay and Martin Bisaillon

Table S1. qPCR and ddPCR primers

Target	Housekeeping gene	Species	Assays	Primers	
				Fw	Rv
DDX60		Mouse	qPCR – RIG-I KD, bystander	5′-CCACCACAGTTCCATGAGTGCC-3′	5′-TGATTCCCAAAGCGAGTGTTCCA-3′
IFNB1		Mouse	qPCR – RIG-I KD, bystander	5′-ACACTGCCTTTGCCATCCAAGA-3′	5′-ACACTGTCTGCTGGTGGAGTTCA-3′
MX1		Mouse	qPCR – RIG-I KD, bystander	5′-GAAGGAGAGGAGTGGAGAGGCA-3′	5′-GCTTATCACTGATCCCCAGGCC-3′
Rnu1a1 (U1)		Mouse	qPCR – infected cells (S28)	5′-TTTTCCCAGGGCGAGGCTTA-3′	5′-CCCCACTACCACAAATTATGCA-3′
Rnu2-10 (U2)		Mouse	qPCR – infected cells (S28)	5′-TGGTATTGCAGGGCTAAGATCA-3′	5′-CAGGTCGATGCGTGGAGT-3′
Gm24265 (U4)		Mouse	qPCR – infected cells (S28)	5′-CTTTGCGCAGTGGCAGTATC-3′	5′-CCAATGCCGACTATATTGCAAGTC-3′
Rnu5g (U5)		Mouse	qPCR – infected cells (S28)	5′-CCTTGTCAAGACAAGGCCTC-3′	5′-CTCTGGTTTCTCTTCAGATCGTATAAATC-3′
Gm25064 (U6)		Mouse	qPCR – infected cells (S28)	5′-CGCTTCGGCAGCACATATAC-3′	5′-AATATGGAACGCTTCACGAATTTGC-3′
PSMC4	✓	Mouse	qPCR – RIG-I KD, bystander, time course, reassortant viruses, U5 KD	5′-CCCAGGAGGAGGTGAAGCGG-3′	5′-GGTCGATGGTACTCAGGATGCG-3′
PUM1	✓	Mouse	qPCR – RIG-I KD, bystander, time course, reassortant viruses, U5 KD	5′-TGCCAGTCTCTTCCAGCAGCA-3′	5′-TGATTTGGGGTCAAAGGACGTTGG-3′
TXNL4B	✓	Mouse	qPCR – RIG-I KD, bystander, time course, reassortant viruses, U5 KD	5′-CCCTCTACCGTATTTTTCTTCAATGGGC-3′	5′-AGTTTTCCCCTCATCGCTCCCC-3′
S1		MRV	qPCR – RIG-I KD, bystander, time course, reassortant viruses, U5 KD	5′-AGCGCGCAGCCTAAATGGTT-3′	5′-ACGTAGATGCCGGGTCTGCT-3′
M1		MRV	qPCR – RIG-I KD, bystander, time course, reassortant viruses, U5 KD, RIP-ddPCR	5′-GGTCGGATGGATCCTCGCCT-3′	5′-TCGAGTCCCTGGGTGATCCG-3′

Table S1. qPCR and ddPCR primers (cont'd)

Target	Housekeeping gene	Species	Assays	Primers	
				Fw	Rv
ABI1		Human	RIP-ddPCR	5′-TCAGAAACCGCCAAGTCCTCCC-3′	5′-GCTGACTTCCAAGCCTAGCAGG-3′
ACTB		Human	RIP-ddPCR	5′-AACCCCAAGGCCAACCGCGA-3′	5′-GGCCAGAGGCGTACAGGGATAG-3′
ALKBH1		Human	RIP-ddPCR	5′-GGGGTCATCGACTTCTCGGC-3′	5′-CACTTGCTGACGGGCTGAAGAC-3′
CFLAR		Human	RIP-ddPCR	5′-GGCAGAGATTGGTGAGGATTTGGA-3′	5′-CCAACTCAACCACAAGGTCCAAGA-3′
GAPDH		Human	RIP-ddPCR	5′-CGAGCCACATCGCTCAGACAC-3′	5′-ATGGCAACAATATCCACTTTACCAGAGT-3′
HNRNPA2B1		Human	RIP-ddPCR	5′-GCAGGAAGTTCAGAGTTCTAGGAGTG-3′	5′-AAATTTCCACCGCCACCACG-3′
IL34		Human	RIP-ddPCR	5′-GCCCTTGACGCAGAATGAGGAG-3′	5′-ACGTTGGCGATTCTGAACACCC-3′
MRPL19	✓	Human	RIP-ddPCR	5′-AAGGAGAAAAGTACTCCACATTCCAGAG-3′	5′-TGGGTCAGCTGTAGTAACACGA-3′
RNU1-1		Human	RIP-ddPCR	5′-TCACGAAGGTGGTTTTCCCA-3′	5′-GAACGCAGTCCCCCACTACC-3′
RNU2-1		Human	RIP-ddPCR	5′-TTTGGCTAAGATCAAGTGTAG-3′	5′-AGCTCCTATTCCATCTCCCT-3′
RNU4-1		Human	RIP-ddPCR	5′-CAGTATCGTAGCCAATGAGGTC-3′	5′-TGTCAAAAATTGCCAGTGC-3′
RNU5D-1		Human	RIP-ddPCR	5′-GCTCTGGTTTCTCTTCAAATC-3′	5′-AAAAATTTGCTTGAAACTCAAA-3′
RNU6-1		Human	RIP-ddPCR	5′-TGCTCGCTTCGGCAGCAC-3′	5′-ATGGAACGCTTCACGAATTTGC-3′
SERBP1		Human	RIP-ddPCR	5′-AAGACCAGAAAGGCGACCACCT-3′	5′-GCCCCCTCGACCTCTTCCAA-3′
TBP		Human	RIP-ddPCR	5′-GGGGTCATCGACTTCTCGGC-3′	5′-CACTTGCTGACGGGCTGAAGAC-3′

Table S2. Primers and peaks analyzed by AS-PCR in mouse cells

Gene	Primers Fw	Primers Rv	ASE present in this region	Expected peak(s) (bp)	Detected peak(s) (bp)	ASE quantified	Corresponding detected peak(s) (bp)
ABI1	5′-TGGAAGTAGTG GAGGAAGCGGA-3′	5′-GGTGGTGGTG GAGTTGGGCTAT-3′	Multiple exon cassette (2)<br>3′-SS	129, 303, 306, 390, 393	138, 313, 366, 424, 468, 564, 688, 894	Exon cassette	313, 424
ALKBH1	5′-GGAAGCTTTTC CGCTTCTACCG-3′	5′-GTTCAAGTCCC ACCCTCTCGG-3′	Multiple exon cassette (2)	180, 318, 391	191, 323, 419	Multiple exon cassette (2)	191, 323
CDKN2AIP	5′-GCTCCAATCC ATACCACACGA-3′	5′-AACAAATGAC GCTGAACCACG-3′	5′-SS	371, 402	388, 425, 482	5′-SS	388, 425
CFLAR	5′-CAGAGTGAGGC GGTTTGACCTT-3′	5′-TGGACTGGGTG TACTTCTGGAT-3′	Exon cassette	238, 344, 704, 869	244, 355	Exon cassette	244, 355
EIF4A2	5′-GGGATTGACG TGCAACAAGTG-3′	5′-CCACACCTTTCC TCCCAAATCG-3′	Exon cassette<br>Intron retention	112, 219, 928	115, 232, 243, 264, 302	Exon cassette	115, 232
HNRNPA2B1	5′-ATGGATACGGA AGTGGACGTGG-3′	5′-TCCTGCTACCCC CAAAGTTTCC-3′	Exon cassette	162, 282	170, 290, 549	Exon cassette	170, 290
IL34	5′-ACGTGAGTGA GCGAGAGCTTC-3′	5′-AAGCAGTTGTC CAGCAAGGCT-3′	Exon cassette	256, 374	272, 417	Exon cassette	272, 417
SERBP1	5′-AAGGCGAGTTGG AAGAAGACCC-3′	5′-TTCCCCAGTTGTG AGAGCCG-3′	Exon cassette<br>Intron retention<br>5′-SS, 3′-SS	172, 341, 359, 470	356, 381, 443, 491	3′-SS	356, 381
TBP	5′-ACGGACAACTGC GTTGATTTTC-3′	5′-AGGAGAACAATT CTGGGTTTGATCA-3′	Exon cassette	120, 288	121, 288, 481	Exon casette	121, 288

Table S3A. Primers and peaks analyzed by AS-PCR in human cells - AS minigene reporters

Gene	Primer		ASE present in this region	Expected peak(s) (bp)	Detected peak(s) (bp)	ASE quantified	Corresponding detected peak(s) (bp)
	Fw	Rv					
ALKBH1	5′-GGAAGCTTTTC CGCTTCTACCG-3′	5′-TAGAAGGCACA GTCGAGG-3′	Multiple exon cassette (2)	284, 422, 495	284, 426, 489	Exon cassette	284, 422
CFLAR	5′-CAGAGTGAGGC GGTTTGACCTT-3′	5′-TAGAAGGCACA GTCGAGG-3′	Exon cassette	294, 400	292, 420	Exon cassette	292, 420
HNRNPA2B1	5′-ATGGATACGGA AGTGGACGTGG-3′	5′-TAGAAGGCACA GTCGAGG-3′	Exon cassette	201, 362	241, 255, 400	Exon cassette	255, 400
TBP	5′-ACGGACAACTG CGTTGATTTTC-3′	5′-TAGAAGGCAC AGTCGAGG-3′	Exon cassette	173, 376	219, 392	Exon cassette	219, 392
SERBP1	5′-GGTGAAGGAG GTGAATTTTCAGT TGATAG-3′	5′-TAGAAGGCACA GTCGAGG-3′	Exon cassette<br>Intron retention<br>5′-SS, 3′-SS	304, 322	298, 331	3′-SS	298, 331

Table S3B. Primers and peaks analyzed by AS-PCR in human cells – Endogenous human ASE

Gene	Primer		ASE present in this region	Expected peak(s) (bp)	Detected peak(s) (bp)	ASE quantified	Corresponding detected peak(s) (bp)
	Fw	Rv					
ABI1	5′-TCGAGAAAACA GTGGTAGCAGT-3′	5′-CGGTGGAGTTG GACTATCAGCA-3′	Multiple exon cassette<br>3′-SS	100, 181, 187, 268, 274, 277, 355, 358, 361, 364, 442, 445, 491	105, 283, 360, 395, 458, 473, 667	Multiple exon cassette	105, 395
CDKN2AIP	5′-CAAAGTGACA GATGCTCCAACC-3′	5′-TGGCAGAGGTT TTTGCGTGATC-3′	5′-SS	139, 170	150, 187, 274, 284	5′-SS	150, 187
SERBP1	5′-AGACGAGTTGG AAGAAGACCTG-3′	5′-TTCCCCAGTTGT GAGATCCGC-3′	3′-SS	340, 358	179, 372, 393, 498, 599	3′-SS	372, 393

Figure S1. Relative mRNA levels for IFNB1, DDX60, and MX1 in siCTRL and siRIG-I mock and infected L929 cells. siRNA were transfected using RNAiMAX, and 56 h post-transfection, cells were infected with MRV ($T3D^S$) at a MOI of 50, or mock-infected. Cells were further incubated for 16 h before RNA was harvested using Qiazol, reverse-transcribed, and subjected to qPCR for IFNB1, DDX60, and MX1 with PSMC4, PUM1, and TXNL4B used as housekeeping genes. The first replicate in the mock siCTRL condition was fixed at 1, and the relative mRNA expression was calculated for all other samples relative to that one. n=3, biological replicates, two-way ANOVA with Dunnett's multiple comparisons test against the siCTRL condition for mock (in black) or infected cells (in red); (ns, $P>0.05$; ****, $P\leq0.0001$).

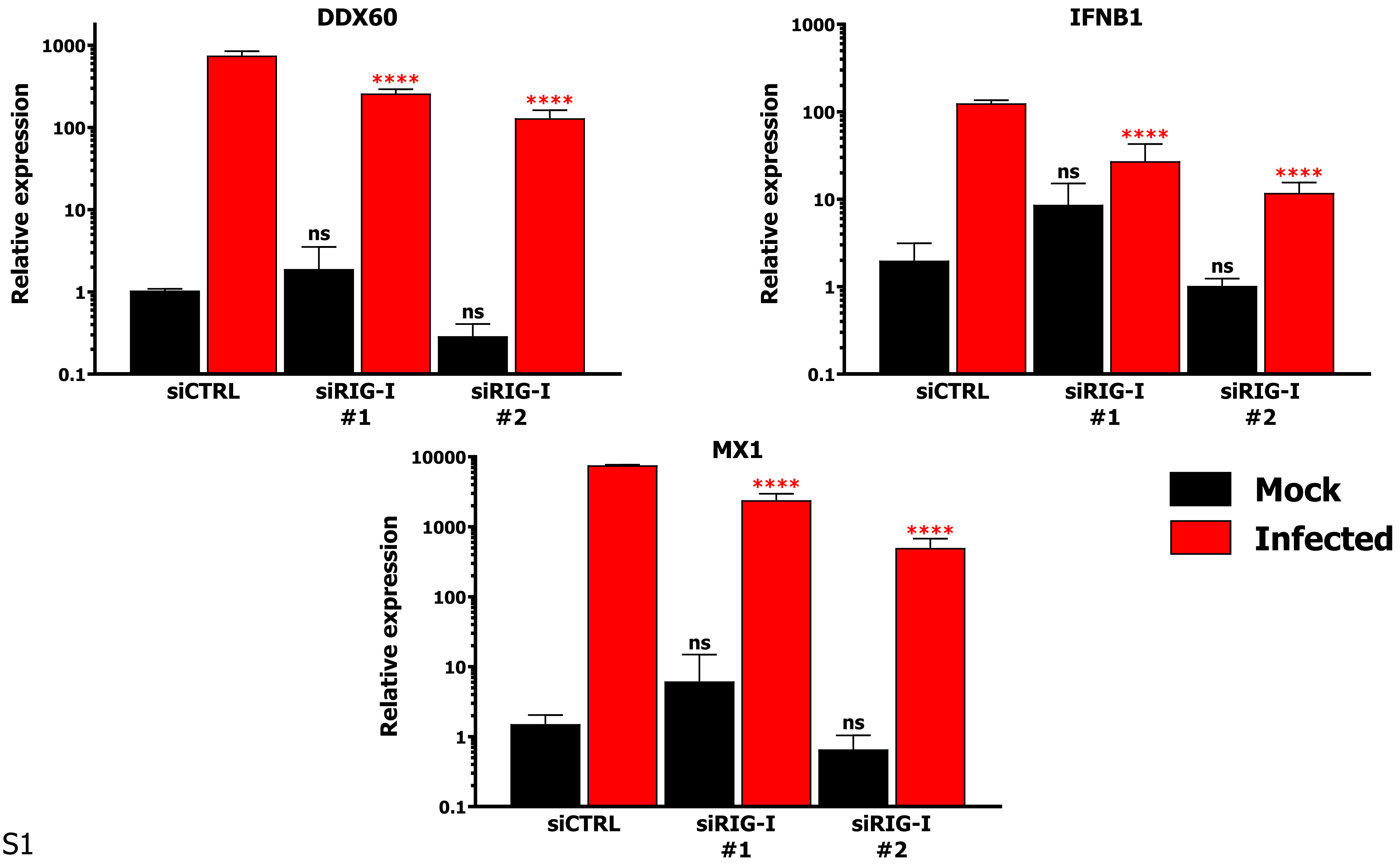
DDX60
IFNB1
MX1
Relative expression
1000
100
10
1
0.1
10000
ns
****
siCTRL
siRIG-I #1
siRIG-I #2
Mock
Infected

Figure S1

Figure S2. Design maps for the ASE analyzed by AS-PCR in mouse and human cells.

All RNA isoforms for each gene is depicted, with the region alternatively spliced where the primers bind. The ENSEMBL assembly MM88.GRCm38 (mouse) and Hg38.p12.109.20191206 (human) were used to generate the maps.

Mouse ASE

ABI1

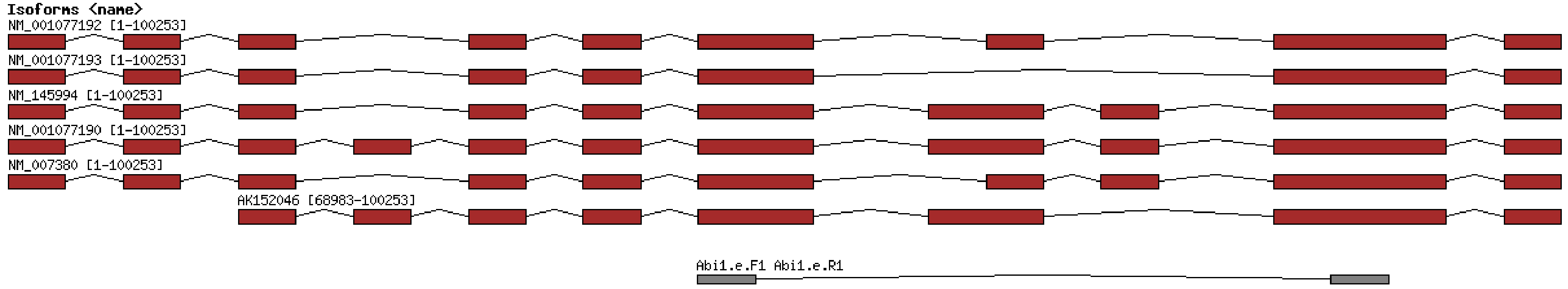

ALKBH1

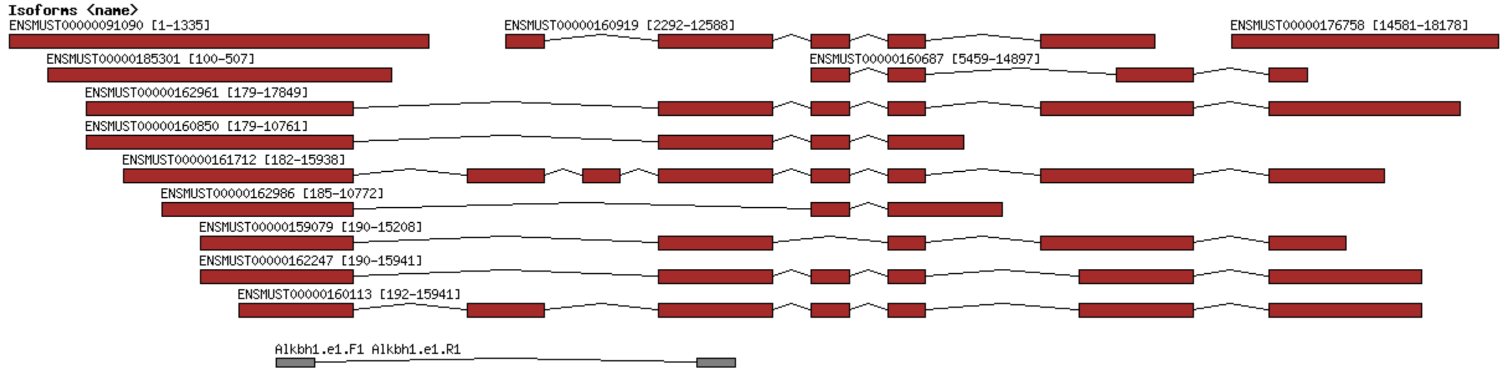

CDKN2AIP

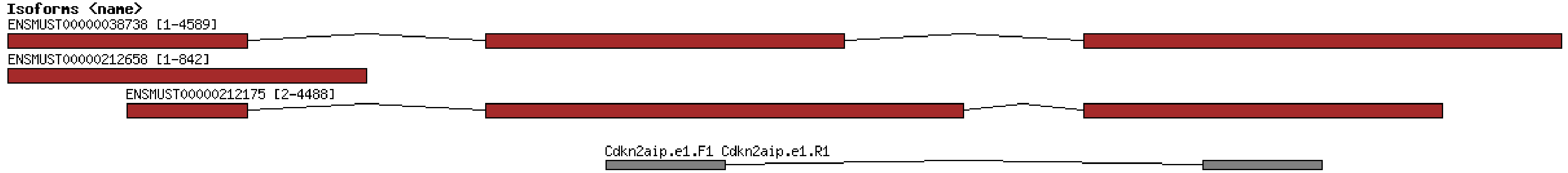

Figure S2

CFLAR

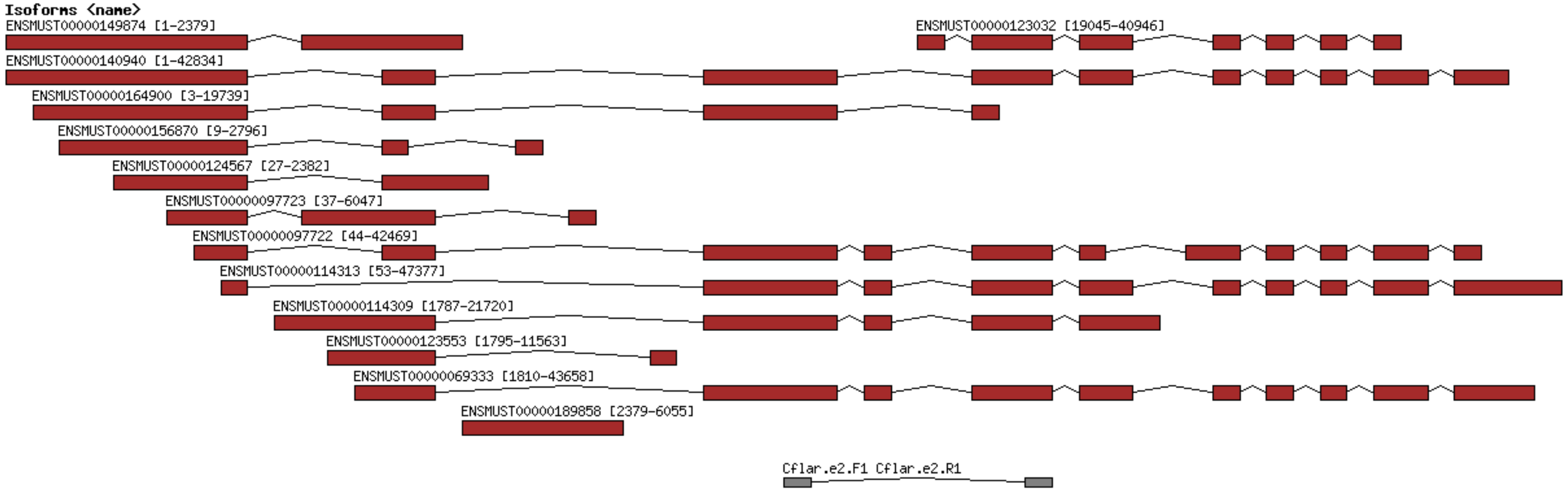

EIF4A2

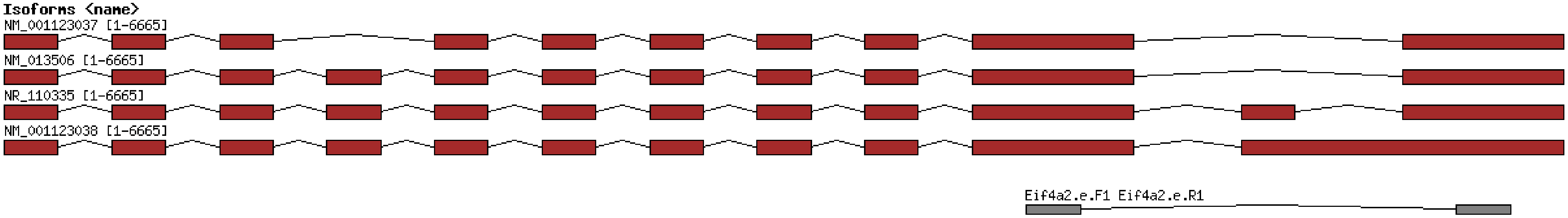

HNRNPA2B1

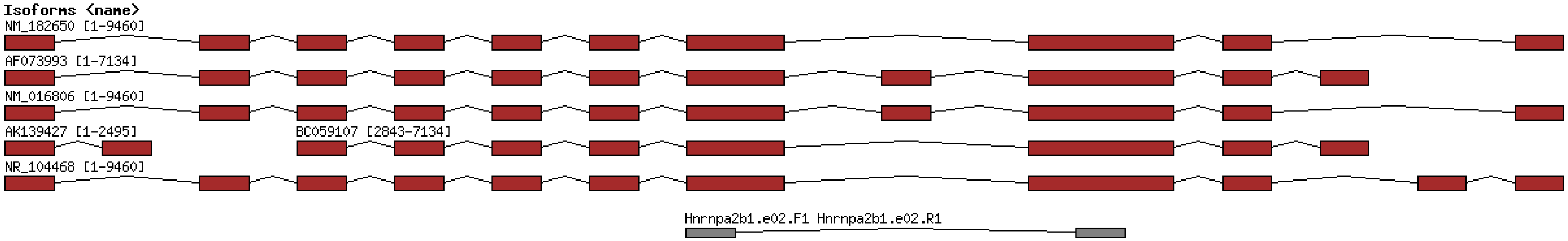

Figure S2 (cont’d)

IL34

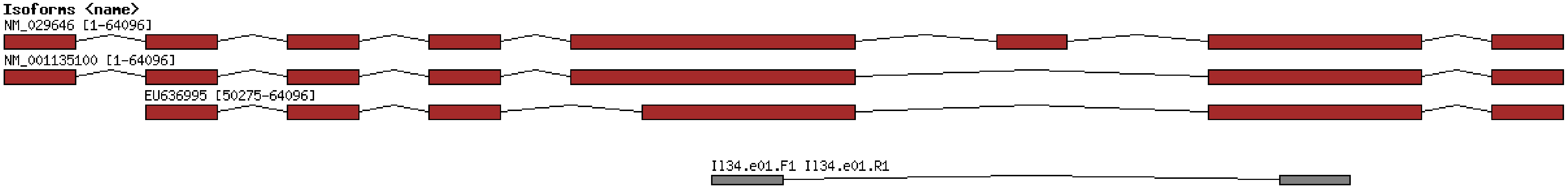

SERBP1

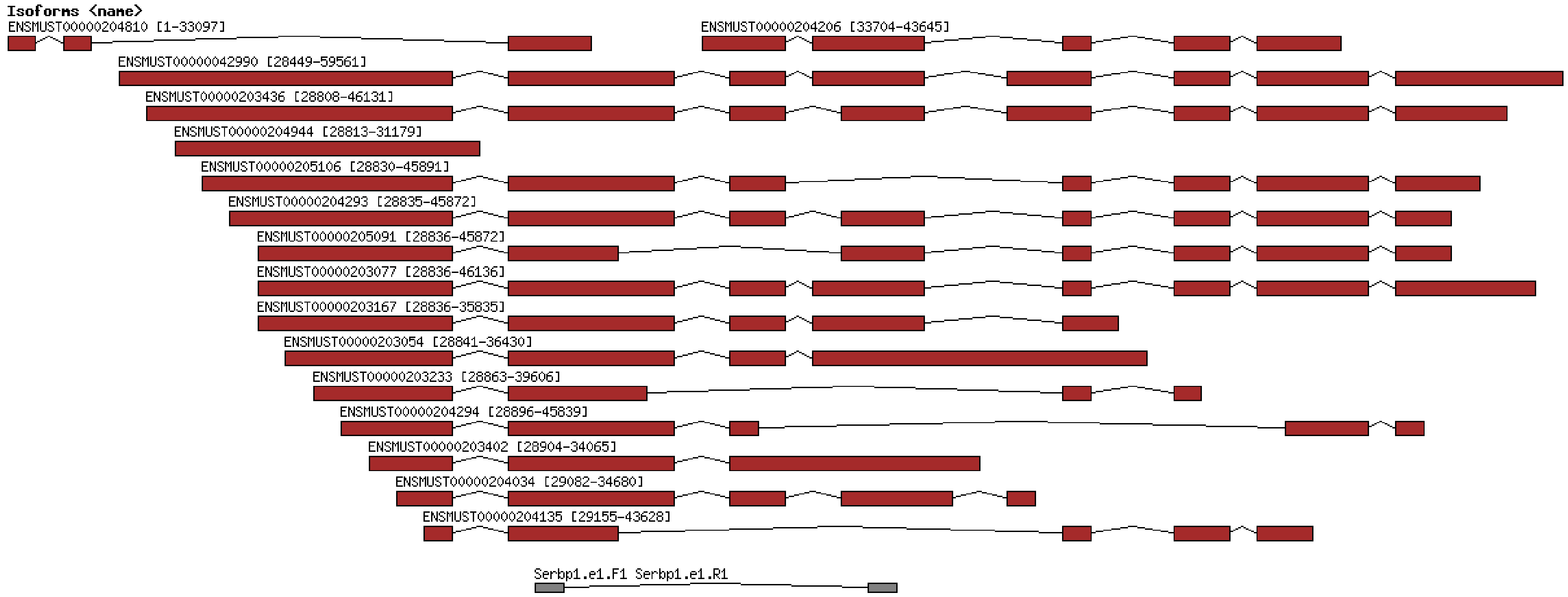

Figure S2 (cont'd)

TBP

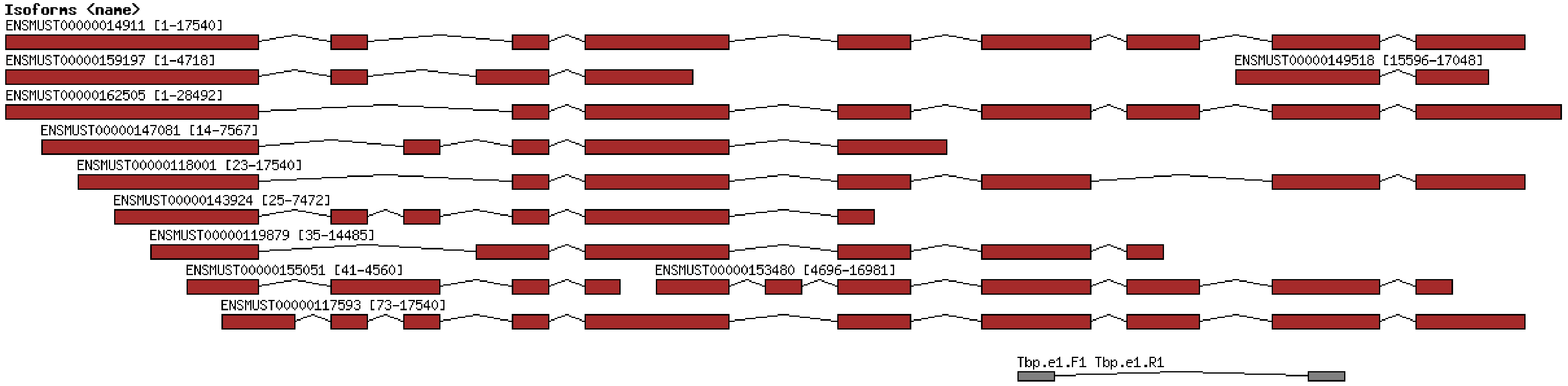

Figure S2 (cont'd)

Human ASE

ABI1

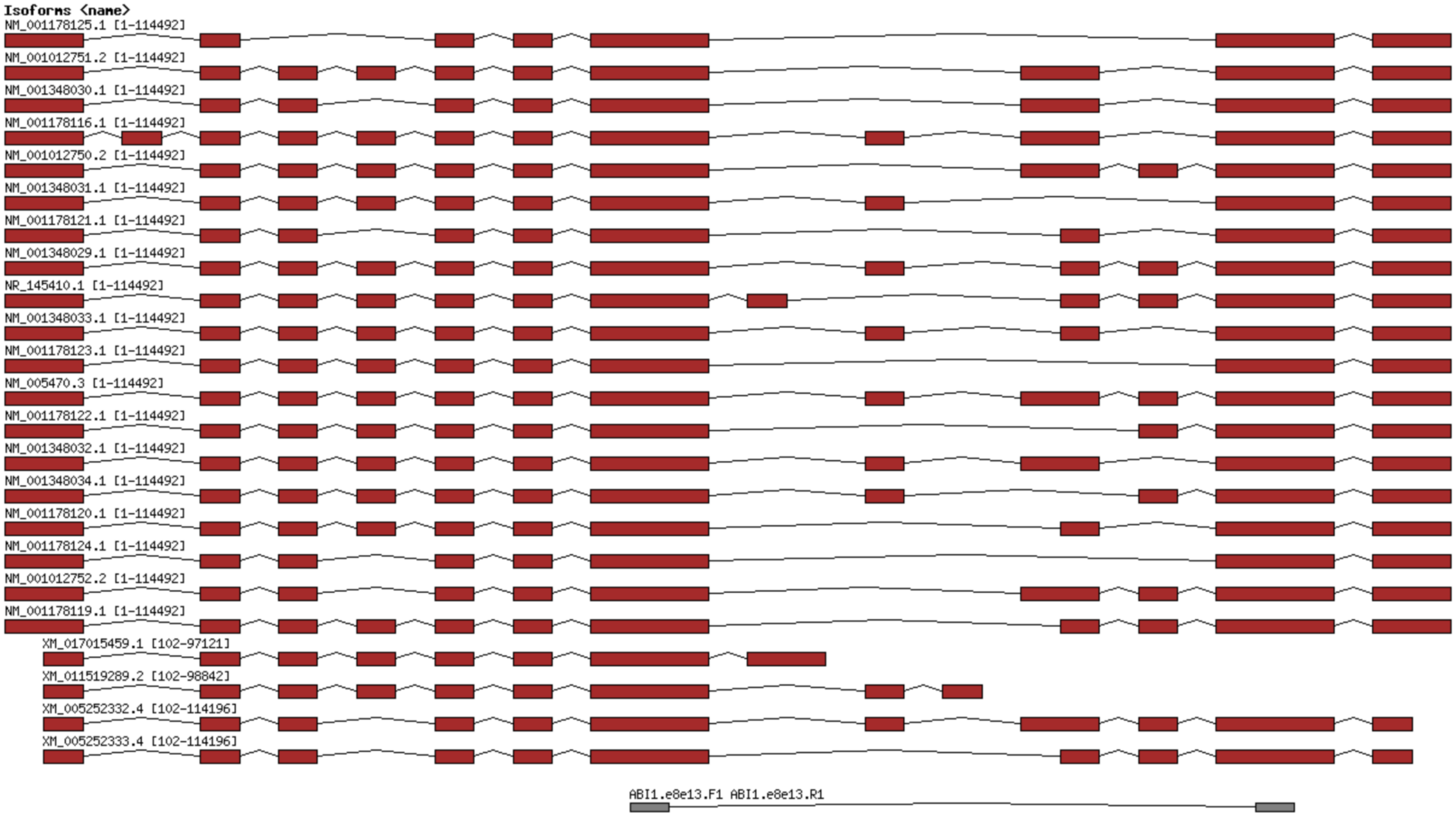

Figure S2 (cont'd)

CDKN2AIP

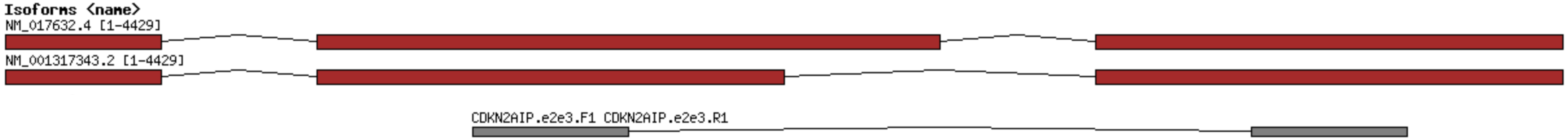

SERBP1

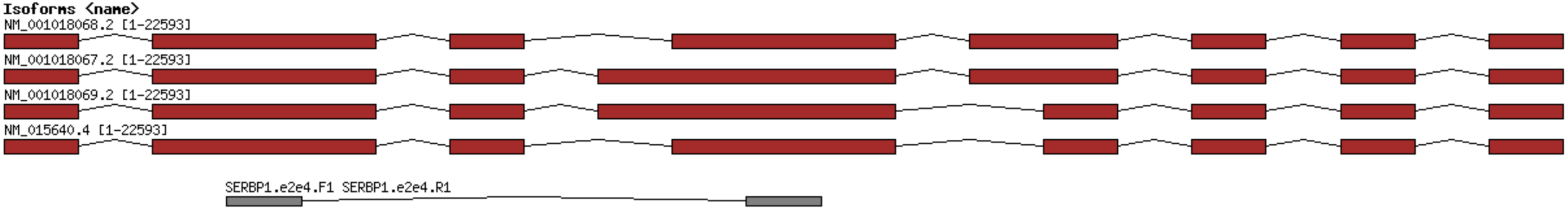

Figure S2 (cont'd)

Figure S3. Splicing profiles of the CDKN2AIP and EIF4A2 ASE in siCTRL and siRIG-I mock and infected L929 cells. siRNA were transfected using RNAiMAX, and 56 h post-transfection, cells were infected with MRV (T3D^{S}) at a MOI of 50, or mock-infected. Cells were further incubated for 16 h before RNA was harvested using Qiazol, reverse-transcribed, and subjected to AS-PCR for the CDKN2AIP and EIF4A2 ASE. PCR amplicons were resolved using capillary electrophoresis and quantified using relative fluorescence. n=3, biological replicates, unpaired two-tailed Student's t-test (ns, P>0.05; *, P≤0.05; **, P≤0.01; ***, P≤0.001; ****, P≤0.0001).

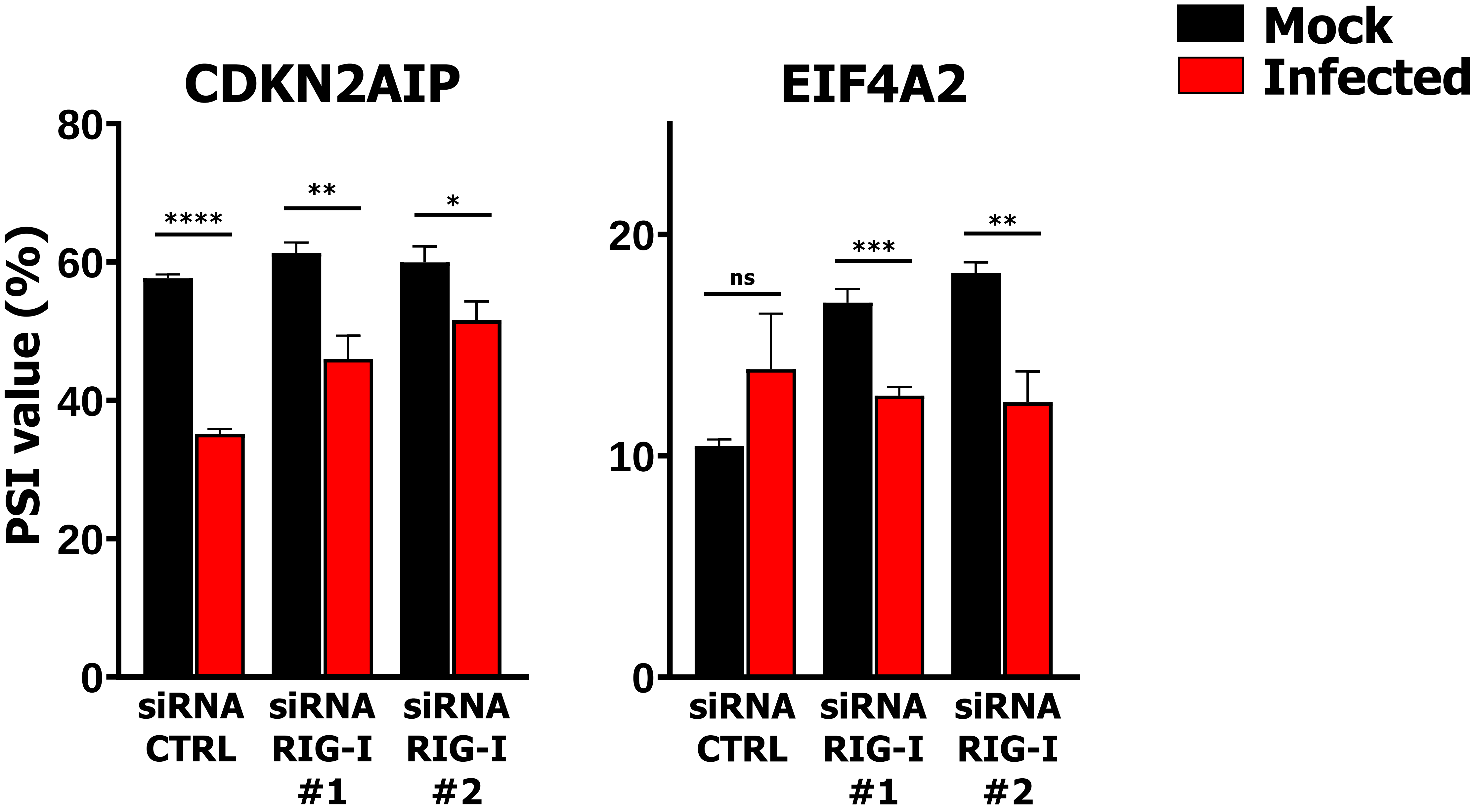
Mock
Infected
CDKN2AIP
EIF4A2
PSI value (%)
80
60
40
20
0
20
10
0
****
**
*
ns
***
**
siRNA CTRL
siRNA RIG-I #1
siRNA RIG-I #2
siRNA CTRL
siRNA RIG-I #1
siRNA RIG-I #2

Figure S3

Figure S4. Relative mRNA levels for S1 and M1 viral genes in siCTRL and siRIG-I mock and infected L929 cells. siRNA were transfected using RNAiMAX, and 56 h post-transfection, cells were infected with MRV ($T3D^{S}$) at a MOI of 50. Cells were further incubated for 16 h before RNA was harvested using Qiazol, reverse-transcribed, and subjected to qPCR for the S1 and M1 viral gene segment with PSMC4, PUM1, and TXNL4B used as housekeeping genes. The first replicate in the siCTRL condition was fixed at 1 and the relative mRNA expression was calculated for all other samples relative to that one. n=3, biological replicates, unpaired two-tailed Student's t-test (ns, $P>0.05$; **, $P\leq0.01$).

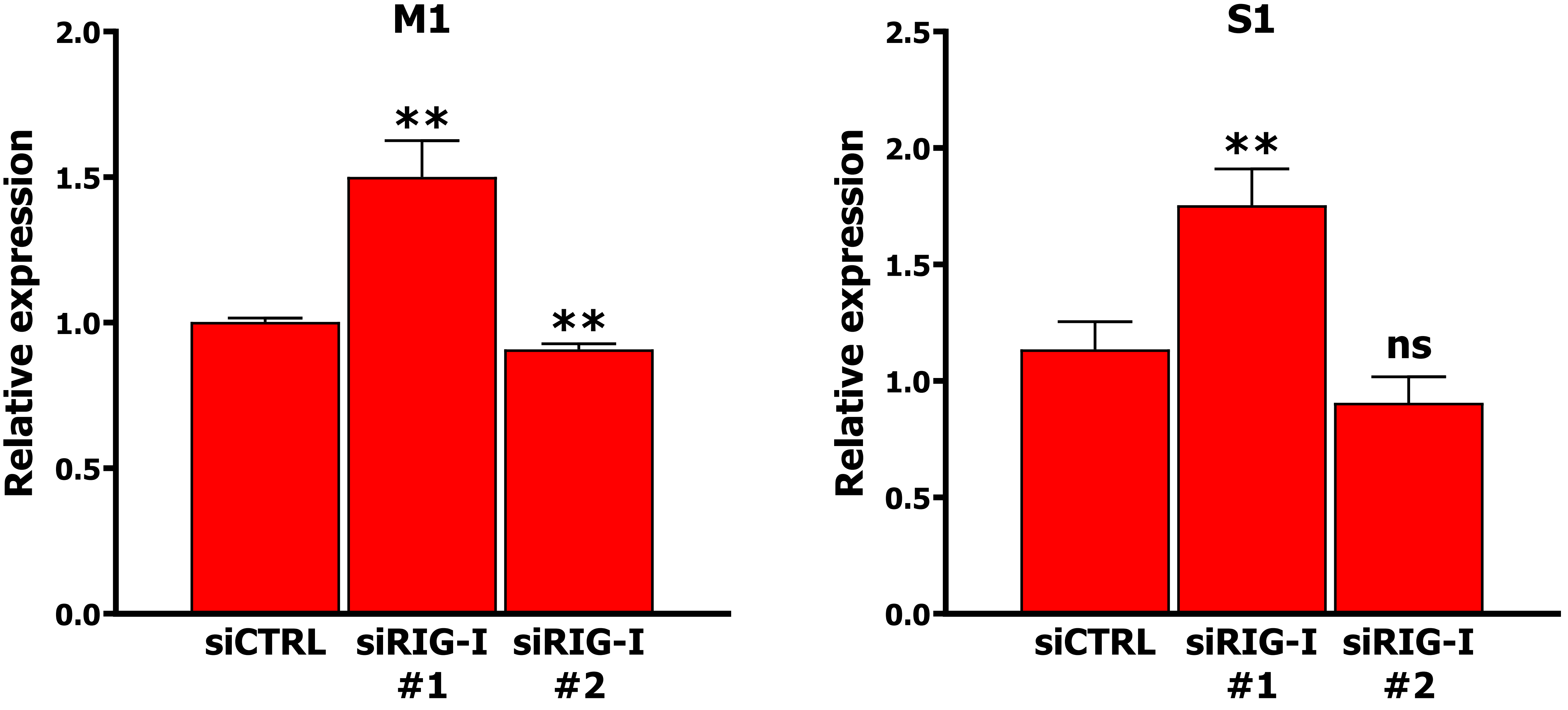
M1
Relative expression
2.0
1.5
1.0
0.5
0.0
**
**
siCTRL
siRIG-I #1
siRIG-I #2
S1
Relative expression
2.5
2.0
1.5
1.0
0.5
0.0
**
ns
siCTRL
siRIG-I #1
siRIG-I #2

Figure S4

Figure S5. Viral protein levels for σ3 and μ2 in siCTRL and siRIG-I infected L929 cells. siRNA were transfected using RNAiMAX, and 56 h post-transfection, cells were infected with MRV (T3D^{S}) at a MOI of 50. Cells were further incubated for 16 h before being lysed in RIPA. Protein lysates were dosed by Bradford assay, and western blots against σ3 and μ2 were realized. Membranes were H_2O_2-inactived and probed against the loading controls actin (μ2) and GAPDH (σ3). The relative protein level was calculated using Image J.

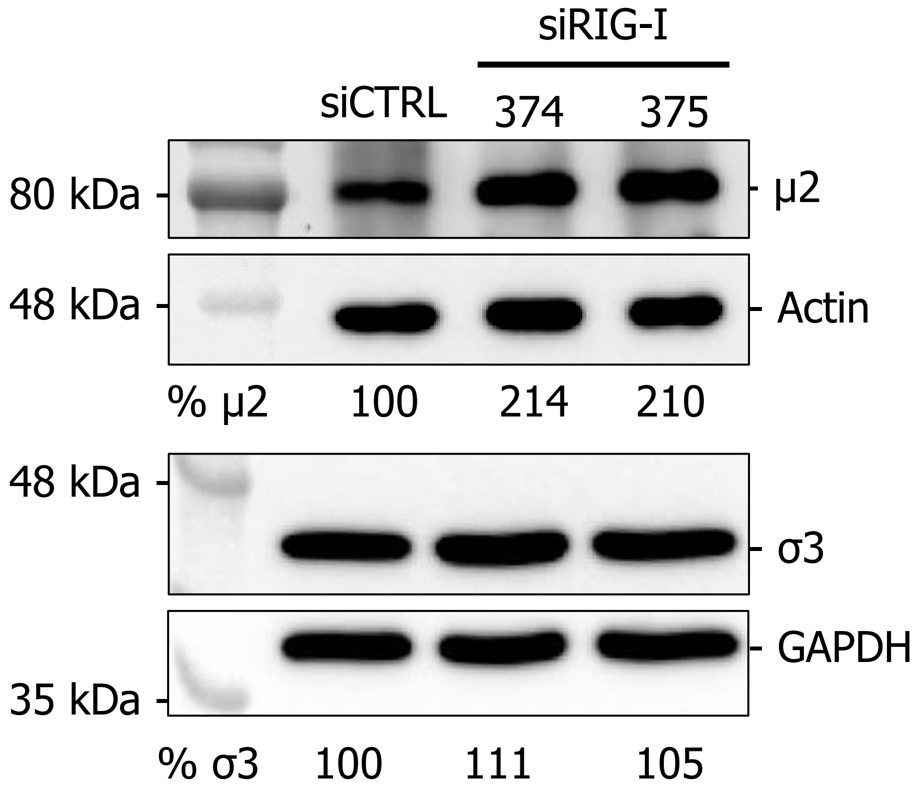
siRIG-I
siCTRL
374
375
80 kDa
μ2
48 kDa
Actin
% μ2
100
214
210
48 kDa
σ3
GAPDH
35 kDa
% σ3
100
111
105

Figure S5

Figure S6. Relative mRNA level for the S1 and M1 viral RNA and two ISG (DDX60 and MX1) by qPCR as controls for the bystander experiment. RNA was harvested at 16h post-infection from either the membrane (top, red) or the bystander (bottom, blue) cells when the top layer was infected or mock-infected with MRV ($T3D^S$) at a MOI of 50. PSMC4, PUM1, and TXNL4B were used as housekeeping genes for normalization. n=3, biological replicates, unpaired two-tailed Student's t-test comparing membrane (in red) and bystander (in blue; ns, $P>0.05$; *, $P\leq0.05$; **, $P\leq0.01$).

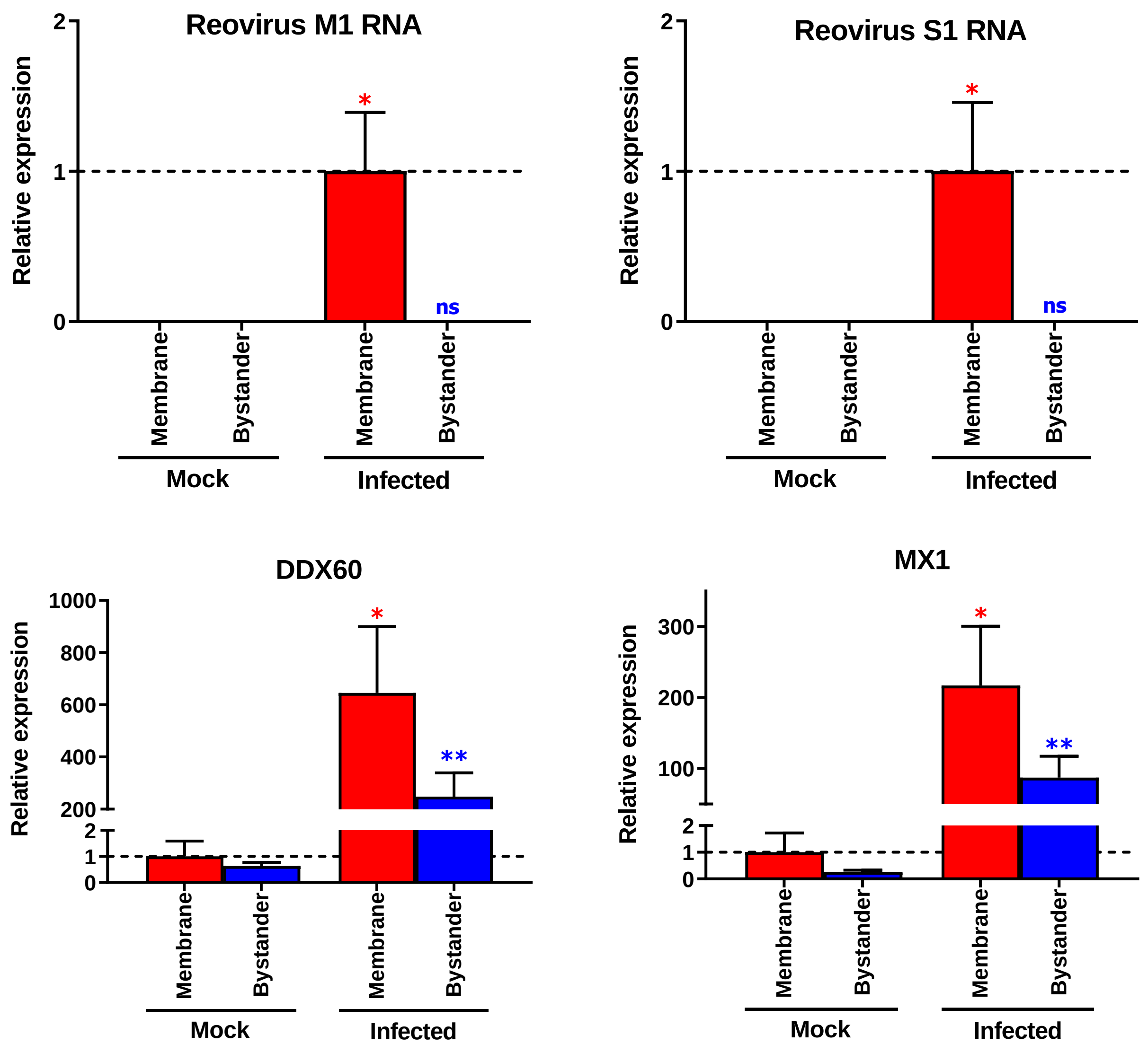

Reovirus M1 RNA
Relative expression
2
1
0
*
ns
Membrane
Bystander
Membrane
Bystander
Mock
Infected
Reovirus S1 RNA
Relative expression
2
1
0
*
ns
Membrane
Bystander
Membrane
Bystander
Mock
Infected
DDX60
Relative expression
1000
800
600
400
200
2
1
0
*
**
Membrane
Bystander
Membrane
Bystander
Mock
Infected
MX1
Relative expression
300
200
100
2
1
0
*
**
Membrane
Bystander
Membrane
Bystander
Mock
Infected

Figure S6

Figure S7. Splicing profiles of the CDKN2AIP and EIF4A2 ASE in the bystander experiment. RNA was harvested at 16 h post-infection from either the membrane (red) or the bystander (blue) cells when the top layer was infected or mock-infected with MRV ($T3D^S$) at a MOI of 50. n=3, biological replicates, unpaired two-tailed Student's t-test (ns, $P>0.05$; *, $P\leq0.05$; **, $P\leq0.01$; ***, $P\leq0.001$; ****, $P\leq0.0001$).

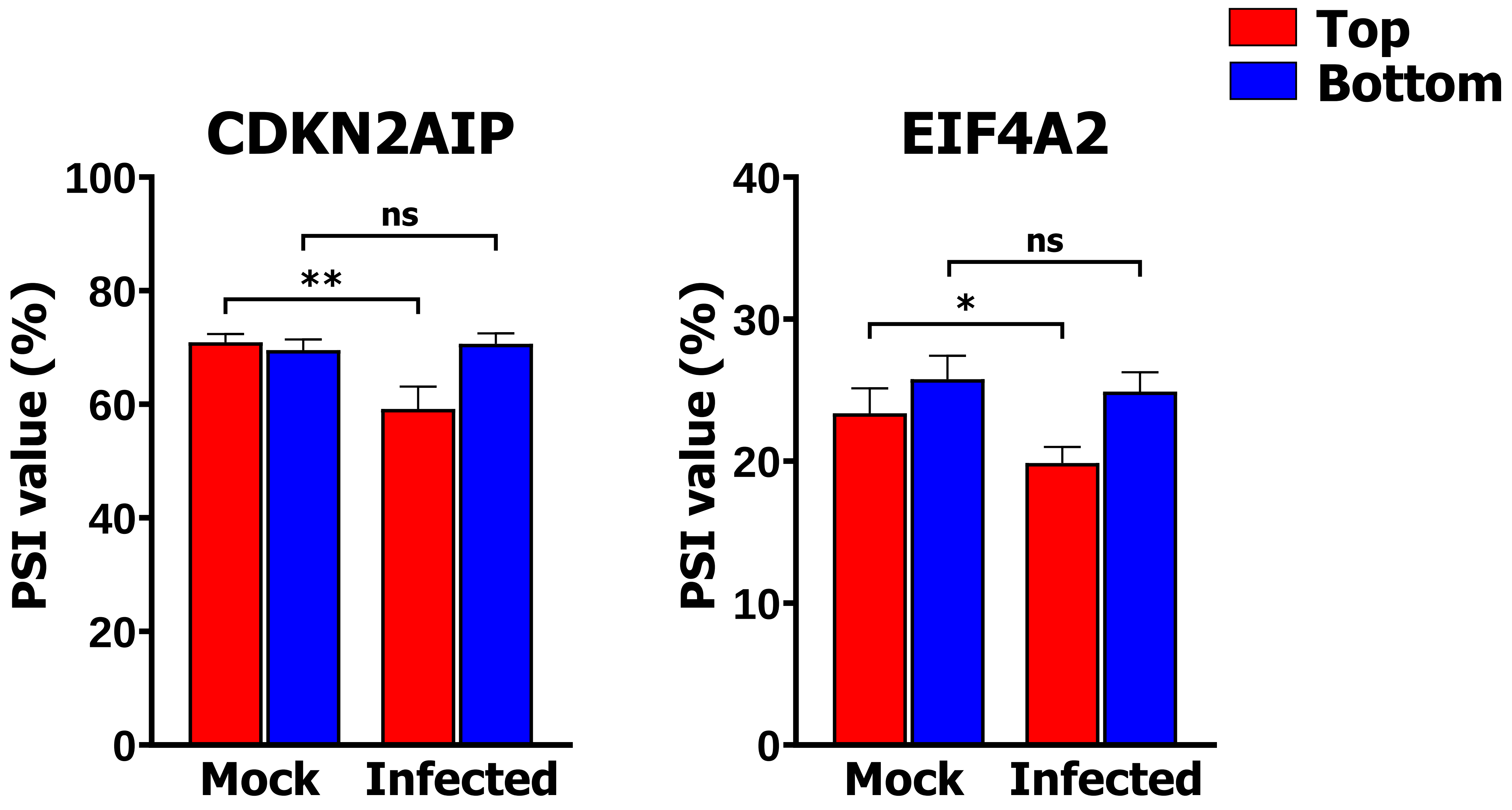

Top
Bottom
CDKN2AIP
PSI value (%)
100
80
60
40
20
0
ns
**
Mock
Infected
EIF4A2
PSI value (%)
40
30
20
10
0
ns
*
Mock
Infected

Figure S7

Figure S8. Impact of IFN-β treatment on the studied ASE.

L929 cells were plated at 2.5 x 10^5 cells per well in 12-well plates, and treated the following morning with 10, 100 or 1,000 U/mL of recombinant mouse interferon beta (PBL Assay Science, #12401-1) for 5 h. RNA was extracted using Qiazol, reverse-transcribed, and subjected to AS-PCR for the different ASE analyzed. PCR amplicons were resolved using capillary electrophoresis and quantified using relative fluorescence. All treated results were not statistically different from the untreated condition using an unpaired two-tailed Student's t-test. n=3, biological replicates.

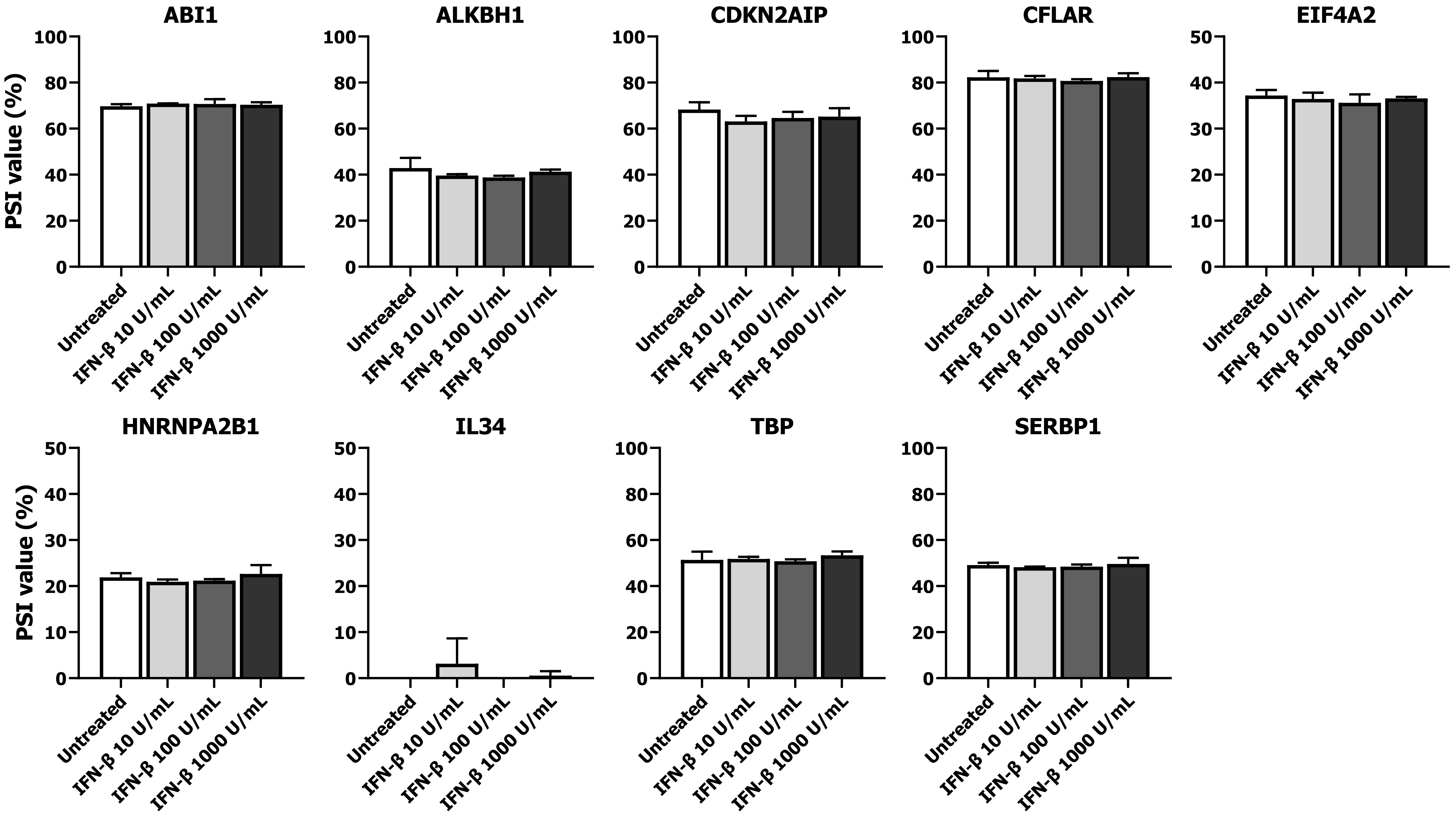
ABI1
ALKBH1
CDKN2AIP
CFLAR
EIF4A2
HNRNPA2B1
IL34
TBP
SERBP1
PSI value (%)
Untreated
IFN-β 10 U/mL
IFN-β 100 U/mL
IFN-β 1000 U/mL

Figure S8

Figure S9. Splicing profiles of additional ASE modulated by reovirus throughout infection.

L929 cells were infected with T3D[s] at a MOI of 50, RNA was extracted at indicated time point using Qiazol, reverse-transcribed, and subjected to AS-PCR for the different ASE analyzed. PCR amplicons were resolved using capillary electrophoresis and quantified using relative fluorescence. n=3, biological replicates, one-way ANOVA with Dunnett's multiple comparisons test against the 0 h mock condition (ns, $P>0.05$; *, $P\leq0.05$; **, $P\leq0.01$; ***, $P\leq0.001$; ****, $P\leq0.0001$).

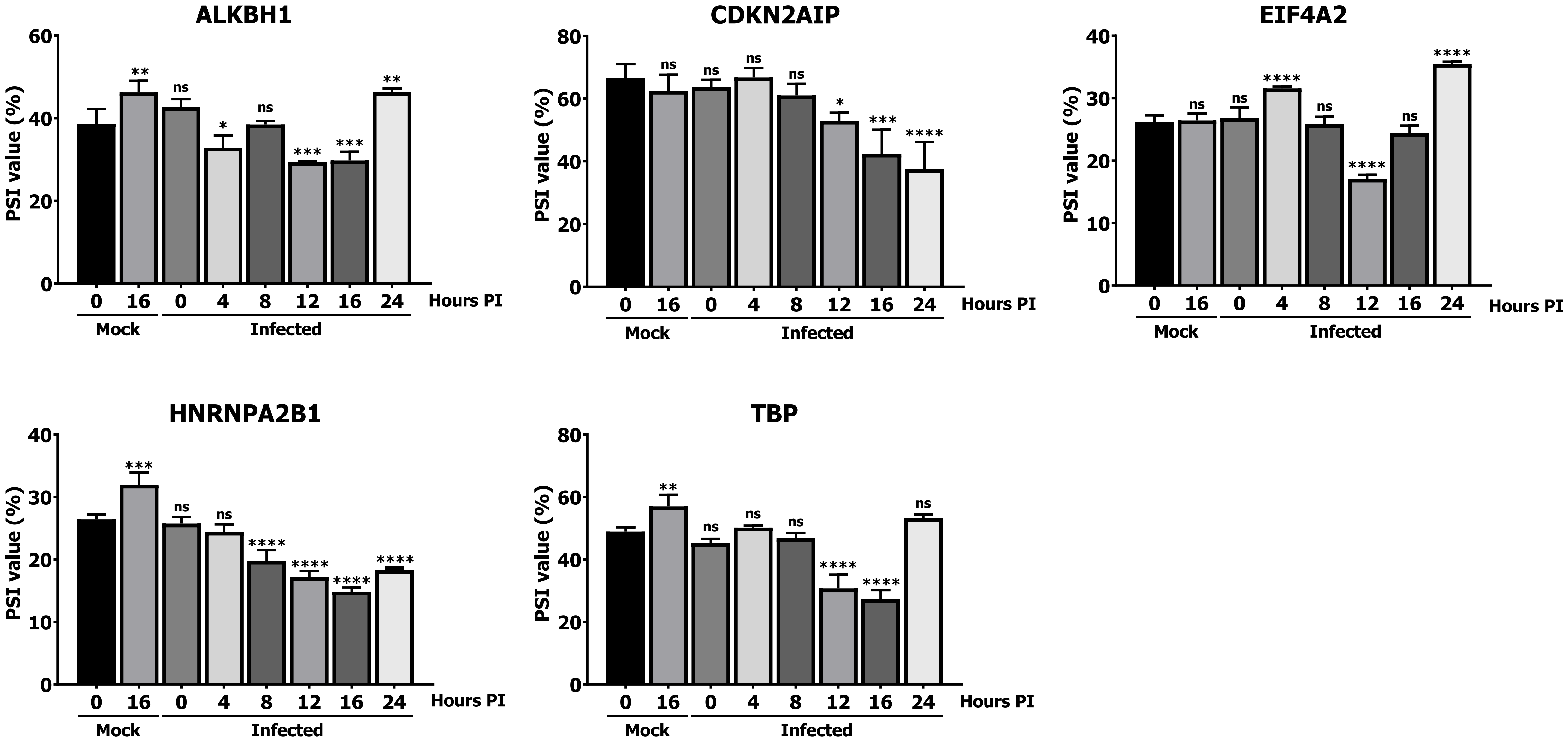

ALKBH1
CDKN2AIP
EIF4A2
HNRNPA2B1
TBP
PSI value (%)
Hours PI
0 16 0 4 8 12 16 24
Mock
Infected
ns
*
**
***
****

Figure S9

Figure S10. Splicing profiles of the ALKBH1, CDKN2AIP, and EIF4A2 ASE upon infection with all *wild-type*/reassortant/single amino acid mutant MRV viruses. Single amino acid mutants, M1T3D^K[T3D^S], and M1T3D^S[T3D^K] reassortant viruses were rescued by reverse genetics. L929 cells were infected with respective reovirus at a MOI of 50, or mock-infected. RNA was extracted at 16 h PI using Qiazol, reverse-transcribed, and subjected to AS-PCR for the different ASE analyzed. PCR amplicons were resolved using capillary electrophoresis and quantified using relative fluorescence. n=3, biological replicates, one-way ANOVA with Dunnett's multiple comparisons test against the mock condition for T3D^S/T3D^K (in black); two-way ANOVA with Šídák's multiple comparisons test against the parental virus (T3D^S in red and T3D^K in green) for the reassortant and mutant viruses (ns, P>0.05; *, P≤0.05; **, P≤0.01; ***, P≤0.001; ****, P≤0.0001).

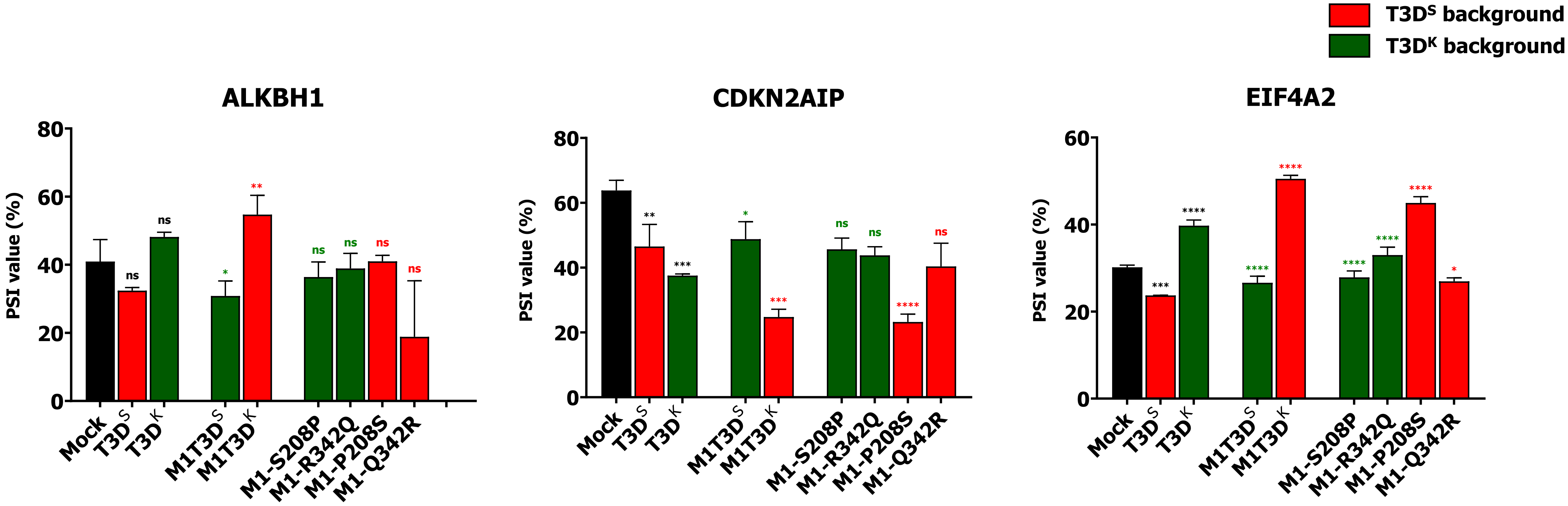
T3D^S background
T3D^K background
ALKBH1
CDKN2AIP
EIF4A2
PSI value (%)
Mock
T3D^S
T3D^K
M1T3D^S
M1T3D^K
M1-S208P
M1-R342Q
M1-P208S
M1-Q342R
ns
*
**
***
****

Figure S10

Figure S11. Relative mRNA levels of S1 and M1 viral genes in all *wild-type*/reassortant/single amino acid mutant MRV viruses. L929 cells were infected with the respective virus at a MOI of 50, RNA was extracted at 16 h PI using Qiazol, reverse-transcribed, and subjected to qPCR for either the M1 or S1 RNA with PSMC4, PUM1, and TXNL4B as housekeeping genes for normalization. Results were normalized against $T3D^S$, to allow for an easier comparison of RNA levels between the different viruses. n=3, biological replicates, two-way anova with Tukey's multiple comparisons test where all groups were compared; results of the comparison against $T3D^S$ are shown (ns, $P>0.05$; *, $P\leq0.05$; ***, $P\leq0.001$; ****, $P\leq0.0001$).

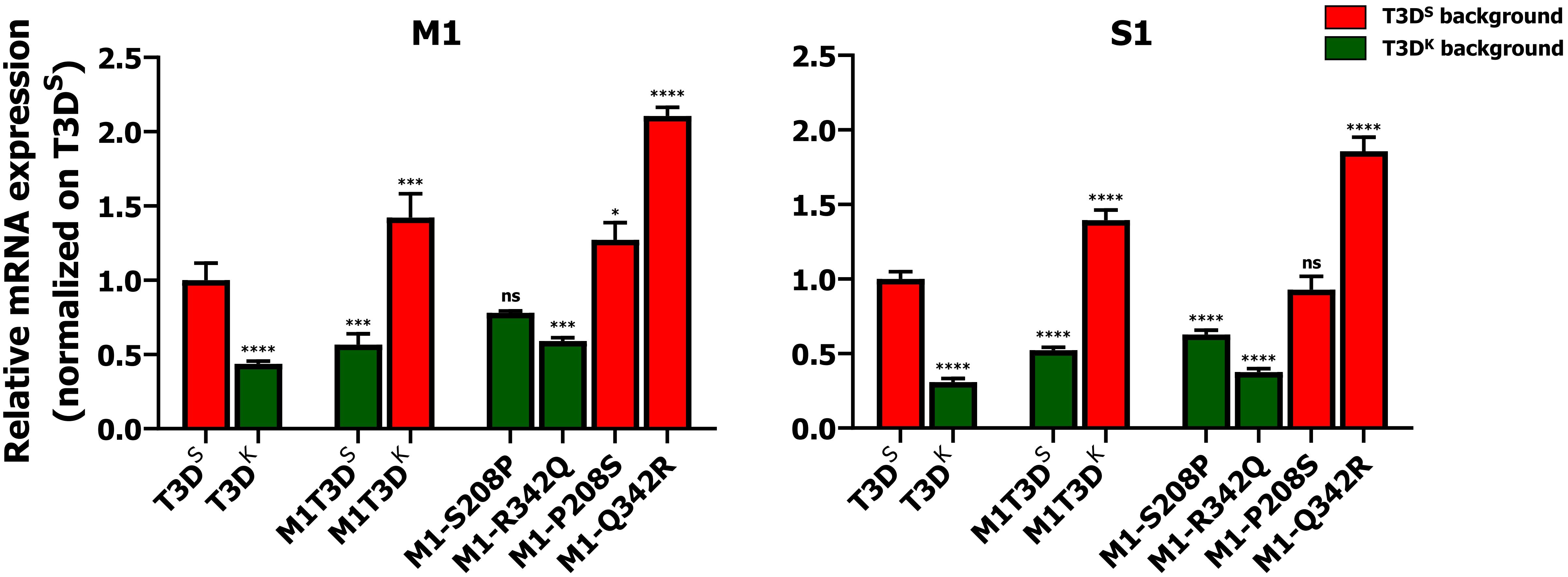
M1
S1
Relative mRNA expression
(normalized on T3DS)
T3DS background
T3DK background
2.5
2.0
1.5
1.0
0.5
0.0
****
***
***
ns
***
*
****
****
****
****
****
****
ns
****
T3DS
T3DK
M1T3DS
M1T3DK
M1-S208P
M1-R342Q
M1-P208S
M1-Q342R

Figure S11

Figure S12. Viral protein levels of σ3 in all *wild-type*/reassortant/single amino acid mutant MRV viruses. L929 cells were infected with the respective virus at a MOI of 50 and incubated for 16 h before being lysed in RIPA. Protein lysates were dosed by Bradford assay and a western blot against σ3 was realized. The membrane was H_2O_2-inactived and probed against the loading control GAPDH. Relative protein level was calculated using Image J.

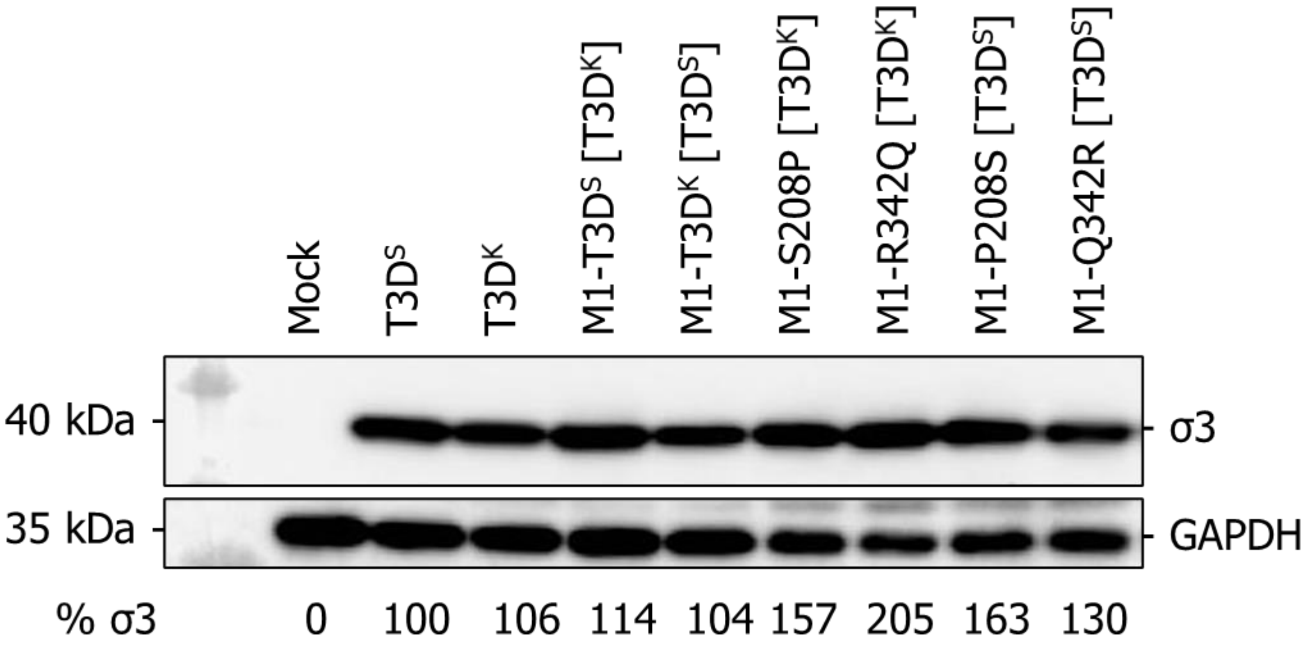
Mock
T3D^S
T3D^K
M1-T3D^S [T3D^K]
M1-T3D^K [T3D^S]
M1-S208P [T3D^K]
M1-R342Q [T3D^K]
M1-P208S [T3D^S]
M1-Q342R [T3D^S]
40 kDa
35 kDa
σ3
GAPDH
% σ3
0
100
106
114
104
157
205
163
130

Figure S12

Figure S13. Live cell imaging of GFP alone or GFP-tagged μ2 in SV40 large T antigen harboring 293T and COS-7 cells. Cells were transfected with either Lipofectamine2000 (293T cells) or LipofectamineLTX (COS-7) following the manufacturer's protocol and incubated for 24 h. Cells were then directly imaged using a Nikon TE2000E epifluorescence microscope at 488 nm, 4x objective and a 500 ms exposure time. The scale bars represent 100 μm.

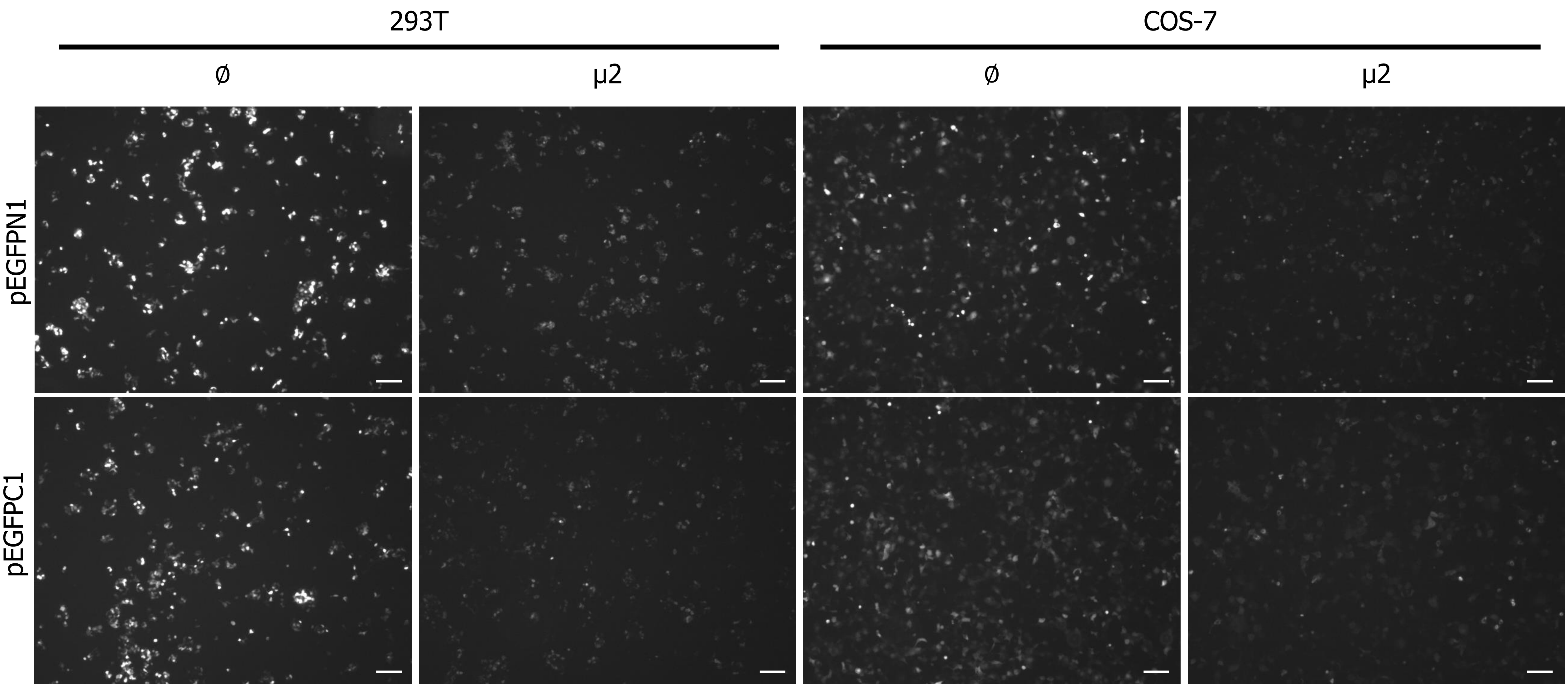

Figure S13

Figure S14. Quantification of the nuclear signal for the different µ2 constructions.

(A) Percent of COS-7 cells with clear nuclear signal over all cells presenting the expression of the µ2 constructs. Cells were transfected for 24 h, and at least 10 fields for an average of 100 cells were quantified using epifluorescence microscopy. Fields were randomly separated into three groups to form three independent quantification replicates. Two-way ANOVA with Dunnett's multiple comparisons test against µ2-GFP (in green) or GFP-µ2 (in red) (**, $P \leq 0.01$; ***, $P \leq 0.001$; ****, $P \leq 0.0001$). **(B)** The histograms display the fluorescence intensity along the red arrow in the panels from Figure 4A and 4D. Scale bar, 5 µm.

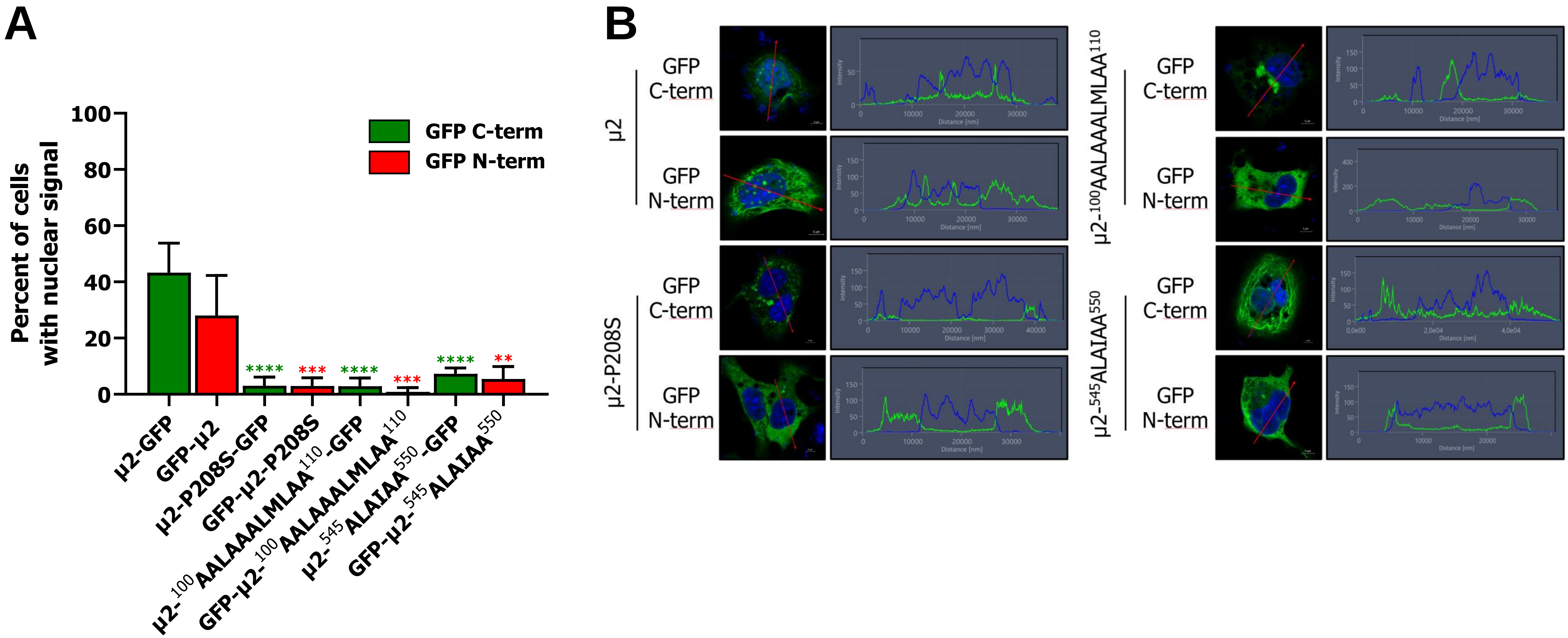

A
Percent of cells with nuclear signal
100
80
60
40
20
0
GFP C-term
GFP N-term
****
***
****
***
****
**
μ2-GFP
GFP-μ2
μ2-P208S-GFP
GFP-μ2-P208S
μ2-100AALAAALMLAA110-GFP
GFP-μ2-100AALAAALMLAA110
μ2-545ALAIAA550-GFP
GFP-μ2-545ALAIAA550
B
μ2
μ2-P208S
μ2-100AALAAALMLAA110
μ2-545ALAIAA550
GFP C-term
GFP N-term

Figure S14

Figure S15. Cellular localization of GFP-μ2 constructs in 293T cells.

293T cells were transfected with GFP-tagged μ2 or μ2-P208S using Lipofectamine2000, and directly imaged using epifluorescence microscopy 24 h after transfection. A Nikon TE2000E epifluorescence microscope was used with a 20X objective at 488 nm and a 100 ms exposure time. Ø denotes an empty control plasmid encoding only the GFP moiety. The scale bars represent 20 μm.

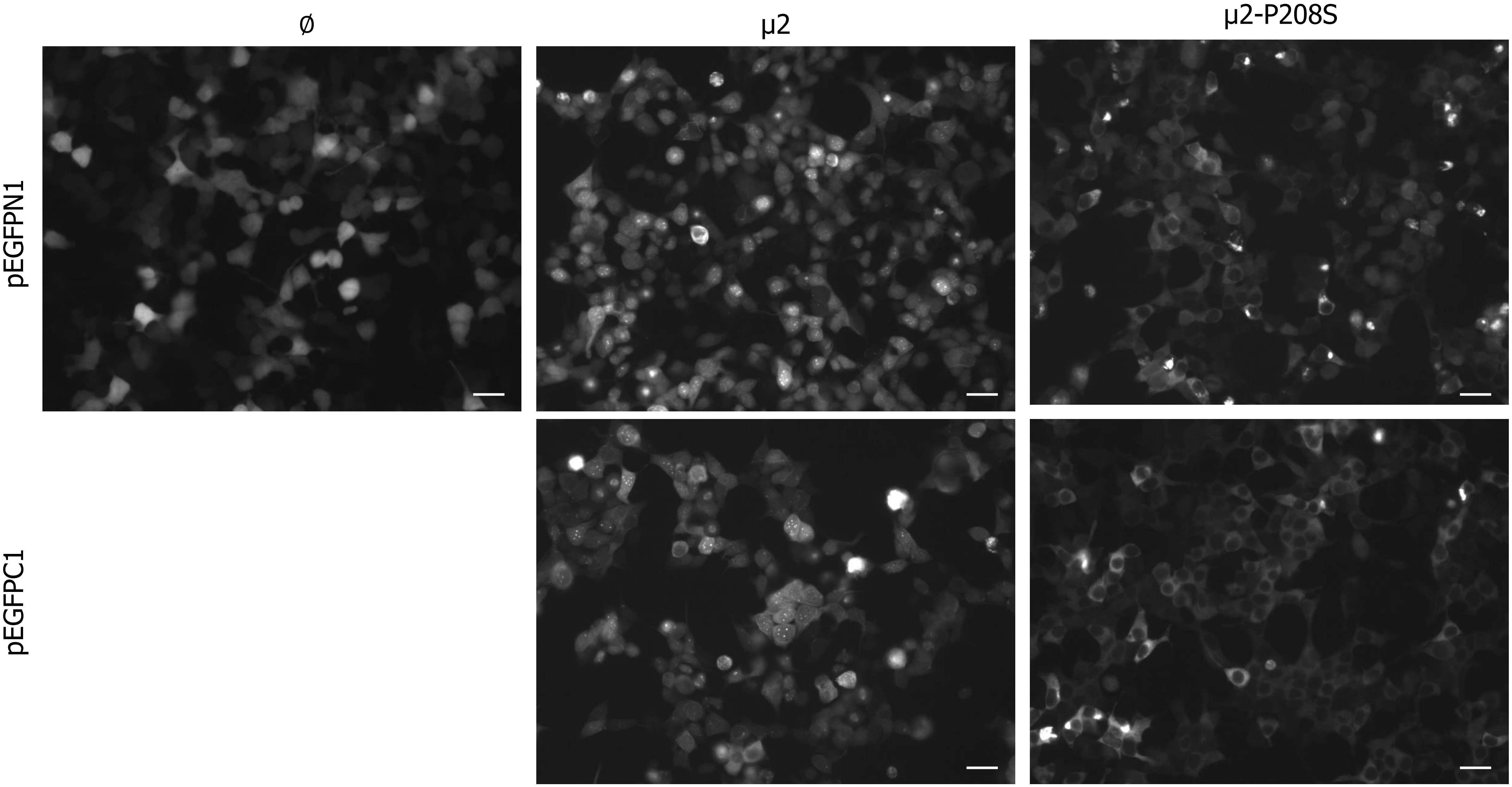

Figure S15

Figure S16. Western blot validation of the expression of different µ2-GFP constructs. 293T cells were transfected with Lipofectamine2000, incubated for 24 h before being lysed in RIPA. Soluble protein lysates were dosed by Bradford assay, and a western blot against GFP was realized. The membrane was H_2O_2-inactived and probed against the loading control GAPDH.

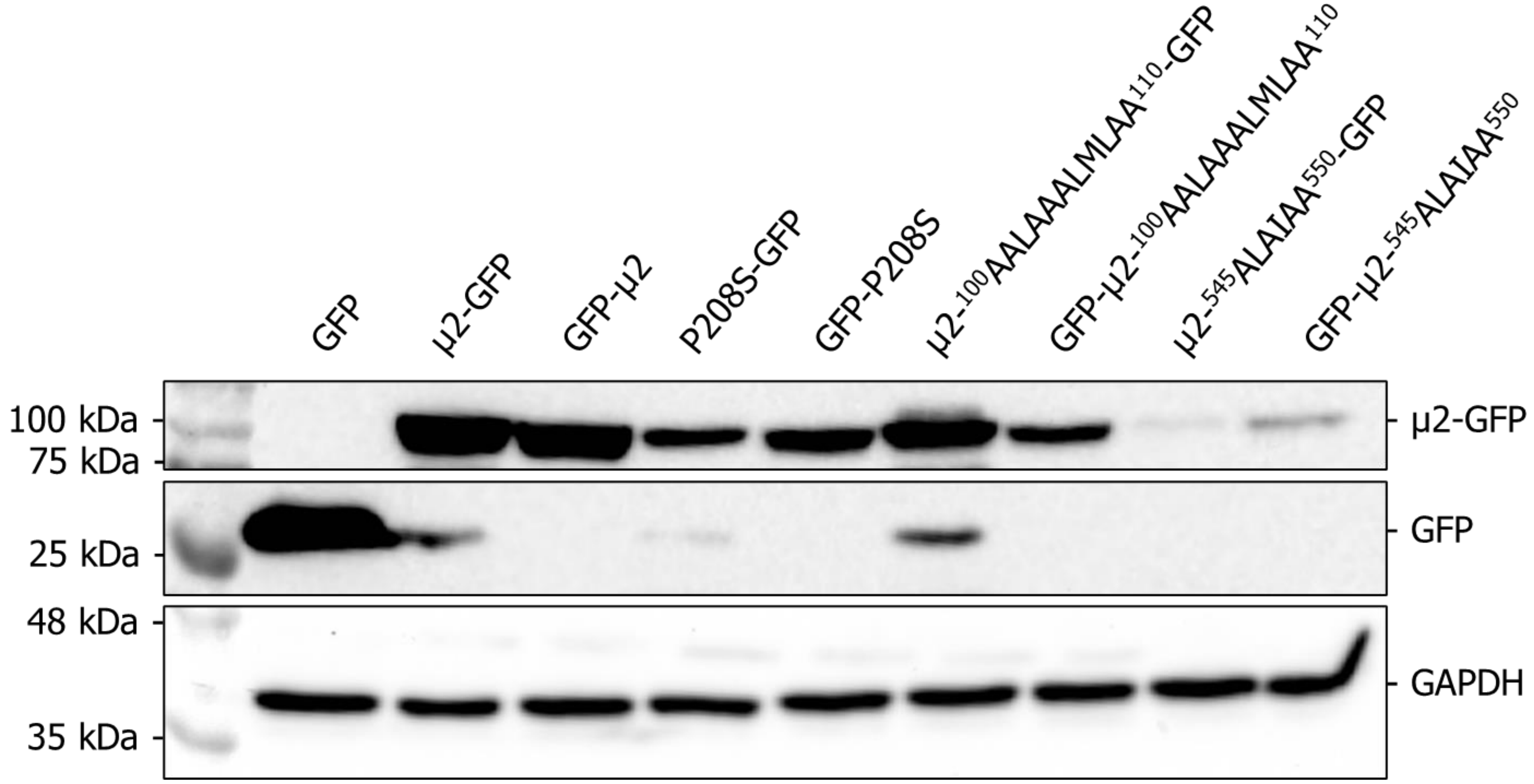
GFP
μ2-GFP
GFP-μ2
P208S-GFP
GFP-P208S
μ2-100AALAAALMLAA110-GFP
GFP-μ2-100AALAAALMLAA110
μ2-545ALAIAA550-GFP
GFP-μ2-545ALAIAA550
100 kDa
75 kDa
25 kDa
48 kDa
35 kDa
μ2-GFP
GFP
GAPDH

Figure S16

Figure S17. Epifluorescence validation of the expression of different μ2-GFP constructs. 293T cells were transfected with Lipofectamine2000, incubated for 24 h and directly imaged using epifluorescence microscopy. A Nikon TE2000E epifluorescence microscope was used with a 4X objective at 488 nm and a 500 ms exposure time. ∅ denotes an empty control plasmid encoding only the GFP moiety. The scale bars represent 100 μm.

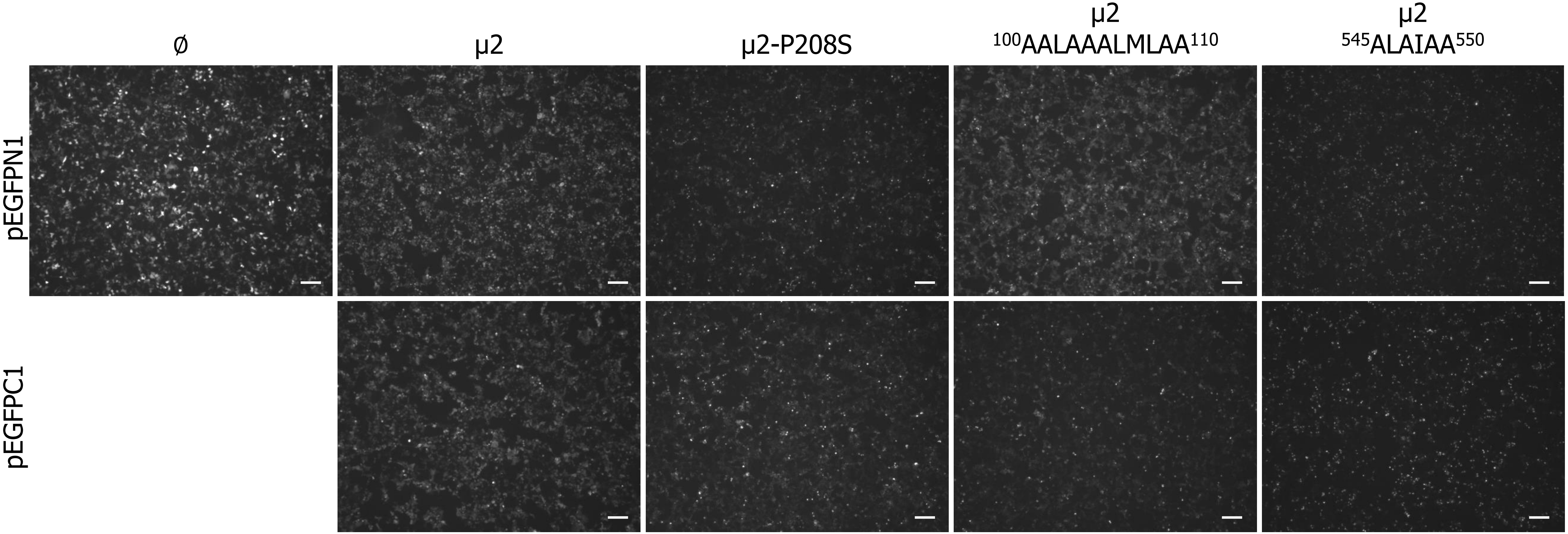

Ø
μ2
μ2-P208S
μ2
100AALAAALMLAA110
μ2
545ALAIAA550
pEGFPN1
pEGFPC1

Figure S17

Figure S18. Splicing profiles of endogenous human ASE in the *ABI1*, *CDKN2AIP*, and *SERBP1* upon expression of different μ2-GFP constructs. 293T cells were transfected with Lipofectamine2000, incubated for 24 h and RNA was extracted using Qiazol, reverse-transcribed, and subjected to AS-PCR for the different ASE analyzed. PCR amplicons were resolved using capillary electrophoresis and quantified using relative fluorescence. n=3, biological replicates, unpaired two-tailed Student's t-test (ns, $P>0.05$; *, $P\leq0.05$; **, $P\leq0.01$; ***, $P\leq0.001$; ****, $P\leq0.0001$) against the GFP alone condition.

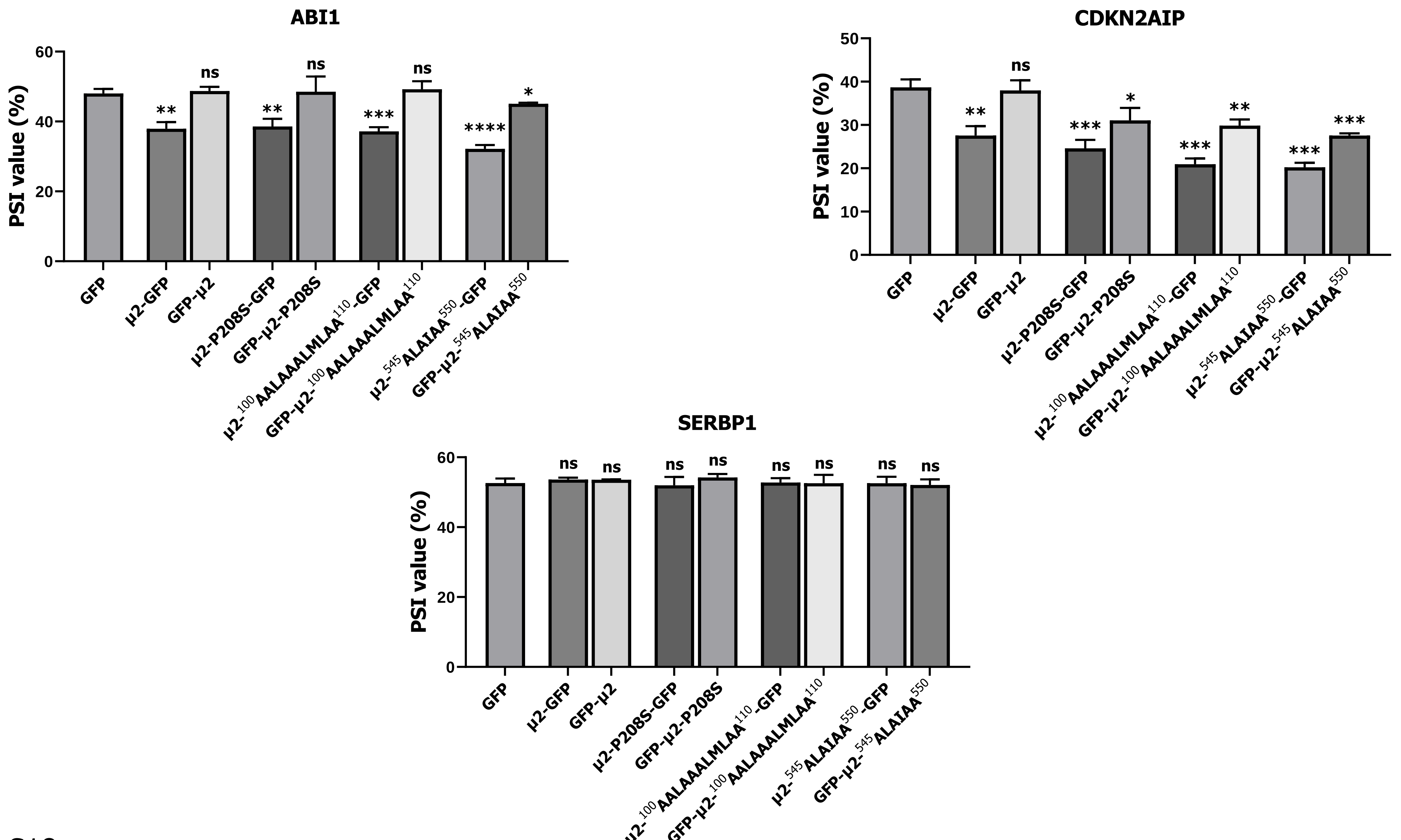
ABI1
CDKN2AIP
SERBP1
PSI value (%)
GFP
μ2-GFP
GFP-μ2
μ2-P208S-GFP
GFP-μ2-P208S
μ2-100AALAAALMLAA110-GFP
GFP-μ2-100AALAAALMLAA110
μ2-545ALAIAA550-GFP
GFP-μ2-545ALAIAA550
ns
**
***
****
*

Figure S18

Figure S19. Sequence of the T3D^S μ2 protein and relevant potential NLS involved in the nuclear localization. Potential NLS are underlined and basic residues are shown bold. The P208 position is indicated in red.

MAYIAVPAVVDSRSSEAIGLLESFGVDAGADANDVSYQDHDYVLDQLQYMLDGYEAGDVI
DALVHKNWLHHSVYCLLPPKSQLLEYWKSNPSAIPDNVD**RR**L**RKR**LML**KK**DLRKDDEYNQ
LARAFKISDVYAPLISSTTSPMTMIQNLNQGEIVYTTTDRVIGARILLYAPRKYYASTLS
FTMTKCIIPFGKEVGRVPHSRFNVGTFPSIATPKCFVMSGVDIESIPNEFIKLFYQRVKS
VHANILNDISPQIVSDMINRKRLRVHTPSDRRAAQLMHLPYHVKRGASHVDVYKVDVVDM
LFEVVDVADGLRNVSRKLTMHTVPVCILEMLGIEIADYCIRQEDGMLTDWFLLLTMLSDG
LTDRRTHCQYLINPSSVPPDVILNISITGFINRHTIDVMPDIYDFVKPIGAVLPKGSFKS
TIMRVLDSISILGIQIMPRAHVVDSDEVGEQMEPTFEQAVMEIYKGIAGVDSLDDLIKWV
LNSDLIPHDDRLGQLFQAFLPLAKDLLAPMARKFYDNSMSEGRLLTFAHADSELLNANYF
GHLL**R**L**K**I**PY**ITEVNLMIRKNREGGELFQLVLSYLYKMYATSAQPKWFGSLLRLLICPWL
HMEKLIGEADPASTSAEIGWHIPREQLMQDGWCGCEDGFIPYVSIRAPRLVIEELMEKNW
GQYHAQVIVTDQLVVGEPRRVSAKAVIKGNHLPVKLVSRFACFTLTAKYEMRLSCGHSTG
RGAAYSARLAFRSDLA

Figure S19

Figure S20. Individual SAINT scores for each IP-MS replicates.

Each independent replicate was analyzed using SAINT against the corresponding control GFP IP. Identified proteins (x-axis) are sorted in increasing SAINT score (y-axis). SAINT scores above 0.9 were considered a hit; the number of hits for each independent replicates are depicted.

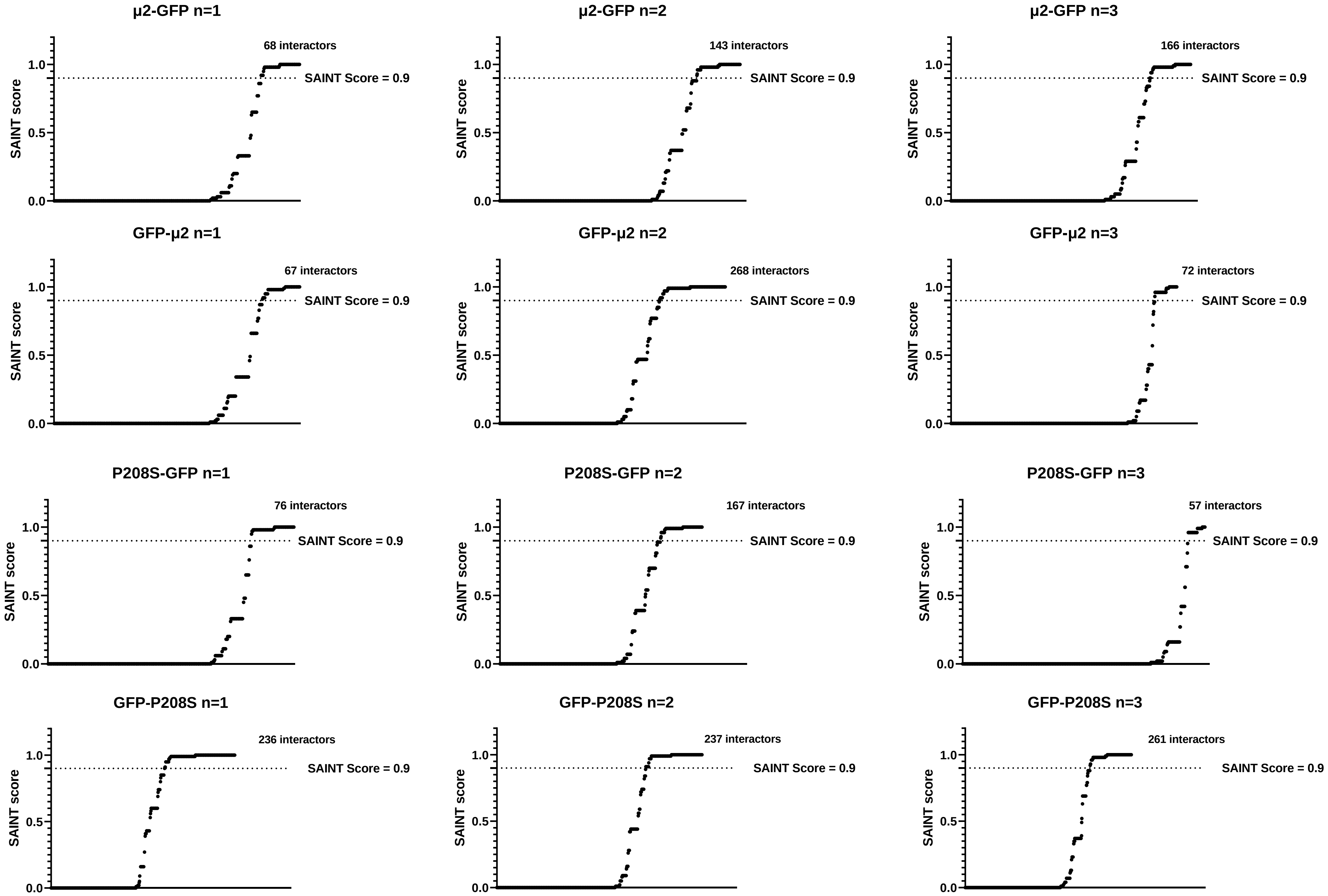
μ2-GFP n=1
68 interactors
μ2-GFP n=2
143 interactors
μ2-GFP n=3
166 interactors
GFP-μ2 n=1
67 interactors
GFP-μ2 n=2
268 interactors
GFP-μ2 n=3
72 interactors
P208S-GFP n=1
76 interactors
P208S-GFP n=2
167 interactors
P208S-GFP n=3
57 interactors
GFP-P208S n=1
236 interactors
GFP-P208S n=2
237 interactors
GFP-P208S n=3
261 interactors
SAINT Score = 0.9
SAINT score
0.0
0.5
1.0

Figure S20

Figure S21. Complete list of identified cellular partners for μ2 and μ2-P208S using IP-MS. On the left, the respective lists of identified cellular partners by alphabetical order are depicted for each construction tested. On the right, the lists correspond to the overlaps presented in Figure 5B, which were proteins identified with more than one construction.

eGFP-μ2 (67)

ADNP
ATAD3B
BAG2
BCLAF1
C14ORF166
CBX3
CCAR2
CCDC6
CCNT1
CCT2
CCT4
CCT8
CDK9
CEP170
CKAP5
CLASP2
COPA
CSNK2A1
DDX21
DHX30
EFTUD2
EXOSC10
FAM120A
FAM98B
FTSJ3
GTF3C5
GTSE1
HADHA
HELZ
HERC2
HSP90AA1
IRS4
LAS1L
MARK2
MRPS12
MRPS25
MYCBP2
NEK1
NEURL4
NOC3L
NOL9
NOP2
NUP153
PABPC1
PARD3
PHLDB2
PKP4
PNN
POLDIP3
PPP1CC
PRPF8
RANBP2
RPF2
RPL10A
RPL31
RPL5
RPL9
RSL1D1
SLAIN1
SNRNP200
SON
SRRM2
SUGP2
TANC2
VIM
WDR6
XRCC6

μ2-eGFP (77)

ABCD3
ADNP
AKAP8
BAG2
CAMK2B
CAMK2D
CAMK2G
CCAR2
CCNT1
CCT2
CCT4
CCT5
CCT7
CCT8
CDK9
CEP170
CHD4
CHTOP
CKAP5
CLASP2
COPA
COPB2
CYC1
CYLD
DDX1
DDX21
DHX30
DYNC1H1
EFTUD2
EIF3C
EMD
FAM120A
GTF3C5
HELZ
HERC2
IKBKAP
LAS1L
LGALS3BP
MAGED1
MLF2
MYBBP1A
MYCBP2
MYO1C
NEK1
NIPSNAP1
NOLC1
NUP133
NUP153
NUP205
PABPC1
PGAM5
PHLDB2
PKP2
POLDIP3
POLR2B
PRKDC
PRPF8
RANBP2
RPL10A
RPL12
RPL7A
RPL9
RSL1D1
RUVBL2
SEC24B
SKIV2L2
SLAIN1
SNRNP200
SON
SPATA2
UPF1
UQCRH
USP9X
VIM
WDR6
XRN2
ZMYM3

eGFP-μ2-P208S (195)

ABCD3
ADNP
AIFM1
AIMP2
AKAP8
AKAP8L
AMBRA1
ATAD3A
ATAD3B
BAG2
CBX3
CCAR2
CCT2
CCT3
CCT4
CCT5
CCT6A
CCT7
CCT8
CDC42EP1
CEP170
CHD4
CKAP5
COPA
COPB1
COPB2
COPE
CPNE3
CSNK2A1
CSNK2B
CYC1
DDX1
DDX21
DDX28
DHX30
DPM1
DPY30
DYNC1H1
DYNLL1
EFTUD2
EIF2S3
EIF3A
EIF3B
EIF3C
EIF3E
EIF3G
EIF4A1
EMD
EPRS
ERLIN2
EXOSC10
FAF2
FAM120A
FAM98A
FAM98B
FAR1
FLII
G3BP1
G3BP2
GAK
GALK1
GANAB
GATAD2A
GCN1L1
GLUD1
GPRASP2
GTF2I
GTF3C5
GTSE1
HACD3
HADHA
HAX1
HDAC2
HELZ
HERC2
HNRNPC
HNRNPR
HSP90AA1
HSPH1
IARS
IKBKAP
IMMT
IRS4
KPNA1
KPNA4
LARP4B
LARS
LAS1L
LBR
LMNB1
MAGED1
MARS
MATR3
MDN1
MLF2
MRPL37
MRPS22
MRPS25
MSH6
MTA2
MYBBP1A
MYCBP2
MYH9
MYO1B
MYO1C
NDUFA9
NEK1
NEURL4
NKRF
NOC2L
NPM3
NUDT21
NUP107
NUP133
NUP153
NUP160
NUP205
NUP214
NUP93
P4HB
PABPC1
PELO
PGAM5
PHB
PHB2
PKP4
POLDIP3
POLR2A
POLR2B
PRKDC
PRMT5
PRPF19
PRPF6
PRPF8
PSMC2
QARS
RANBP2
RARS
RBBP7
RCN2
RFC5
RPL10A
RPL28
RPL3
RPL31
RPL35A
RPL7A
RPL8
RPL9
RPSA
RUVBL1
RUVBL2
SAV1
SEC16A
SEC24B
SEC61A1
SENP1
SFXN4
SKIV2L2
SLAIN1
SLC25A1
SLC25A10
SLC25A12
SMARCA4
SMC1A
SMC3
SND1
SNRNP200
SON
SRSF3
SSBP1
SUGP2
TANC2
TCP1
TECR
TELO2
TIMM50
TOMM22
TP53
TRIM27
UBAP2L
UBE3C
UBR5
UNC45A
UPF1
USP9X
VCP
VIM
WDR33
WDR6
WDR77
XRCC5
XRCC6
XRN2
YTHDF2

μ2-P208S-eGFP (51)

ADNP
BAG2
CAMK2D
CAMK2G
CCAR2
CCT2
CCT5
CCT8
CDK1
CDKN2A
CPSF2
CYLD
DHFR
DPM1
EFTUD2
EIF4A1
EPRS
FAF2
GTF2I
GTF3C5
HADHA
HELZ
IKBKAP
LAS1L
MAGED1
MLF2
NDUFA13
NUP133
NUP153
PABPC1
PRKDC
PRPF8
PSMB2
PSMB3
PSMB4
PSMC2
RANBP2
RARS
RPL10A
SLAIN1
SLC25A1
SNRNP200
SNRPF
SON
SQSTM1
SRSF9
SUGP2
UBR5
VCP
VIM
WDR6

All four IP (19)

ADNP
BAG2
CCAR2
CCT2
CCT8
EFTUD2
GTF3C5
HELZ
LAS1L
NUP153
PABPC1
PRPF8
RANBP2
RPL10A
SLAIN1
SNRNP200
SON
VIM
WDR6

Three IP (eGFP-μ2, μ2-eGFP, eGFP-μ2-P208S, 12)

CCT4
CEP170
CKAP5
COPA
DDX21
DHX30
FAM120A
HERC2
MYCBP2
NEK1
POLDIP3
RPL9

Three IP (eGFP-μ2, eGFP-μ2-P208S, μ2-P208S-eGFP, 2)

HADHA
SUGP2

Three IP (μ2-eGFP, eGFP-μ2-P208S, μ2-P208S-eGFP, 6)

CCT5
IKBKAP
MAGED1
MLF2
NUP133
PRKDC

Two IP (μ2-eGFP eGFP-μ2, 5)

CCNT1
CDK9
CLASP2
PHLDB2
RSL1D1

Two IP (eGFP-μ2, eGFP-μ2-P208S, 14)

ATAD3B
CBX3
CSNK2A1
EXOSC10
FAM98B
GTSE1
HSP90AA1
IRS4
MRPS25
NEURL4
PKP4
RPL31
TANC2
XRCC6

Two IP (μ2-eGFP, eGFP-μ2-P208S, 22)

ABCD3
AKAP8
CCT7
CHD4
COPB2
CYC1
DDX1
DYNC1H1
EIF3C
EMD
MYBBP1A
MYO1C
NUP205
PGAM5
POLR2B
RPL7A
RUVBL2
SEC24B
SKIV2L2
UPF1
USP9X
XRN2

Two IP (μ2-eGFP, μ2-P208S-eGFP, 3)

CAMK2D
CAMK2G
CYLD

Two IP (eGFP-μ2-P208S, μ2-P208S-eGFP, 10)

DPM1
EIF4A1
EPRS
FAF2
GTF2I
PSMC2
RARS
SLC25A1
UBR5
VCP

Figure S21

Figure S22. STRING network of the 19 proteins identified in all IP-MS.

The interaction network was generated using the STRING database.

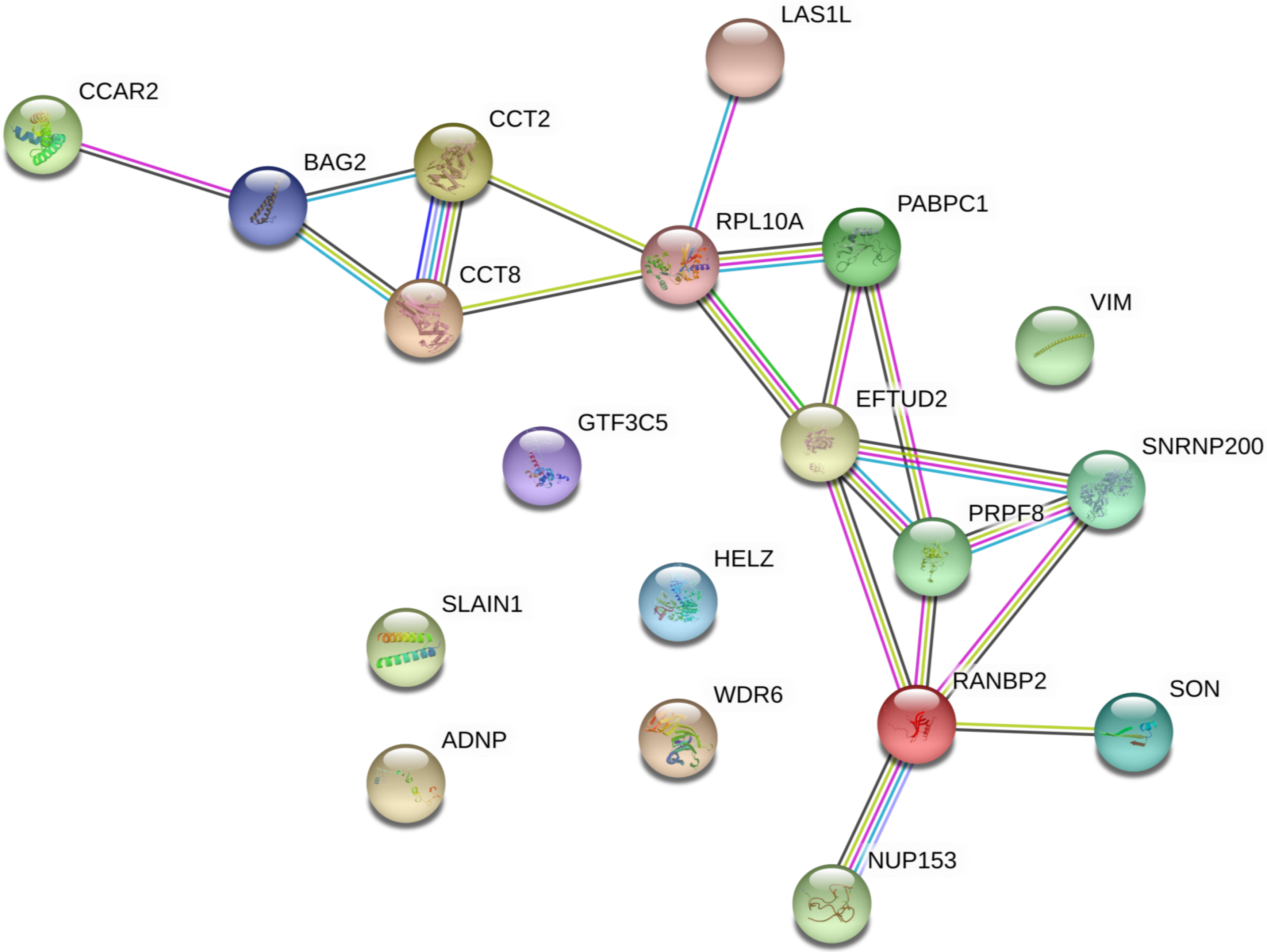
LAS1L
CCAR2
CCT2
BAG2
RPL10A
PABPC1
CCT8
VIM
EFTUD2
GTF3C5
SNRNP200
PRPF8
HELZ
SLAIN1
WDR6
RANBP2
SON
ADNP
NUP153

Figure S22

Figure S23. RIP-ddPCR of GFP, µ2-GFP and GFP-µ2 assessing the immunoprecipitation of all spliceosomal snRNA. 293T cells were transfected with pEGFPN1 (GFP), pEGFPN1-µ2 (µ2-GFP), or pEGFPC1-µ2 (GFP-µ2), and incubated for 24 h. RIP was performed as previously described (Boudreault et al. (2019) *Virology Journal* 16, pg. 29, reference 41 in the manuscript) and inputs and IP fractions were submitted to ddPCR. A percent of input immunoprecipitated was calculated based on quantity of the target before IP and after IP. n=3, biological replicates, unpaired two-tailed Student's t-test (ns, P>0.05; *, P≤0.05; **, P≤0.01) against the GFP alone IP. Input fractions were normalized using the MRPL19 housekeeping gene

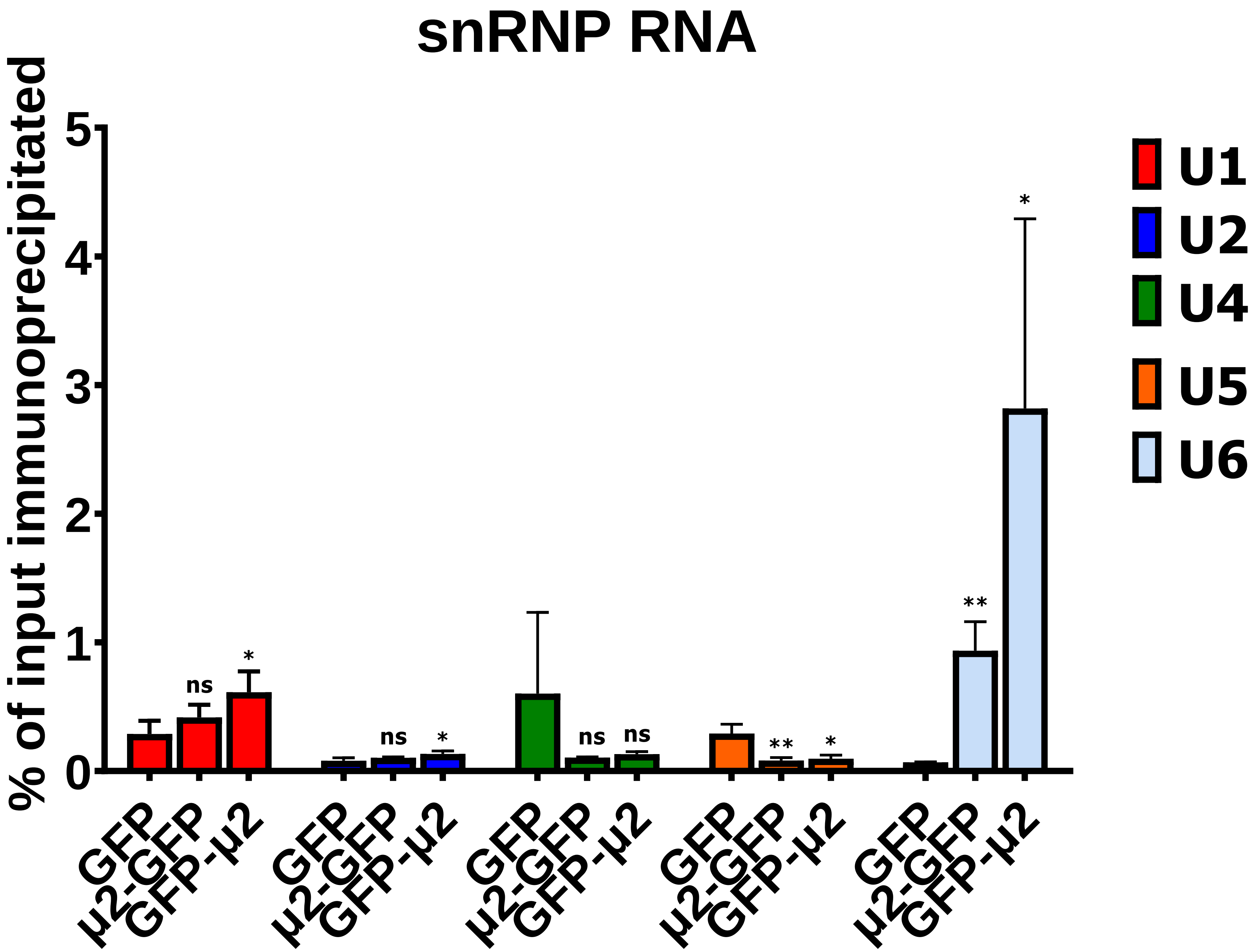

snRNP RNA
% of input immunoprecipitated
0
1
2
3
4
5
ns
*
ns
*
ns
ns
**
*
**
*
GFP
μ2-GFP
GFP-μ2
GFP
μ2-GFP
GFP-μ2
GFP
μ2-GFP
GFP-μ2
GFP
μ2-GFP
GFP-μ2
GFP
μ2-GFP
GFP-μ2
U1
U2
U4
U5
U6

Figure S23

Figure S24. Relative mRNA levels for M1 and S1 viral genes during infection upon silencing of EFTUD2, PRPF8 and SNRNP200. L929 cells were transfected with the respective siRNA using RNAiMAX, and 56 h post-transfection, cells were infected with MRV ($T3D^S$) at a MOI of 50. Cells were further incubated for 16 h before RNA was harvested using Qiazol, reverse-transcribed, and subjected to qPCR for the S1 and M1 viral gene segment with PSMC4, PUM1, and TXNL4B as housekeeping genes for normalization. The first replicate in the siCTRL condition was fixed at 1 and the relative mRNA expression was calculated for all other samples relative to that one. n=3, biological replicates, unpaired two-tailed Student's t-test (ns, $P>0.05$; *, $P\leq0.05$) against the siCTRL condition.

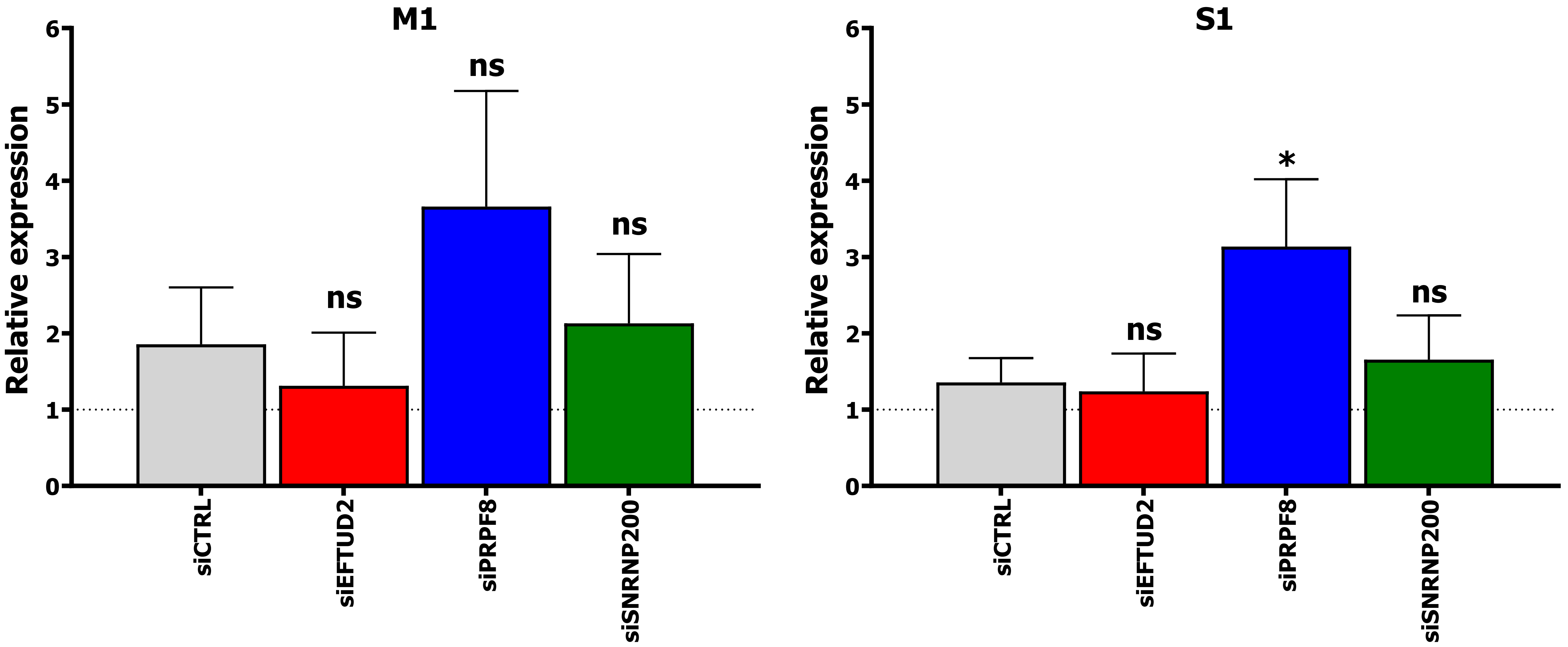
M1
Relative expression
0
1
2
3
4
5
6
ns
ns
ns
siCTRL
siEFTUD2
siPRPF8
siSNRNP200
S1
Relative expression
0
1
2
3
4
5
6
ns
*
ns
siCTRL
siEFTUD2
siPRPF8
siSNRNP200

Figure S24

Figure S25. Viral protein levels of μ2 and σ3 during infection upon silencing of EFTUD2, PRPF8 and SNRNP200. L929 cells were transfected with the respective siRNA using RNAiMAX, and 56 h post-transfection, cells were infected with MRV ($T3D^S$) at a MOI of 50. Cells were further incubated for 16 h before being lysed in RIPA. Protein lysates were dosed by Bradford assay, and western blots against σ3 and μ2 were realized. Membranes were H_2O_2-inactived and probed against the loading controls actin (μ2) and GAPDH (σ3). The relative protein level was calculated using Image J.

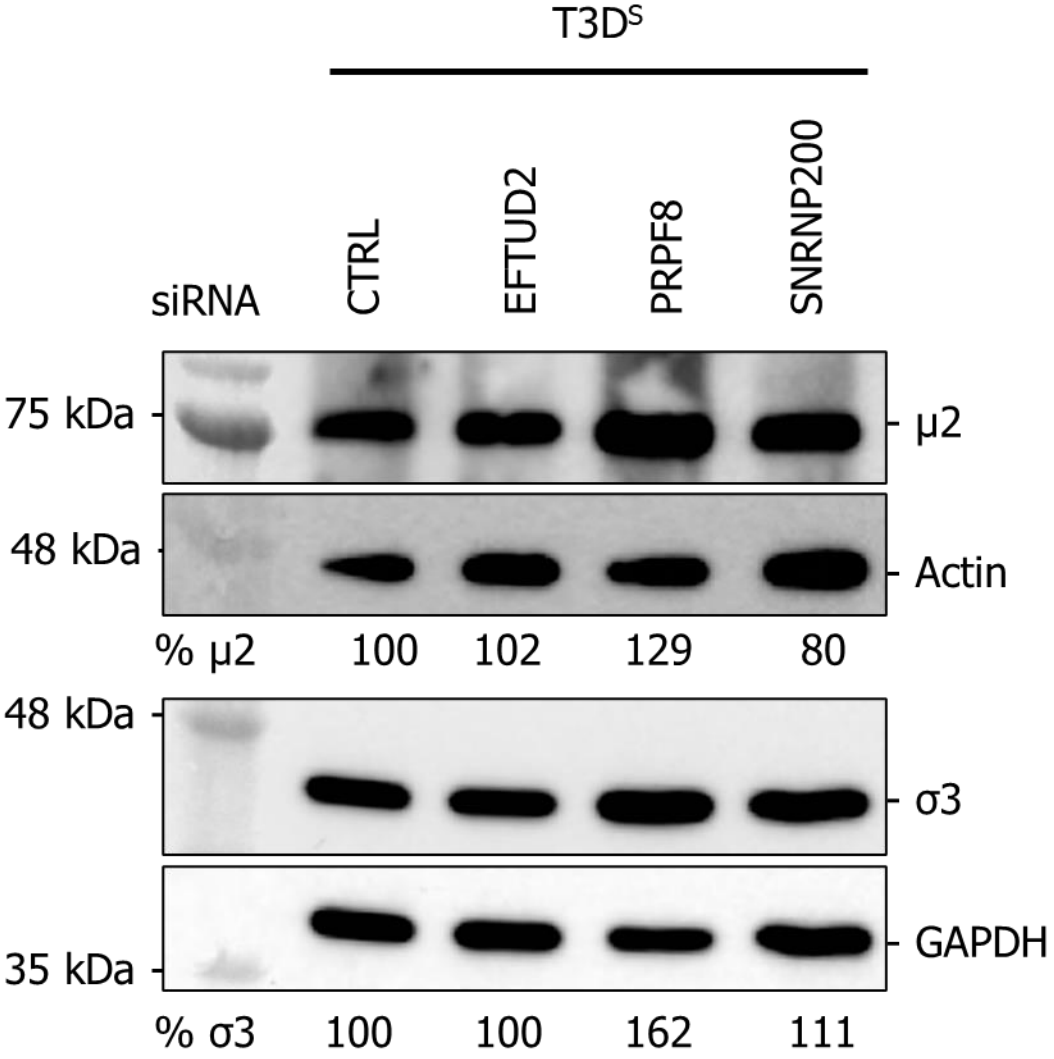

T3D^S
siRNA
CTRL
EFTUD2
PRPF8
SNRNP200
75 kDa
μ2
48 kDa
Actin
% μ2 100 102 129 80
48 kDa
σ3
GAPDH
35 kDa
% σ3 100 100 162 111

Figure S25

Figure S26. Impact of silencing core U5 proteins EFTUD2, PRPF8, and SNRNP200 on cellular AS. L929 cells were transfected with the respective siRNA using RNAiMAX, and 56 h post-transfection, cells were mock-infected, and further incubated for 16 h before RNA was harvested using Qiazol, reverse-transcribed, and subjected to AS-PCR for the different ASE analyzed. PCR amplicons were resolved using capillary electrophoresis and quantified using relative fluorescence. n=3, biological replicates, unpaired two-tailed Student's t-test (ns, $P>0.05$; *, $P\leq0.05$; **, $P\leq0.01$; ***, $P\leq0.001$; ****, $P\leq0.0001$) against the control siRNA condition.

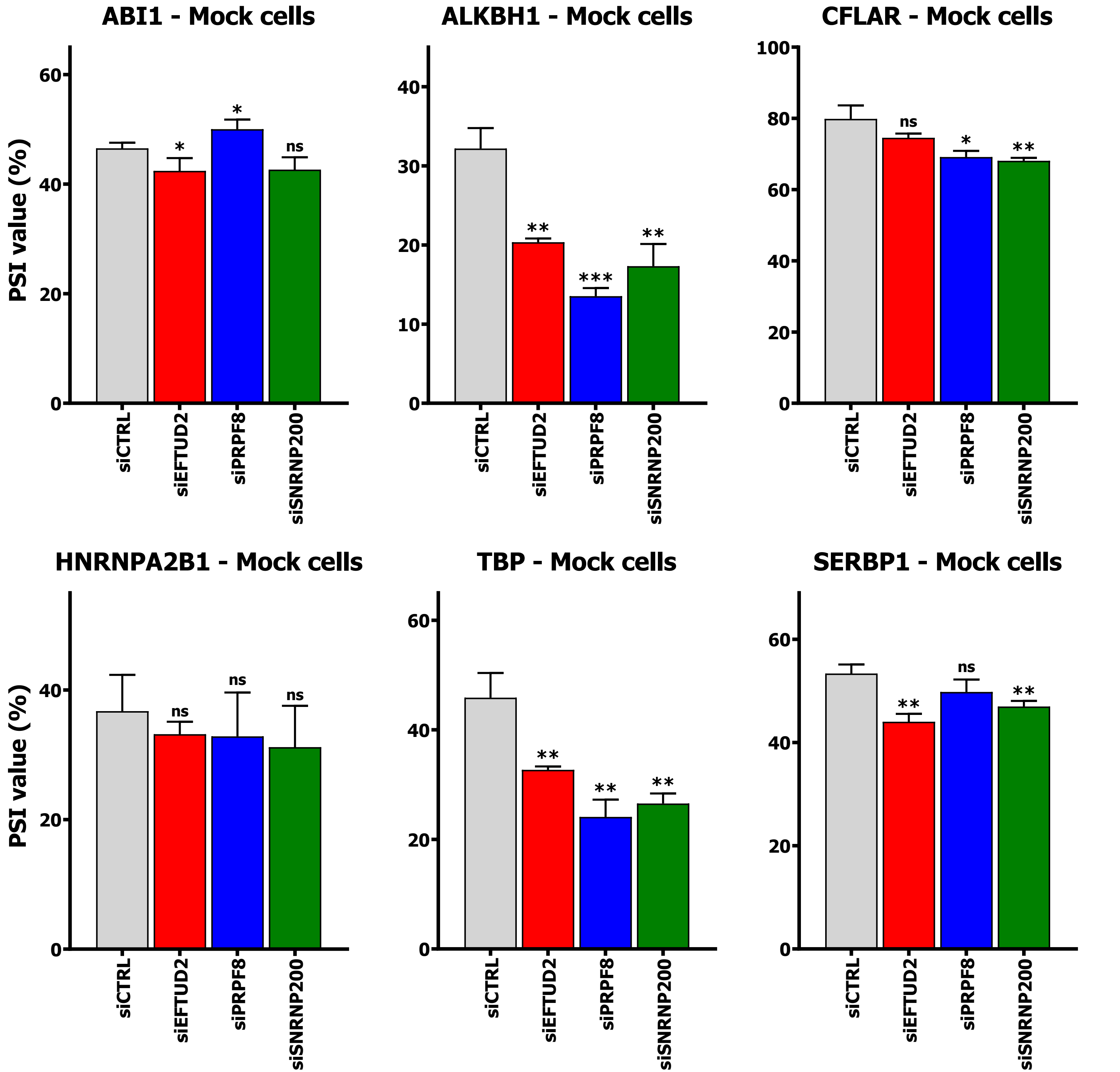
ABI1 - Mock cells
ALKBH1 - Mock cells
CFLAR - Mock cells
HNRNPA2B1 - Mock cells
TBP - Mock cells
SERBP1 - Mock cells
PSI value (%)
siCTRL
siEFTUD2
siPRPF8
siSNRNP200
ns
*
**
***

Figure S26

Figure S27. Immunofluorescence of U5 spliceosomal components EFTUD2, SNRNP200, and PRPF8 in mock-, T3D^{S}- and T3D^{S} M1-P208S-infected cells. L929 cells were infected at a MOI of 50, fixed at 16 h post-infection and imaged using protein-specific antibodies against EFTUD2, SNRNP200, and PRPF8 using standard immunofluorescence technique. Slides were imaged using a confocal Zeiss LSM 880 2-Photon microscope. The scale bars represent 20 μm.

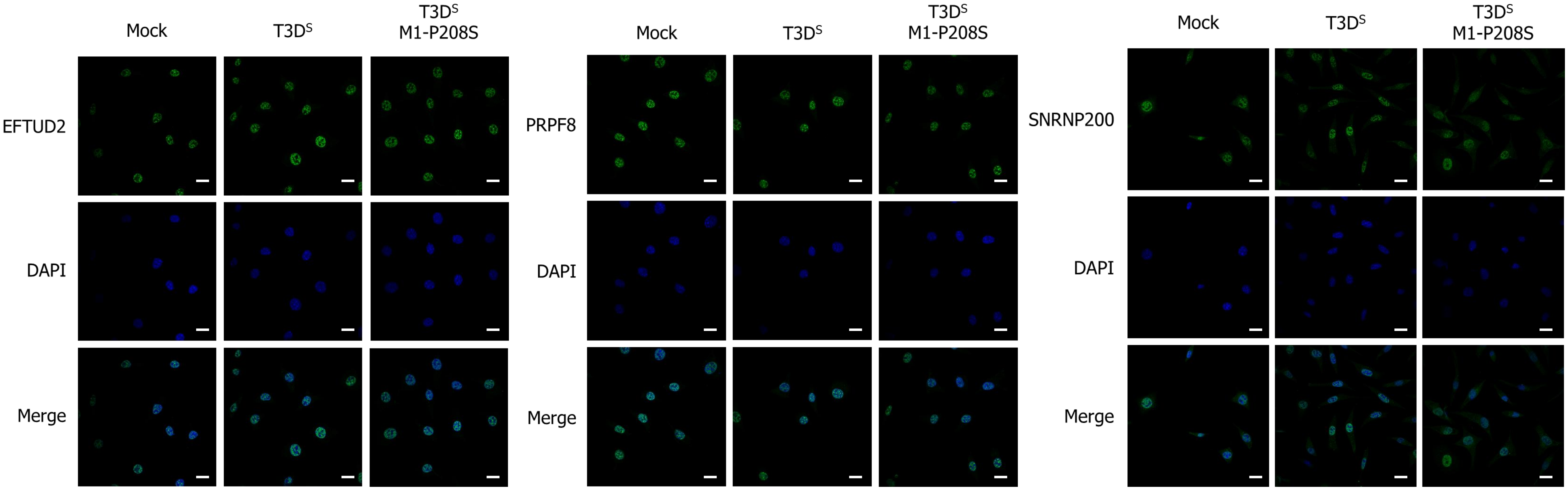

Figure S27

Figure S28. Relative RNA level for all spliceosomal snRNA upon infection with T3D^S and T3D^K. L929 cells were mock-infected or infected with MRV (T3D^S or T3D^K) at a MOI of 50, incubated for 16 h before RNA was harvested using Qiazol, reverse-transcribed, and subjected to qPCR for U1, U2, U4, U5, and U6 snRNA with PSMC4, PUM1, and TXNL4B used as housekeeping genes. The first replicate in the mock siCTRL condition was fixed at 1, and the relative mRNA expression was calculated for all other samples relative to that one. n=3, biological replicates, one-way ANOVA with Dunnett's multiple comparisons test against the mock condition (ns, $P>0.05$; *, $P\leq0.05$; **, $P\leq0.01$).

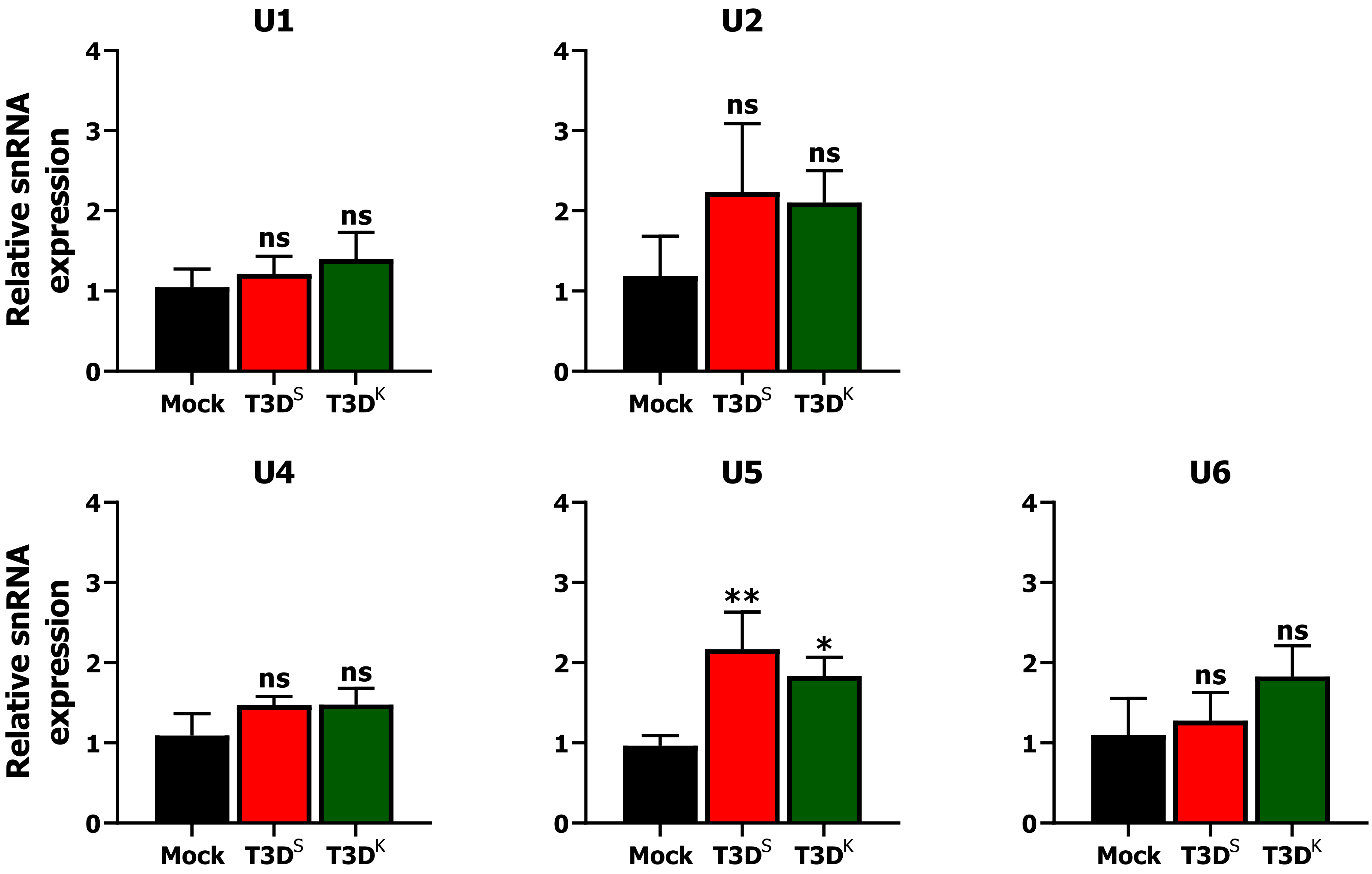
U1
U2
U4
U5
U6
Relative snRNA expression
0
1
2
3
4
Mock
T3D^S
T3D^K
ns
**
*
Figure S28

Figure S29. Relative quantitation of U5 snRNP protein levels upon expression of different µ2-GFP constructs. 293T cells were transfected with pEGFPN1 (GFP), pEGFPN1-µ2 (µ2-GFP), pEGFPC1-µ2 (GFP-µ2), or P208S mutants and incubated for 24 h or 48 h before being lysed in RIPA. Protein lysates were dosed by Bradford assay, and western blots against EFTUD2, PRPF8, SNRNP200 or U2AF35 were realized. The membranes were H_2O_2-inactived and probed again against actin (EFTUD2, U2AF35) or vinculin (PRPF8, SNRNP200) as a loading control. The cumulative results for three western blots are summarized in bar graph. The U2 auxiliary factor U2AF35 was probed as a control. n=3, biological replicates, unpaired two-tailed Student's t-test (ns, $P>0.05$; *, $P\leq0.05$; **, $P\leq0.01$; ***, $P\leq0.001$) comparing against GFP alone control cells.

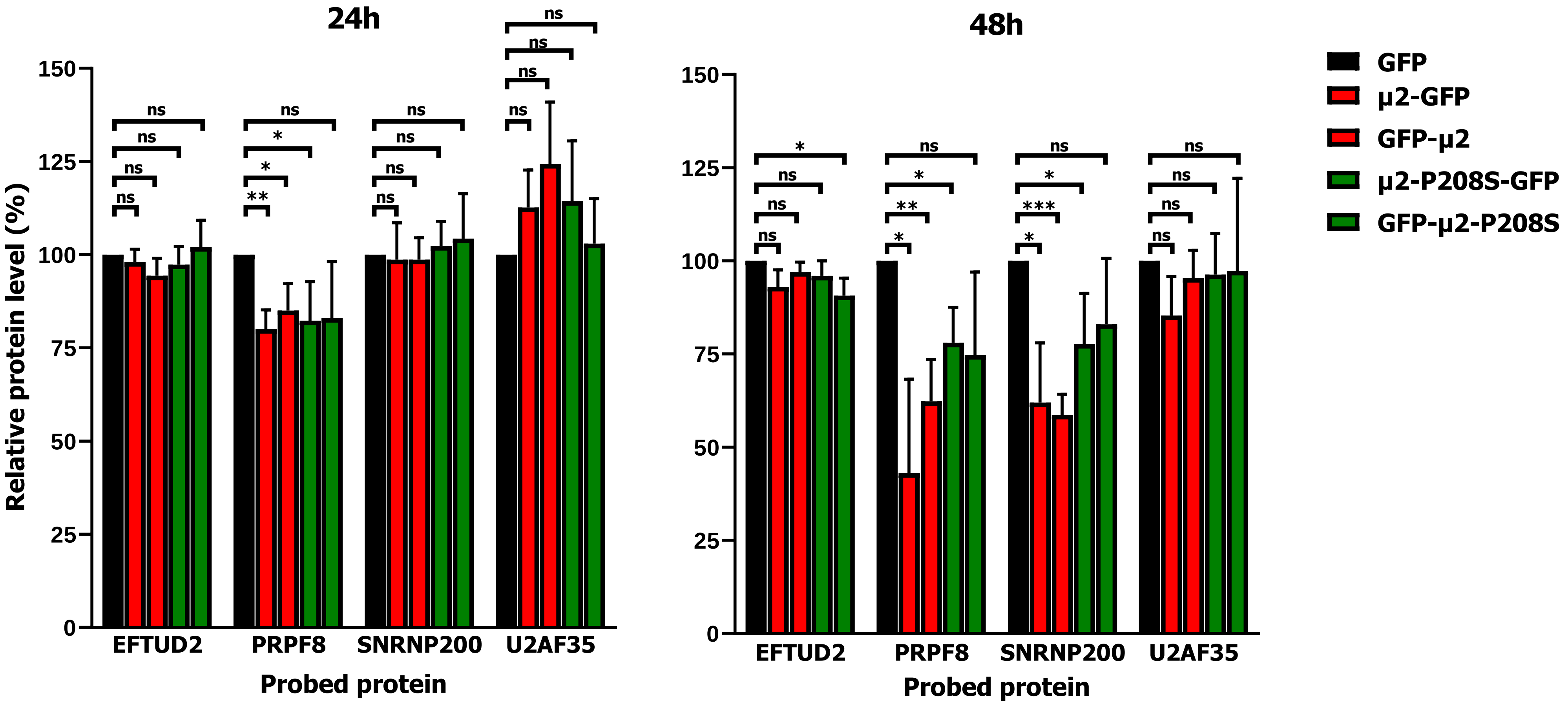
24h
48h
Relative protein level (%)
150
125
100
75
50
25
0
EFTUD2
PRPF8
SNRNP200
U2AF35
Probed protein
ns
*
**
***
GFP
µ2-GFP
GFP-µ2
µ2-P208S-GFP
GFP-µ2-P208S

Figure S29

Figure S30. RIP-ddPCR of GFP, μ2-GFP, and GFP-μ2 assessing the immunoprecipitation of cellular mRNA. 293T cells were transfected with pEGFPN1 (GFP), pEGFPN1-μ2 (μ2-GFP), or pEGFPC1-μ2 (GFP-μ2), and incubated for 24 h. RIP was performed as previously described (Boudreault et al. (2019) *Virology Journal* 16, pg. 29, reference 41 in the manuscript) and both input and IP fractions were submitted to ddPCR. A percent of input immunoprecipitated was calculated based on quantity of the target before IP and after IP. Input fractions were normalized using the MRPL19 housekeeping gene. n=3, biological replicates, unpaired two-tailed Student's t-test(*, $P \leq 0.05$; **, $P \leq 0.01$; ***, $P \leq 0.001$; ****, $P \leq 0.0001$) against the GFP alone IP.

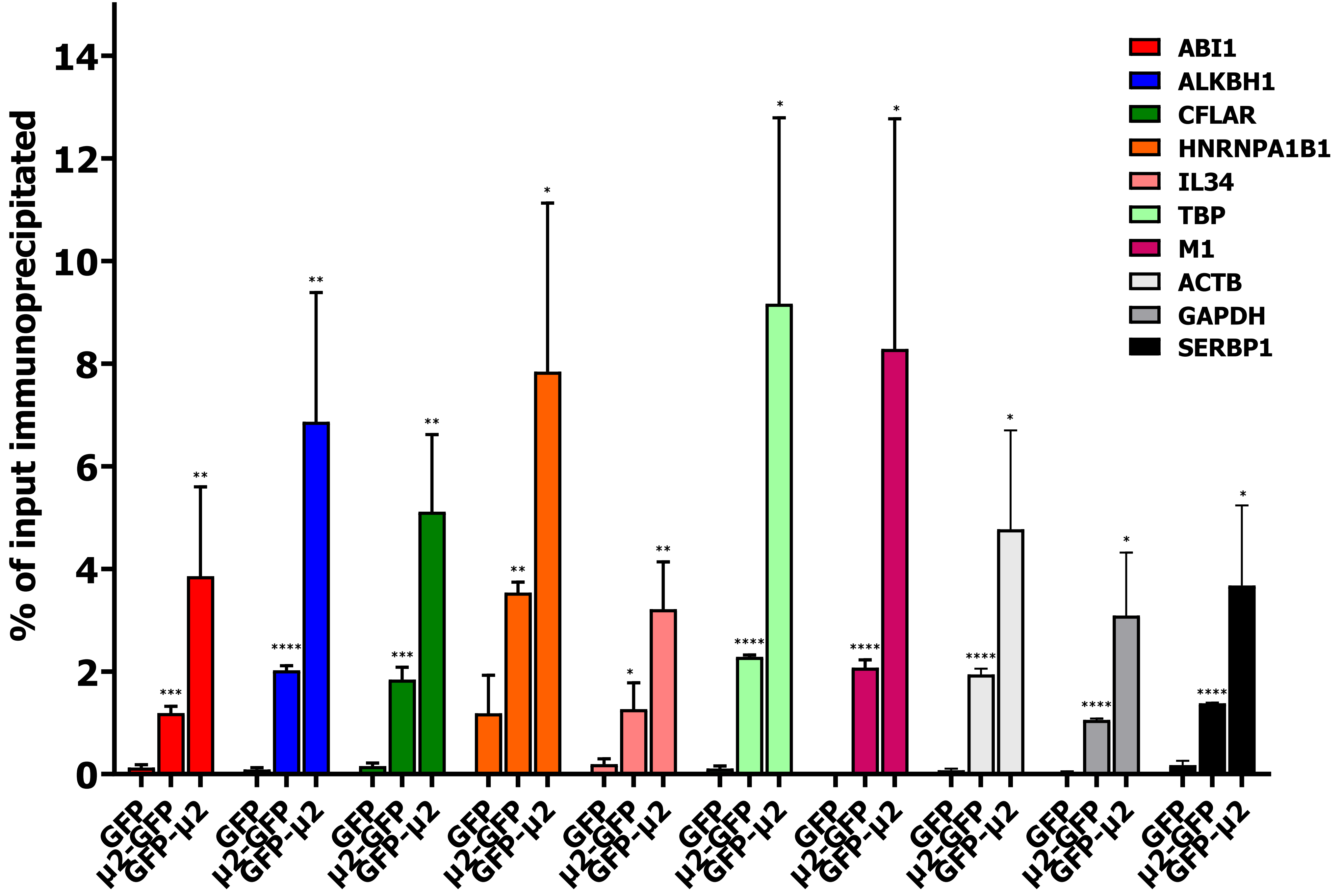

% of input immunoprecipitated
0
2
4
6
8
10
12
14
ABI1
ALKBH1
CFLAR
HNRNPA1B1
IL34
TBP
M1
ACTB
GAPDH
SERBP1
GFP
μ2-GFP
GFP-μ2

Figure S30

Figure S31. μ2 protein levels in all *wild-type*/reassortant/single amino acid mutant MRV viruses. L929 cells were infected with the respective virus at a MOI of 50 and incubated for 16 h before being lysed in RIPA. Protein lysates were dosed by Bradford assay and a western blot against μ2 was realized. The membrane was H_2O_2-inactived and probed against the loading control actin. Relative protein level was calculated using Image J.

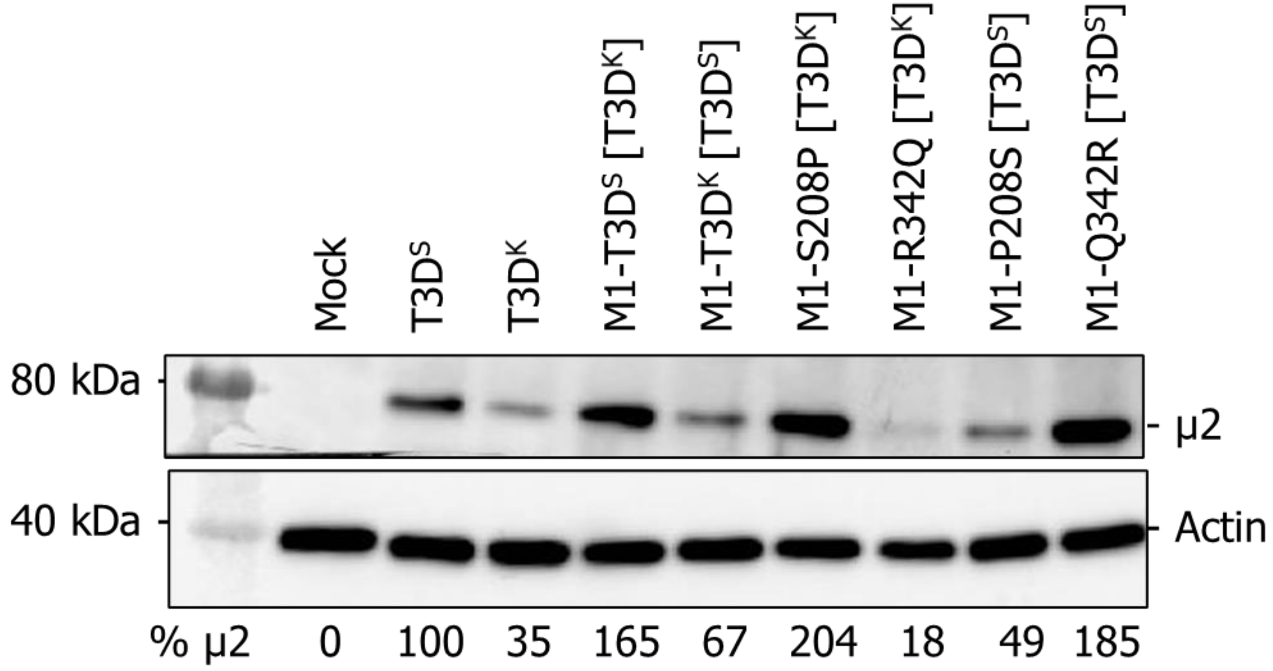
Mock
T3DS
T3DK
M1-T3DS [T3DK]
M1-T3DK [T3DS]
M1-S208P [T3DK]
M1-R342Q [T3DK]
M1-P208S [T3DS]
M1-Q342R [T3DS]
80 kDa
40 kDa
μ2
Actin
% μ2
0
100
35
165
67
204
18
49
185

Figure S31

Figure S32. Uncropped western blots from this study.

Figure 1A

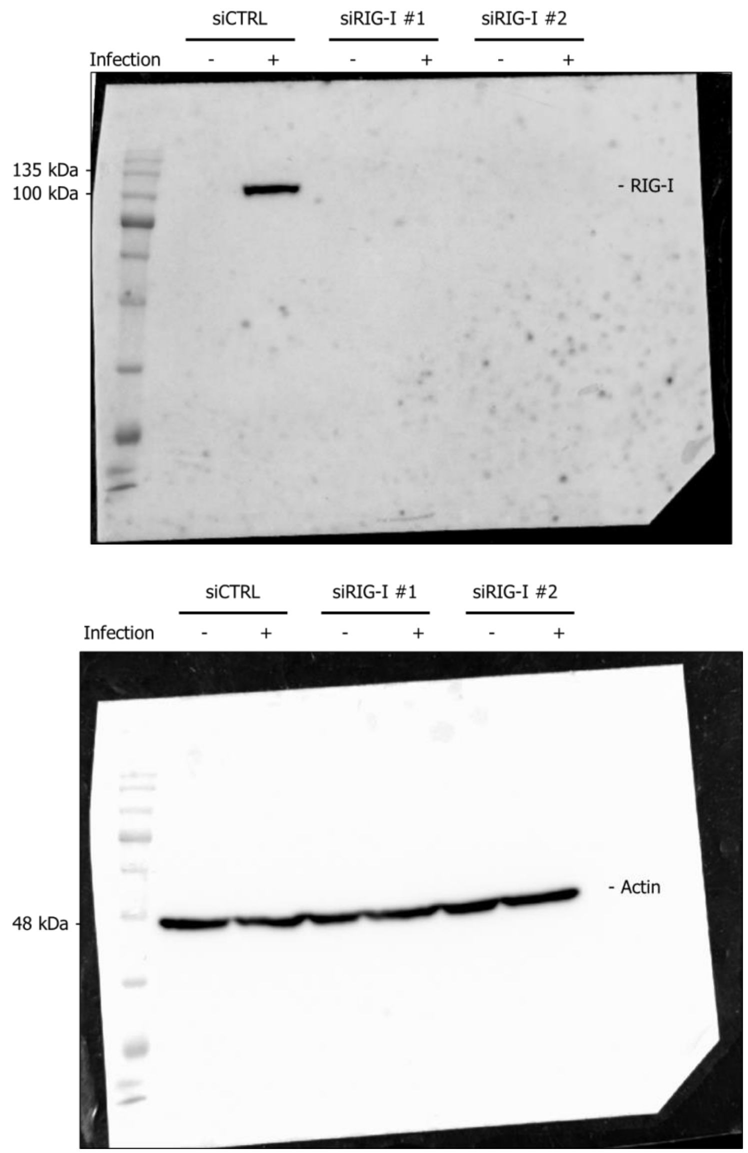

Figure 5A

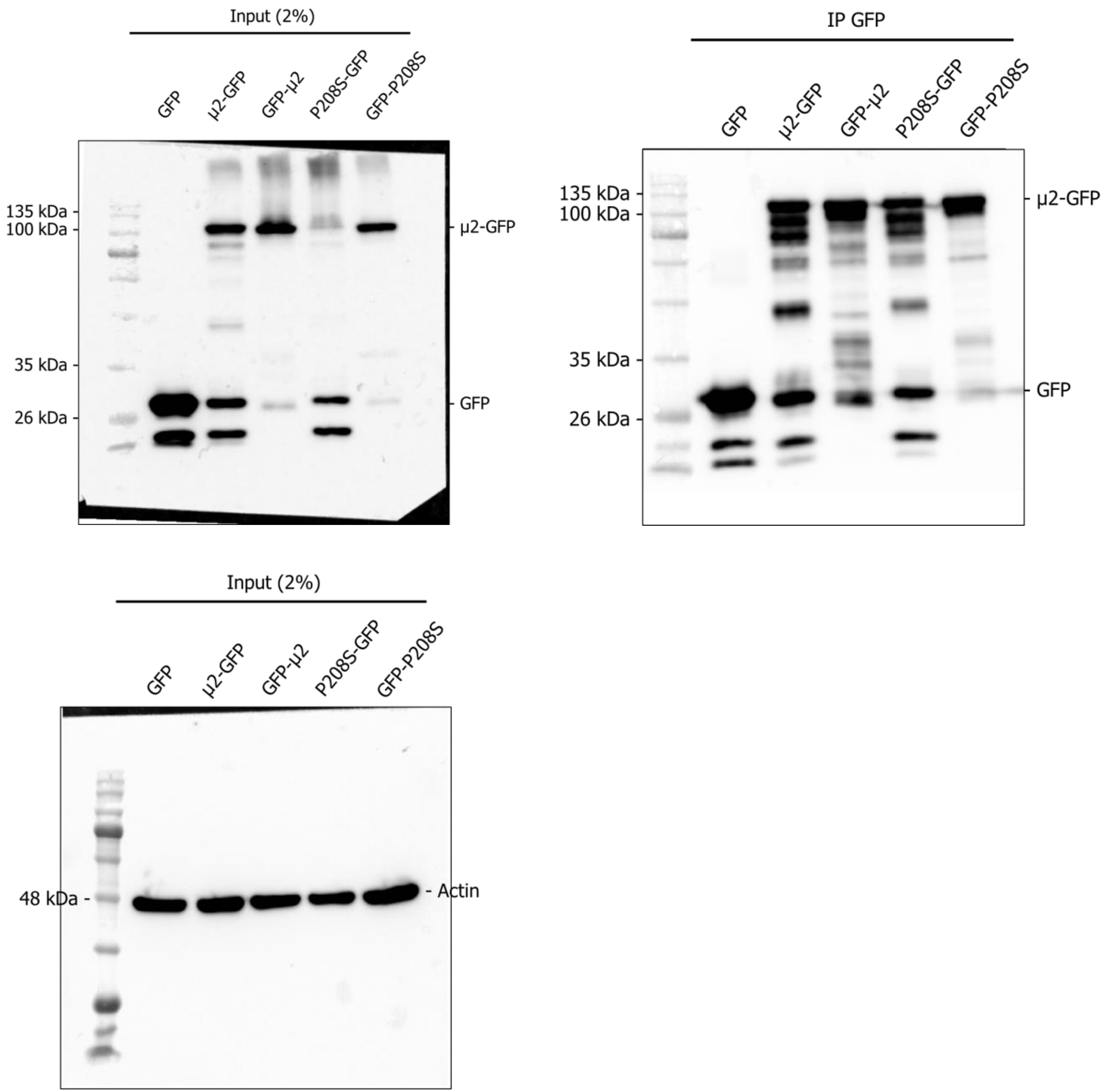

Figure S32

Figure 5C

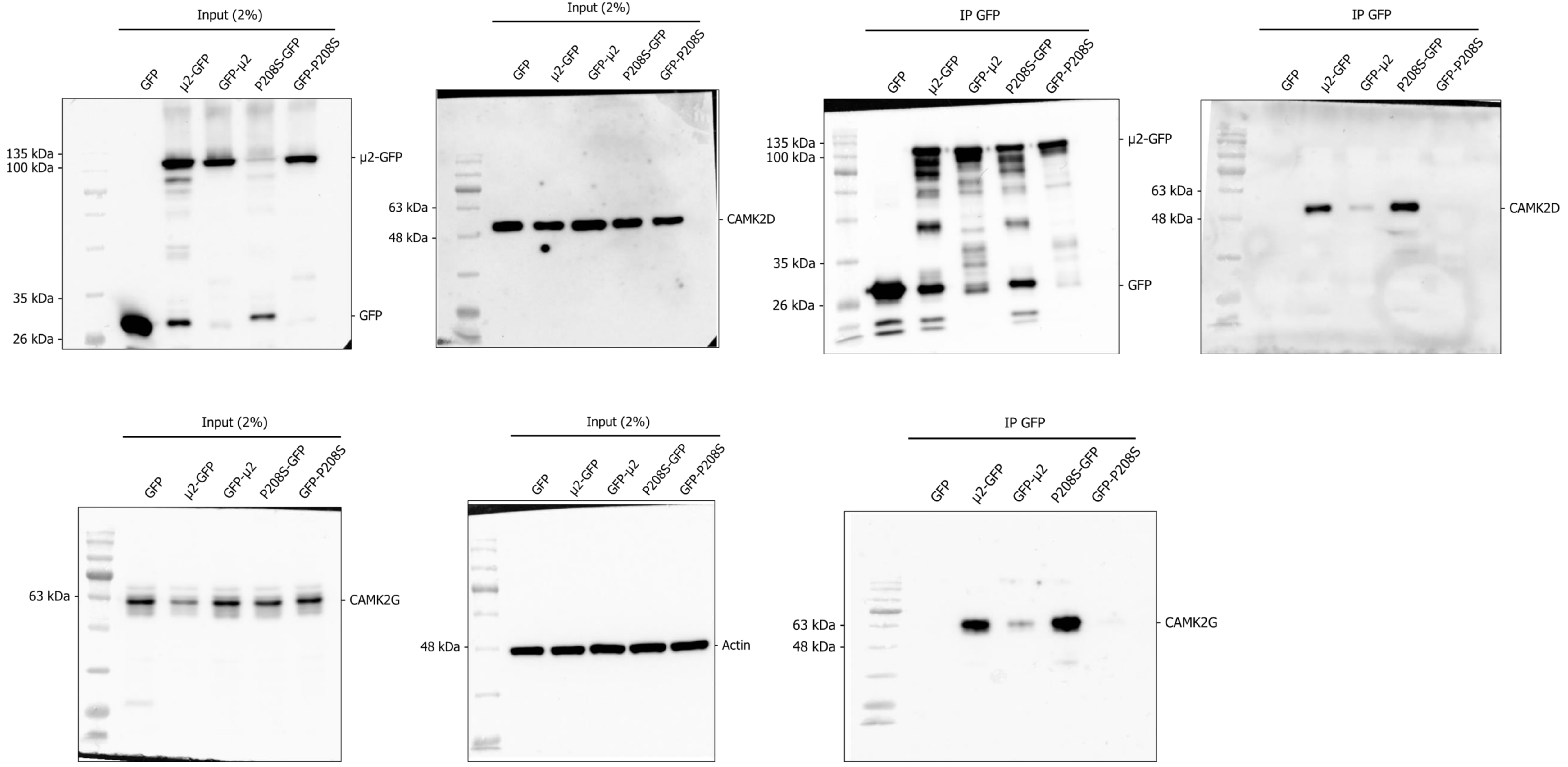

Figure S32 (cont'd)

Figure 5D

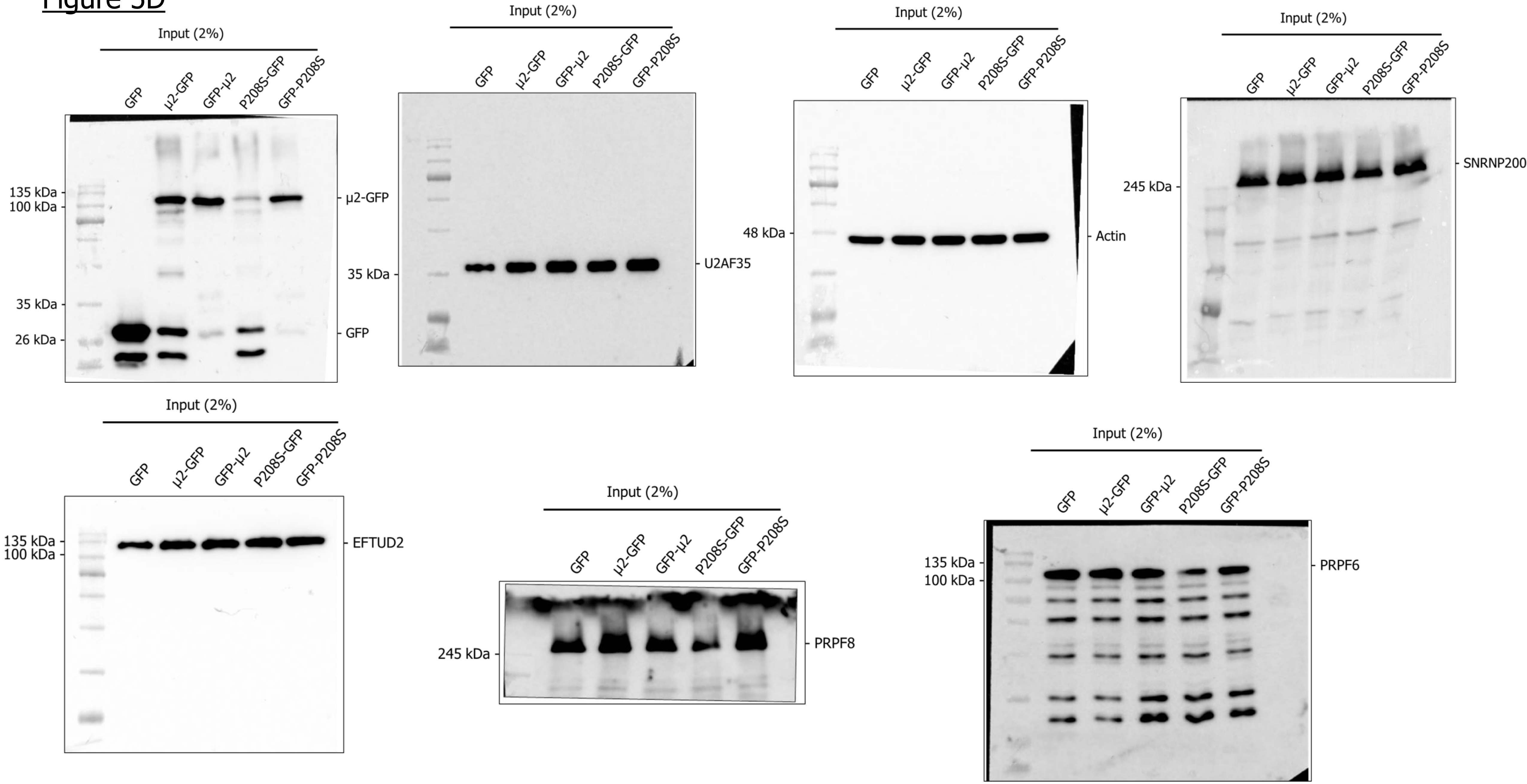

Figure S32 (cont'd)

Figure 5D (cont'd)

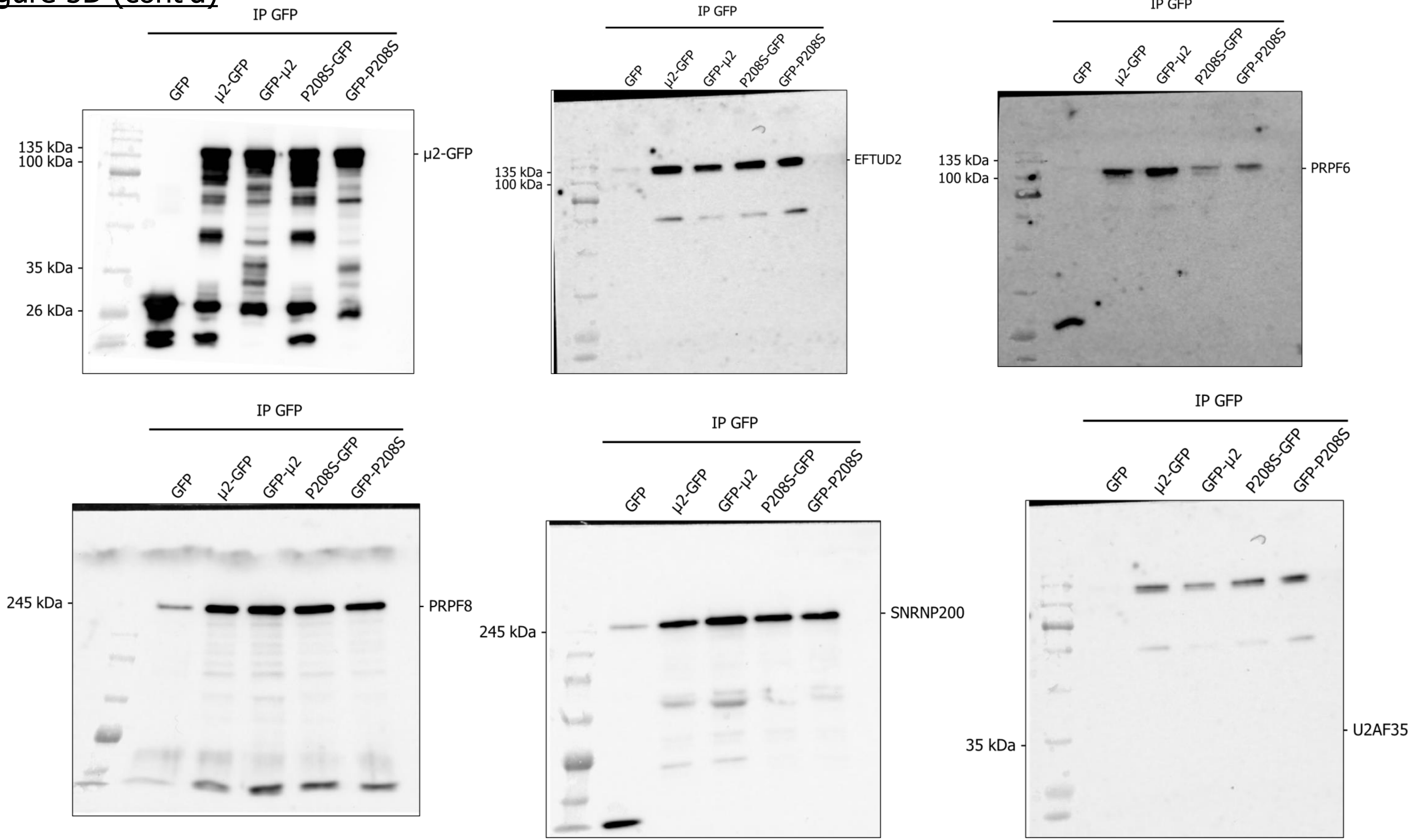

Figure S32 (cont'd)

Figure 6A

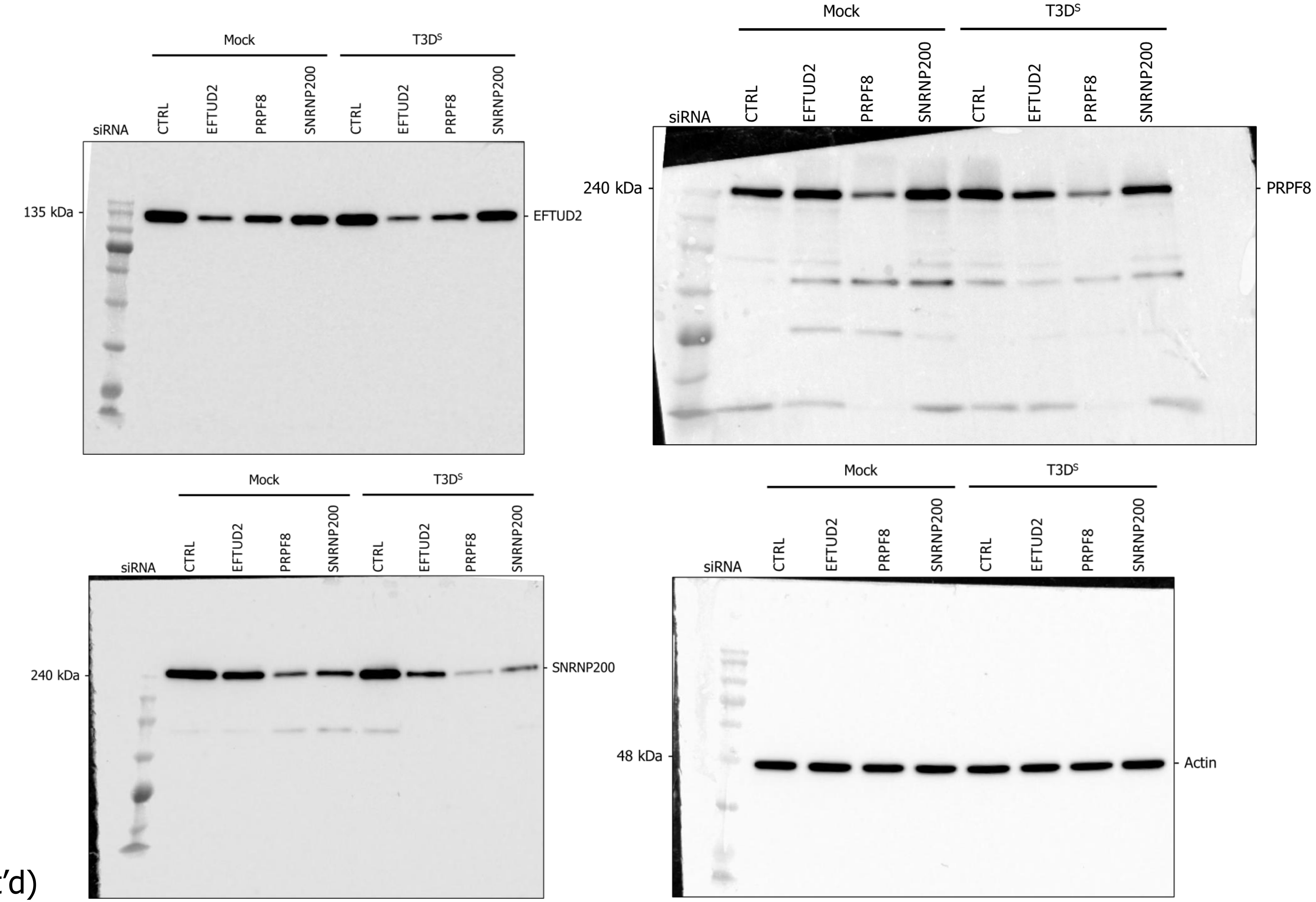

Figure S32 (cont'd)

<u>Figure 6C</u>

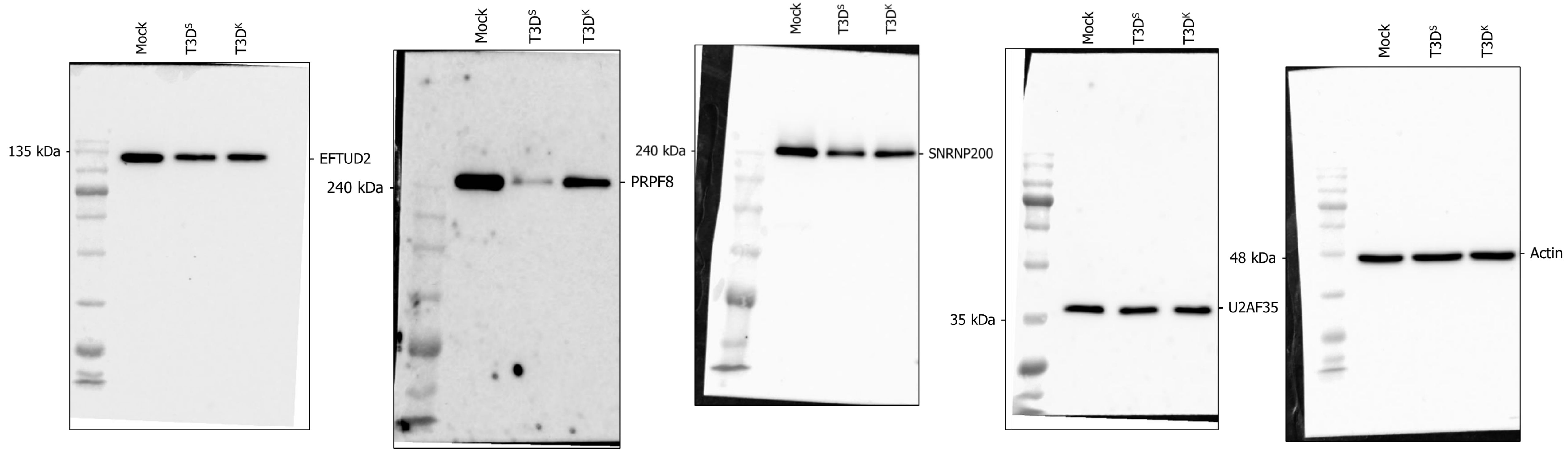

Figure S32 (cont'd)

Figure 6D

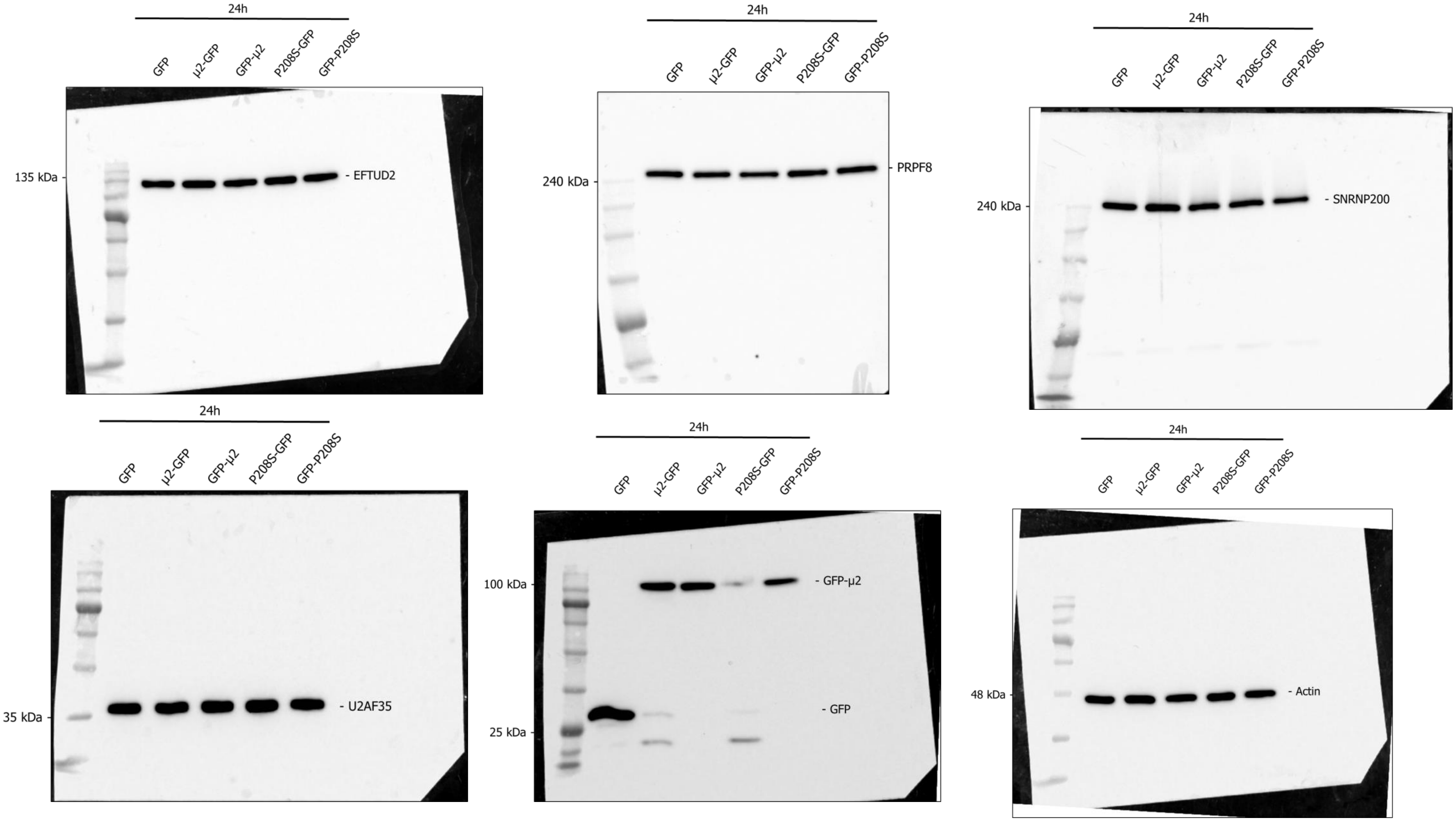

Figure S32 (cont'd)

Figure 6D (cont'd)

Figure S32 (cont'd)

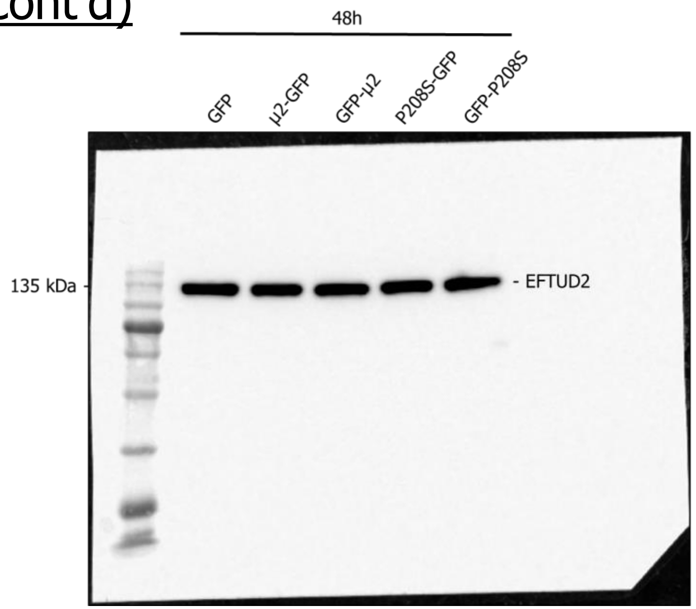

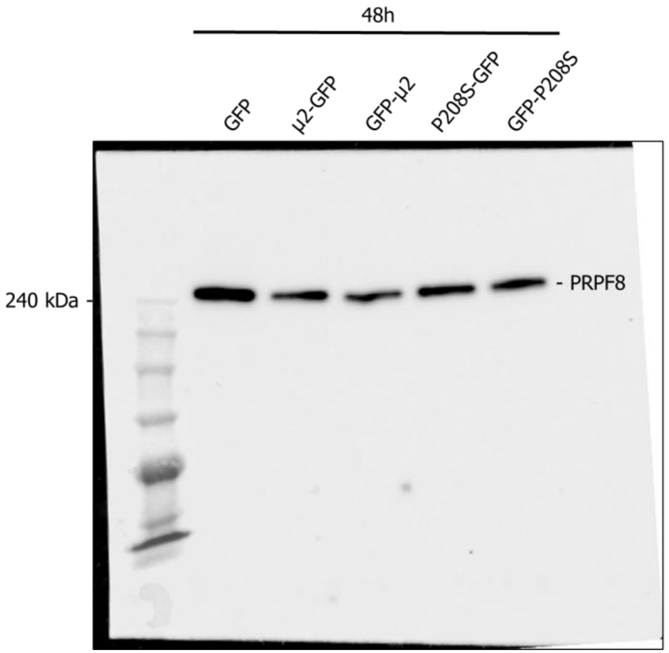

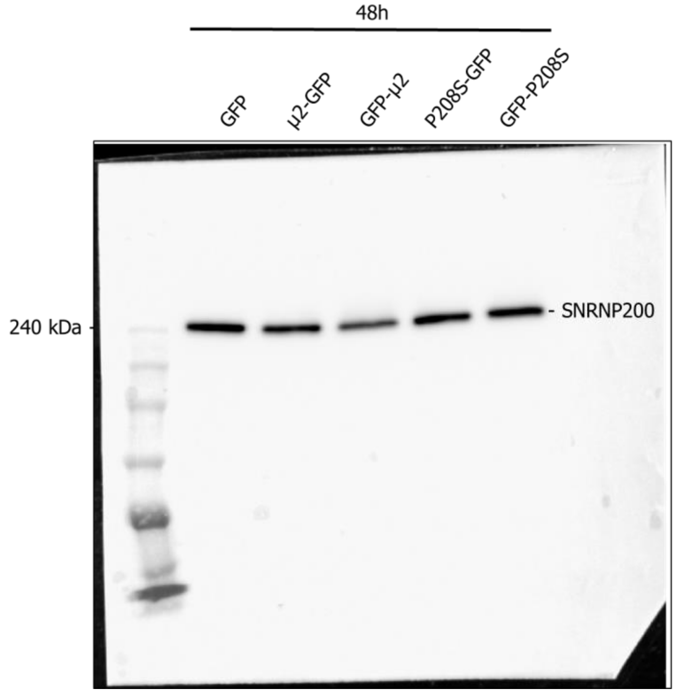

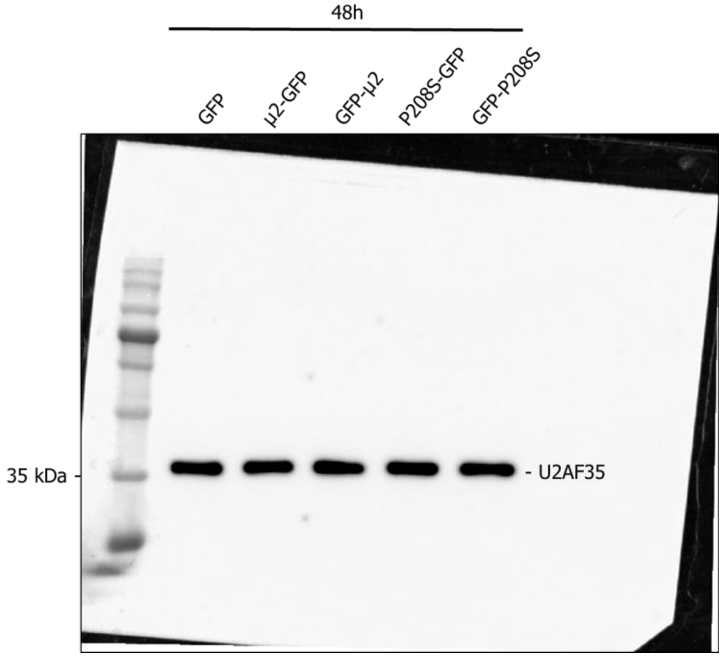

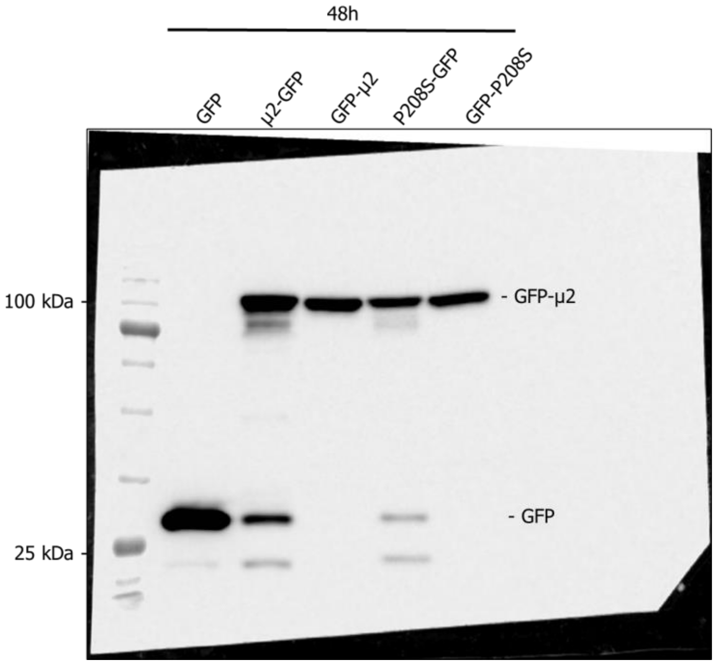

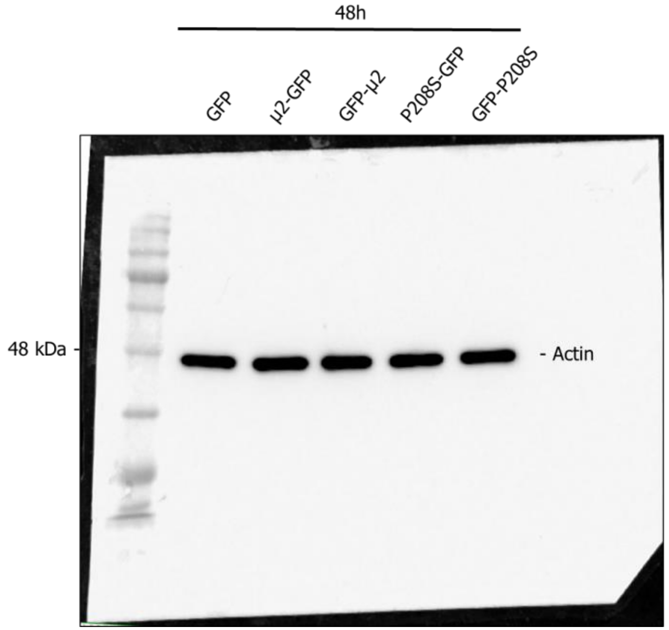

Supplement: gkac272_Supplemental_File [file gkac272_supplemental_file.pdf]
